# Early-life exposures and risk of hematological malignancies in adulthood: a cohort study, meta-analysis and Mendelian randomization analysis

**DOI:** 10.1186/s12885-025-14780-y

**Published:** 2025-10-08

**Authors:** Yuelin Guo, Jing Zheng, Hongli Huang, Lili Pan, Haomin Yang

**Affiliations:** 1 Department of Epidemiology and Health Statistics, School of Public Health & Fujian Provincial Key Laboratory of Environment Factors and Cancer, Fujian Medical University, Xue Yuan Road 1, University Town, Fuzhou, Fujian 350122 China; 2 Fuqing Center for Disease Control and Prevention, Fuzhou, 350300 China; 3 Department of Hematology, Fujian Institute of Hematology, Fujian Medical University Union Hospital, Xinquan Road, No.29, Fuzhou, Fujian China; 4 Department of Medical Epidemiology and Biostatistics, Karolinska Institutet, Stockholm, 17177 Sweden

**Keywords:** Early-life exposures, Adults hematological malignancy, Mendelian randomization

## Abstract

**Background:**

Despite the global increase in annual incidence of hematological malignancies, the associations between early-life exposures and adult hematological malignancies were not well understood.

**Methods:**

By conducting a prospective cohort study in the UK biobank, hazard ratios (HR) and 95% confidence intervals (CI) for hematological malignancies based on early-life exposures were estimated with Cox regression models. We further conducted a meta-analysis combining our study with previous cohort studies, and a two-sample Mendelian randomization (MR) analysis to evaluate the potential causal association.

**Results:**

Smoking initiation < 14 years old, a larger body size and taller in height at age 10, were associated with an increased risk of hematological malignancies in adulthood, with corresponding HRs 1.17 (95% CI = 1.03; 1.32), 1.16 (95%CI = 1.06; 1.27) and 1.24 (95%CI = 1.14; 1.36), respectively. The associations were mediated by adulthood BMI and height, but not smoking status. Meta-analysis found no significant association for maternal smoking around birth, multiple births and breastfed as a baby. In addition, the MR analysis suggested a potential causal effect of height at age 10 and age at smoking initiation on leukemia (*p* < 0.05).

**Conclusions:**

Taller height at age 10 and younger age at smoking initiation were associated with an increased risk of hematological malignancies in adulthood, especially for leukemia. These findings emphasize the importance of comprehensive tobacco control among adolescents and inclusion of these factors in the assessment for hematological malignancies risk.

**Supplementary Information:**

The online version contains supplementary material available at 10.1186/s12885-025-14780-y.

## Introduction

Hematological malignancies, including leukemia, lymphoma and multiple myeloma (MM), account for approximately 7% of global incident cancer cases annually, and the incidence rate is still rising [1]. Several large-scale studies have identified potential risk factors for hematological malignancies, such as anthropometric characteristics, physical activity [2], alcohol consumption [3], and family history [4]. However, the exact etiology remains elusive. Given the long latency of cancer, it is plausible that early-life exposures (e.g. birth characteristics and childhood lifestyles) may play a role in initiating carcinogenesis process for hematological malignances, especially for early-onset cases. While there is evidence linking early-life exposures to childhood hematological malignancy [5–7], limited research exists regarding their influence on adult hematological malignancy, which is more prevalent in the general population [8].

Previous research has shown the effect of birth characteristics on adulthood obesity, as well as the association between adulthood obesity and risk of hematological malignancies [9]. Nonetheless, direct evidence linking birth characteristics, such as multiple births and birthweight > 4000 g, to hematological malignancies in adulthood is limited due to small sample sizes or within a selected population in previous cohorts [10–12].

Childhood lifestyle factors may also influence the risk of hematological malignancies in adulthood. Childhood weight and height have been identified as predictors for certain hematological malignancies, such as non-Hodgkin lymphoma, and acute myeloid leukemia [12–14], while their impact on other hematological malignancies is still unknown. Exposure to tobacco smoking during intrauterine development and childhood [15, 16] may increase the risk and mortality rate of cancer, though the association with hematological malignancies requires further research.

This study aims to investigate the associations between early-life exposures and the risk of hematological malignancies in adulthood in a large-scale community-based cohort, with particular focus on birth characteristics and childhood lifestyles. We also attempt to validate our findings through meta-analysis and Mendelian randomization analysis.

## Methods

A prospective cohort study was conducted using the UK Biobank cohort (UKB), which recruited over 0.5 million participants aged 40–70 years between 2006 and 2010 from 22 assessment centers in England, Scotland and Wales. A wide range of data including socioeconomic status, life-style factors and environmental exposures was collected through self-administered touchscreen questionnaires at the assessment centers. UKB has been reviewed by the North West Multi-center Research Ethics Committee and written informed consent was obtained from all participants.

### Ascertainment of birth characteristics and lifestyle factors in childhood and adulthood

At the assessment center, the participants reported their birth characteristics through the questionnaires, including maternal smoking around birth, birth weight, multiple birth status, and being breastfed. Maternal smoking around birth was ascertained by the question “Did your mother smoke regularly around the time when you were born?”. Part of a multiple birth was determined by the question “Are you a twin, triplet or other multiple birth?”. Whether the participants were breastfed as a baby was ascertained using the question “Were you breastfed when you were a baby?”. These characteristics were dichotomized by “Yes” or “No”, and set as missing if the participants answered “Do not know” or “Prefer not to answer”. In the verbal interview, participants also provided their birth weight if known, and birth weight was categorized according to World Health Organization guidelines as < 2.5, 2.5 ~ 4 and > 4 kg. Information on these early-life exposures from questionnaires has been used in previous publications [17, 18] and displayed on the UK Biobank website (https://biobank.ndph.ox.ac.uk/showcase/ukb/docs/TouchscreenQuestionsMainFinal.pdf).

For childhood lifestyle factors, comparative body and height size at age 10 were collected through the question “When you were 10 years old, compared to average, would you describe yourself as…”. Participants’ body size was categorized as thinner, larger, or about average, while their height size was categorized as shorter, taller, or about average. Participants who answered “Do not know” or “Prefer not to answer” had their responses set as missing as well. Age at smoking initiated was defined as < 14, 15–18 and > 18 years old according to the suggestion from a previous publication [19].

Adulthood lifestyle factors were collected during UK Biobank baseline assessments. Current smoking status was self-reported via standardized touchscreen questionnaires, while BMI and height were objectively measured by trained staff using calibrated instruments.

### Outcome ascertainment

The diagnosis of hematological malignancy was obtained through linkage to the National Health Service (NHS), using personal identification numbers. Different types of hematological malignancies (including leukemia, Hodgkin’s lymphoma, Non-Hodgkin’s lymphoma, and multiple myeloma) were defined according to the International Classification of Diseases (ICD), with the diagnostic codes described in Supplementary Table 1 [20]. Leukemia was further subdivided into acute myeloid leukemia (AML) and chronic lymphocytic leukemia (CLL). Participants who were diagnosed with hematological malignancy prior to their enrollment in the UK Biobank were excluded from the analysis, leaving 495,005 individuals in the study. Information regarding date of death was obtained via linkage to death registry records from the NHS. Follow-up for the participants started from the date of enrollment and continued until the diagnosis of hematological malignancy, death, loss to follow-up, or the end of the study (December 31, 2019, taking into account the potential influence from the COVID-19 pandemic), whichever occurred first.

### Statistical analyses

Cox proportional hazards models were used to estimate hazard ratios (HRs) for hematological malignancy according to different early-life exposures, with attainting age as the underlying time scale. The proportional hazards assumption was tested using Schoenfeld residuals. Model 1 was adjusted for age (as time scale), sex, ethnicity (white or others), and place of residence (England, Wales and Scotland). Model 2 further adjusted for early-life exposures simultaneously. Sensitivity analyses were further conducted with stratified analysis by sex and age (< 60 and > 60 years old), and including only White population. For the identified associations between early-life exposures and hematological malignancy, to test whether the associations were mediated by corresponding adulthood factors (such as smoking status, BMI and height), further mediation analyses were performed using the med4way package based on model 2 [21].

### Meta-analysis for all available evidence

A meta-analysis was performed to summarize all available evidence on the associations between early-life exposures and hematological malignancies, incorporating previous prospective cohort studies along with this study. Two investigators (JZ and HY) independently searched for studies indexed in PubMed, Embase, Web of Science and Scopus databases from inception to June 2023. Early-life exposures included birth characteristics (maternal smoking around birth, birth weight, multiple birth status, and being breastfed) and childhood factors (comparative body/height size at age 10, and age at smoking initiation). The detailed search strategy is provided in the Supplementary Table 2. A manual search for additional studies using references from the selected retrieved articles was also performed. After screening titles and abstracts of all citations, short-listed articles were compared by two authors to ensure accuracy and completeness. Discrepancies were resolved by consensus with a third investigator (HH), and the final list of articles used in the meta-analysis is presented in Supplementary Table 3. The study protocol for the meta-analysis was registered in PROSPERO (CRD42023443096). We did additional analysis for birth weight per 1KG increase and smoking initiation < 16 years old in the UK biobank cohort to incorporate our study in the meta-analysis. We performed meta-analyses only when at least two independent studies were available for a given exposure to ensure reliable pooled estimates. Quality assessment of each included study was based on the Newcastle–Ottawa Scale, a nine-star scoring system (Supplementary Table 4). Common or random effects models were used to calculate pooled HRs and 95% CIs for hematologic malignancies, according to the heterogeneity examined by the Cochran Q test and I^2^ statistic.

### Mendelian randomization analysis

Two-sample Mendelian Randomization (MR) analysis was used to assess the potential causal association between early-life exposures with the risk of hematological malignancy. The analysis was performed according to the STROBE-MR guidelines. Genetic instruments for early-life exposures were constructed by identifying single nucleotide polymorphisms (SNPs) associated with them from the IEU open GWAS program with significant *p* < 5 × 10^–8^ [21–23]. The SNPs were selected after excluding those showing linkage disequilibrium (LD threshold: *r*^2^ < 0.001) and their corresponding proportion of variance explained is listed in Supplementary Table 5. As outcomes, GWAS summary statistics for hematological malignancies were obtained from the FinnGen dateset (R9 [24]), including leukemia (AML and CLL), Hodgkin’s lymphoma, Non-Hodgkin’s lymphoma, and multiple myeloma. Two-sample MR analyses were performed using the inverse variance weighting (IVW) method as main models, with alternate MR methods (MR-Egger, and weighted median) used as sensitivity analyses. All statistical tests were two-sided and performed using Stata 15 (Stata Corp, College Station, TX, USA) and R 4.3.1.

## Results

### The associations between early-life exposures and hematological malignancies in the UK biobank

During a median follow-up of 10.8 years, 4,673 patients were diagnosed with hematological malignancies, corresponding to an incidence rate of 0.88/1000 person years. Non-Hodgkin’s lymphoma was the most prevalent type of hematological malignancy, with 2,271 cases, followed by leukemia with 1,377 cases. Table 1 summarizes the key characteristics of the participants, indicating that patients with hematological malignancies were more likely to be older and male, and have obesity.

**Table 1:** Baseline characteristics of the UK Biobank participants overall and in hematological malignancy patients

	**Total**	**Overall**	**Leukemia**	**Hodgkin’s lymphoma**	**Non-Hodgkin’s lymphoma**	**Multiple myeloma**
	**(*N* =,%)**	**(*N* =,%)**	**(*N* =,%)**	**(*N* =,%)**	**(*N* =,%)**	**(*N* =,%)**
Age (mean + sd)	56.98 (8.09)	61.14 (6.81)	61.37 (6.58)	58.90 (7.61)	61. 06 (6.81)	61.48 (6.69)
Sex						
Male	224 917 (45.44)	2 625 (56.17)	832 (60.42)	84 (53.16)	1 221 (53.76)	574 (57.23)
Female	270 088 (54.56)	2 048 (43.83)	545 (39.58)	74 (46.84)	1 050 (46.24)	429 (42.77)
Region						
England	439 808 (88.85)	4 179 (89.43)	1 227 (89.11)	139 (87.97)	2 034 (89.56)	903 (90.03)
Wales	19 926 (4.03)	123 (2.63)	40 (2.90)	4 (2.53)	65 (2.86)	22 (2.19)
Scotland	35 271 (7.13)	371 (7.94)	110 (7.99)	15 (9.49)	172 (7.57)	78 (7.78)
BMI						
< 18.5	5 646 (1.14)	38 (0.81)	10 (0.73)	2 (1.27)	21 (0.92)	6 (0.60)
18.5–24.9	160 187 (32.36)	1 333 (28.53)	373 (27.09)	45 (28.48)	679 (29.90)	265 (26.42)
25–29.9	208 868 (42.20)	2 060 (44.08)	635 (46.11)	58 (36.71)	996 (43.86)	446 (44.47)
≥ 30	120 304 (24.30)	1 242 (26.58)	359 (26.07)	53 (33.54)	575 (25.32)	286 (28.51)
Ethnicity						
White	465 534 (94.57)	4 457 (96.12)	1 334 (97.23)	148 (94.87)	2 175 (96.67)	933 (93.77)
Mixed	7 428 (1.51)	47 (1.01)	13 (0.95)	2 (1.28)	19 (0.84)	13 (1.31)
Asian	11 354 (2.31)	66 (1.42)	11 (0.80)	3 (1.92)	36 (1.60)	17 (1.71)
Black	7 961 (1.62)	67 (1.44)	14 (1.02)	3 (1.92)	20 (0.89)	32 (3.22)
Unknown	2 728	36	5	2	21	8
Physical activity level						
Quintile 1	79 437 (20.04)	755 (20.56)	235 (21.31)	27 (23.48)	356 (20.00)	161 (20.46)
Quintile 2	79 148 (19.96)	695 (18.92)	206 (18.68)	21 (18.26)	337 (18.93)	154 (19.57)
Quintile 3	79 472 (20.05)	712 (19.38)	216 (19.58)	25 (21.74)	331 (18.60)	161 (20.46)
Quintile 4	79 126 (19.96)	765 (20.83)	218 (19.76)	24 (20.87)	397 (22.30)	151 (19.19)
Quintile 5	79 275 (20.00)	746 (20.31)	228 (20.67)	18 (15.65)	359 (22.30)	160 (20.33)
Unknown	98 547	1 000	274	43	491	216
Drinking status						
Never	22 061 (4.47)	215 (4.62)	56 (4.08)	10 (6.37)	93 (4.11)	60 (5.99)
Previous	17 779 (3.60)	177 (3.80)	41 (2.99)	9 (5.73)	90 (3.98)	40 (4.00)
Current	453 537 (91.93)	4 264 (91.58)	1 274 (92.92)	138 (87.90)	2 080 (91.91)	901 (90.01)
Unknown	1 628	17	6	1	8	2

Overall, in model 2 with full covariate adjustment, having a larger body size at age 10 was positively associated with risk of hematological malignancies in adulthood (HR = 1.16, 95% CI = 1.06; 1.27), especially for leukemia (HR = 1.24, 95% CI = 1.05; 1.45) and Non-Hodgkin’s lymphoma (HR = 1.14, 95% CI = 1.00; 1.30) (Tables 2 and 3). These associations were partially mediated by BMI in adulthood (by 35.90% overall, and 56.98% and 23.55% for leukemia and Non-Hodgkin’s lymphoma, respectively, Supplementary Table 6). Similarly, taller participants at age 10 had an increased risk of hematological malignancies (HR = 1.24, 95% CI = 1.14; 1.36), primarily leukemia (HR = 1.34, 95% CI = 1.14; 1.58) and Non-Hodgkin’s lymphoma (HR = 1.29, 95% CI = 1.14; 1.47), with 88.08%, 87.44% and 69.67% of the risk mediated by adulthood height. In addition, an elevated risk of hematological malignancies was also observed among those who started smoking before the age of 14 (HR = 1.17, 95% CI = 1.03; 1.32), as compared to non-smokers, with the strongest effect for leukemia (HR = 1.46, 95% CI = 1.18; 1.81). However, we observed a direct effect of younger age at smoking initiation on leukemia risk without mediation of current smoking status. In a further analysis of leukemia subtypes, the effects of body and height size at age 10 were similar for CLL and AML, while a stronger effect of smoking initiation before 14 was observed for AML (HR = 1.65, 95% CI = 1.07;2.56, Supplementary Table 7). However, maternal smoking around birth, multiple births and being breastfed as a baby were not associated with an increased risk of hematological malignancies, while a birth weight > 4 kg was only associated with a threefold increased risk of Hodgkin’s lymphoma (HR = 3.08, 95% CI = 1.14; 8.35). Model 1 adjusting for age, sex, ethnicity, and place of residence showed similar findings.

**Table 2:** Risk of hematological malignancies in the UK biobank cohort by early-life exposures

	**Hematological malignancies**			
	**Total number**	**NO of events**	**Hazard ratio (95%CI)**	
			Model 1^*^	Model 2^*^
Maternal smoking around birth				
No	301,692	2831	1.00 (REF)	1.00 (REF)
Yes	124,780	1145	1.02 (0.96;1.10)	1.02 (0.95;1.09)
Part of a multiple birth				
No	474,919	4489	1.00 (REF)	1.00 (REF)
Yes	11,064	108	1.05 (0.87;1.28)	1.09 (0.89;1.32)
Birth weight				
< 2.5 kg	27,880	226	1.00 (REF)	1.00 (REF)
2.5-4 kg	207,739	1668	1.04 (0.91;1.20)	1.02 (0.89;1.18)
> 4 kg	37,635	378	1.14 (0.97;1.34)	1.09 (0.92;1.29)
Breastfed as a baby				
No	104,811	771	1.00 (REF)	1.00 (REF)
Yes	273,448	2596	1.01 (0.93;1.10)	1.01 (0.93;1.10)
Comparative body size at age 10				
Thinner	161,312	1448	1.00 (REF)	1.00 (REF)
About average	245,836	2375	**1.07 (1.00;1.14)**	1.06 (0.99;1.14)
Larger	76,964	745	**1.18 (1.08;1.29)**	**1.16 (1.06;1.27)**
Comparative height size at age 10				
Shorter	99,198	812	1.00 (REF)	1.00 (REF)
About average	263,236	2500	**1.13 (1.04;1.22)**	**1.12 (1.03;1.21)**
Taller	122,322	1259	**1.26 (1.15;1.37)**	**1.24 (1.14;1.36)**
Smoking age				
No smoking	269,721	2347	1.00 (REF)	1.00 (REF)
5–14	25,162	275	**1.17 (1.03;1.33)**	**1.17 (1.03;1.32)**
15–18	91,806	982	1.04 (0.96;1.12)	1.03 (0.96;1.11)
> 18	39,467	390	0.98 (0.88;1.09)	0.97 (0.87;1.08)

*CI* confidence interval, *HR* hazard ratio

^*^Model 1 adjusted for age, sex, region of residence and ethnicity. Model 2 further adjusted for maternal smoking, multiple births, birth weight, being breastfed, body size at age 10, height size at age 10, and age at smoking initiation simultaneously. Statistically significant results are bolded

**Table 3:** Risk of the subtypes of hematological malignancies in the UK biobank cohort by early-life exposures

		**Leukemia**			**Hodgkin’s lymphoma**			**Non-Hodgkin’s lymphoma**			**Multiple myeloma**		
	**Total number**	**NO of events**	**Hazard ratio (95%CI)**		**NO of events**	**Hazard ratio (95%CI)**		**NO of events**	**Hazard ratio (95%CI)**		**NO of events**	**Hazard ratio (95%CI)**	
			Model 1^*^	Model 2^*^		Model 1^*^	Model 2^*^		Model 1^*^	Model 2^*^		Model 1^*^	Model 2^*^
Maternal smoking around birth													
No	301,692	813	1.00 (REF)	1.00 (REF)	91	1.00 (REF)	1.00 (REF)	1345	1.00 (REF)	1.00 (REF)	649	1.00 (REF)	1.00 (REF)
Yes	124,780	349	1.07 (0.94;1.21)	1.06 (0.93;1.20)	45	1.21 (0.84;1.73)	1.18 (0.82;1.71)	586	1.09 (0.99;1.21)	1.09 (0.99;1.20)	211	0.86 (0.73;1.00)	0.86 (0.74;1.01)
Part of a multiple birth													
No	474,919	1333	1.00 (REF)	1.00 (REF)	154	1.00 (REF)	1.00 (REF)	2169	1.00 (REF)	1.00 (REF)	966	1.00 (REF)	1.00 (REF)
Yes	11,064	25	0.82 (0.55;1.22)	0.85 (0.57;1.27)	3	0.84 (0.27;2.63)	1.01 (0.32;3.24)	59	1.19 (0.92;1.54)	1.21 (0.93;1.58)	23	1.05 (0.69;1.58)	1.10 (0.72;1.68)
Birth weight													
< 2.5 kg	27,880	62	1.00 (REF)	1.00 (REF)	5	1.00 (REF)	1.00 (REF)	121	1.00 (REF)	1.00 (REF)	44	1.00 (REF)	1.00 (REF)
2.5-4 kg	207,739	496	1.11 (0.85;1.44)	1.05 (0.80;1.37)	65	1.77 (0.71;4.41)	1.83 (0.72;4.62)	807	0.95 (0.78;1.15)	0.94 (0.77;1.15)	348	1.13 (0.82;1.54)	1.13 (0.82;1.55)
> 4 kg	37,635	109	1.14 (0.83;1.56)	1.03 (0.75;1.42)	22	**3.07 (1.16;8.13)**	**3.08 (1.14;8.35)**	177	1.02 (0.81;1.29)	0.99 (0.78;1.26)	78	1.20 (0.83;1.74)	1.19 (0.81;1.74)
Breastfed as a baby													
No	104,811	230	1.00 (REF)	1.00 (REF)	31	1.00 (REF)	1.00 (REF)	365	1.00 (REF)	1.00 (REF)	159	1.00 (REF)	1.00 (REF)
Yes	273,448	770	1.00 (0.86;1.16)	0.99 (0.85;1.15)	89	0.96 (0.64;1.46)	0.95 (0.62;1.44)	1251	1.06 (0.94;1.19)	1.07 (0.95;1.20)	560	0.99 (0.83;1.18)	0.97 (0.81;1.16)
Comparative body size at age 10													
Thinner	161,312	428	1.00 (REF)	1.00 (REF)	48	1.00 (REF)	1.00 (REF)	686	1.00 (REF)	1.00 (REF)	323	1.00 (REF)	1.00 (REF)
About average	245,836	679	1.03 (0.91;1.16)	1.03 (0.91;1.16)	79	1.08 (0.75;1.54)	1.14 (0.79;1.64)	1183	1.12 (1.02;1.23)	1.11 (1.01;1.22)	504	1.02 (0.89;1.17)	1.02 (0.89;1.18)
Larger	76,964	233	**1.27 (1.08;1.49)**	**1.24 (1.05;1.45)**	28	1.28 (0.80;2.04)	1.24 (0.78;1.99)	352	**1.17 (1.02;1.33)**	**1.14 (1.00;1.30)**	159	1.16 (0.96;1.40)	1.15 (0.95;1.40)
Comparative height size at age 10													
Shorter	99,198	230	1.00 (REF)	1.00 (REF)	38	1.00 (REF)	1.00 (REF)	379	1.00 (REF)	1.00 (REF)	197	1.00 (REF)	1.00 (REF)
About average	263,236	729	**1.16 (1.00;1.34)**	1.15 (0.99;1.34)	71	0.69 (0.47;1.03)	**0.67 (0.45;0.99)**	1237	**1.20 (1.07;1.35)**	**1.18 (1.05;1.33)**	525	0.97 (0.83;1.15)	0.96 (0.81;1.13)
Taller	122,322	389	**1.36 (1.16;1.60)**	**1.34 (1.14;1.58)**	46	0.98 (0.64;1.51)	0.90 (0.59;1.40)	607	**1.30 (1.14;1.48)**	**1.29 (1.14;1.47)**	257	1.06 (0.88;1.28)	1.04 (0.86;1.25)
Smoking age													
No smoking	269,721	637	1.00 (REF)	1.00 (REF)	77	1.00 (REF)	1.00 (REF)	1169	1.00 (REF)	1.00 (REF)	522	1.00 (REF)	1.00 (REF)
5–14	25,162	99	**1.47 (1.19;1.82)**	**1.46 (1.18;1.81)**	13	1.75 (0.97;3.18)	1.69 (0.93;3.07)	137	**1.20 (1.00;1.43)**	1.18 (0.99;1.42)	40	0.77 (0.56;1.07)	0.78 (0.57;1.08)
15–18	91,806	310	**1.16 (1.01;1.33)**	**1.15 (1.00;1.32)**	35	1.24 (0.83;1.86)	1.22 (0.82;1.84)	475	1.02 (0.92;1.14)	1.01 (0.91;1.13)	197	0.93 (0.79;1.10)	0.93 (0.79;1.10)
> 18	39,467	114	1.04 (0.85;1.27)	1.03 (0.84;1.26)	13	1.09 (0.61;1.97)	1.07 (0.59;1.94)	186	0.95 (0.81;1.11)	0.94 (0.81;1.10)	90	0.99 (0.79;1.24)	0.99 (0.79;1.24)

*CI* confidence interval, *HR* hazard ratio

^*^Model 1 adjusted for age, sex, region of residence and ethnicity. Model 2 further adjusted for maternal smoking, multiple births, birth weight, being breastfed, body size at age 10, height size at age 10, and age at smoking initiation simultaneously. Statistically significant results are bolded

In the sensitivity analysis stratified by sex and age, there were no significant differences in the effects of these factors (Supplementary Tables 8–11), and the *p*-values for the interaction tests were > 0.05. Including only the White population yielded similar results.

### Meta-analysis of early-life exposures and hematological malignancies

Out of an initial identification of 10,532 records, only 7 cohort studies met our selection criteria, covering a population of 1,999,102 individuals and 6,383 cases of hematological malignancies. Study selection and individual characteristics are displayed in Supplementary Fig. 1 and Supplementary Table 3. Our meta-analysis examined early-life exposures including birth characteristics (maternal smoking around birth, birth weight, and being breastfed) and childhood factors (comparative body size at age 10 and age at smoking initiation).

**Fig. 1:**
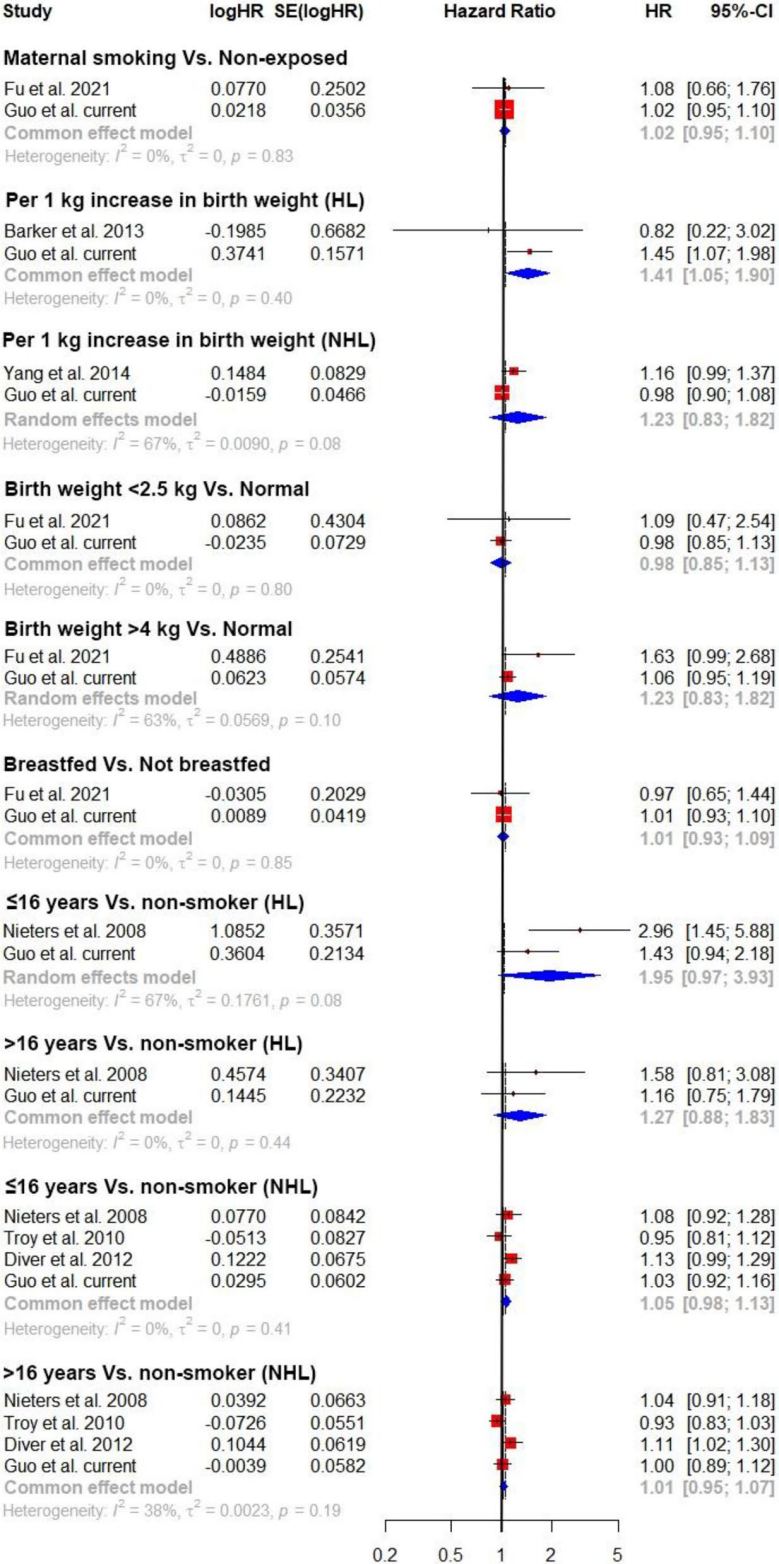
Meta-analysis of available studies on early-life exposures and risk of hematological malignancies. HL: Hodgkin’s lymphoma; NHL: Non- Hodgkin’s lymphoma. The outcome was hematological malignancy overall if not noted in the brackets

After conducting meta-analysis in combination with our study, maternal smoking around birth and being breastfed as a baby were still not associated with the risk of hematological malignancies (Fig. 1). Increase of birth weight by 1 kg was positively associated with risk of Hodgkin’s lymphoma (HR = 1.41, 95% CI = 1.05; 1.90), although the association was dominated by our study. A twofold increased risk of Hodgkin’s lymphoma was observed among individuals who started smoking younger than 16 years old. However, the association was not statistically significant in the random effect model (HR = 1.95, 95% CI = 0.97; 3.93). Multiple birth and height at age 10 were not included in the meta-analysis as they had not been studied previously.

### MR analysis of early-life exposures and hematological malignancies

**Fig. 2:**
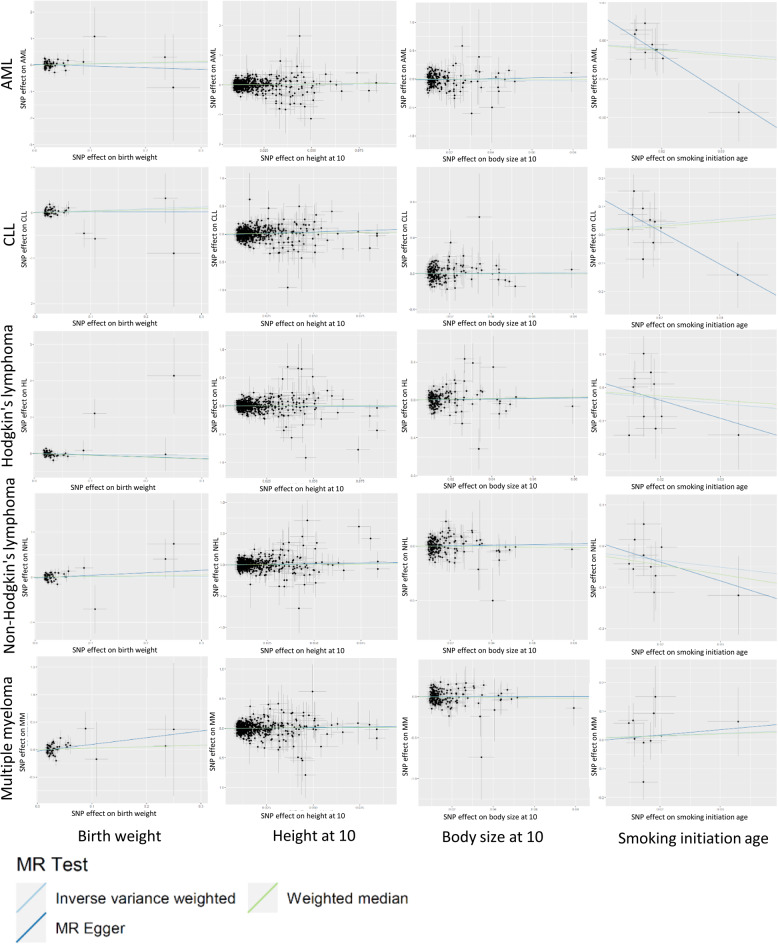
Scatter plot for the effect of early-life exposures and risk of hematological malignancy in the Mendelian randomization analysis. All the SNPs are plotted according to their effects on hematological malignancy (y axis, including AML, CLL, Hodgkin’s lymphoma, Non- Hodgkin’s lymphoma and multiple myeloma) and early-life exposures (x axis, including birth weight, height at age 10, body size at age 10 and age at smoking initiation), with their confidence intervals. Mendelian randomization analyses were performed using the inverse variance weighting method as main models, with alternate MR methods (MR-Egger and weighted median) used as sensitivity analyses

To assess the potential causal association between early-life exposures and the risk of hematological malignancy, 925 SNPs were selected as genetic instruments. The number of SNPs used as genetic instruments for each early-life exposure was: 63 for birth weight, 648 for height at age 10, 10 for age at smoking initiation, and 204 for body size at age 10. These SNPs explained 0.1%–6.1% of the variance in early-life exposures, with corresponding F-statistic ranged from 29–87 (Supplementary Table 5), indicating no evidence of weak instrument bias. The two-sample Mendelian Randomization analysis found a notable association between height at age 10 and CLL (β = 0.36, 95%CI: 0.01; 0.71, *p* = 0.04) using both the IVW method and Egger regression method (Fig. 2, Supplementary Table 14). Age at smoking initiation was inversely associated with AML in the Egger regression model (β = −24.57, 95%CI: −44.98; −4.15, *p* = 0.046), but the association was not statistically significant in the IVW model. Birth weight and body size at age 10 had no causal effect on the risk of hematological malignancies.

## Discussions

We observed a 16% increased risk of hematological malignancies in individuals with a larger body size at age 10. While previous studies have mainly focused on adult BMI and the risk of several hematological malignancies [25–27], only two studies have reported the association between childhood BMI and Non-Hodgkin’s lymphoma [27, 28], supporting our findings. Furthermore, we also found an association between childhood body size and leukemia. Individuals with higher BMI are known to be in a pro-inflammatory state with altered levels of circulating cytokines, growth factors, and adipokines, which are involved in the pathways related to carcinogenesis of Non-Hodgkin’s lymphoma and leukemia, such as B-cell proliferation, energy homeostasis, or immunomodulation [29–33]. Our study suggested that this effect started early in childhood, and partially mediated by adulthood BMI.

Apart from body size, height at age 10 was also associated with the risk of hematological malignancies, especially for leukemia. These findings are aligned with previous studies [13, 28, 34], and our MR analysis further supported the potential causal association for CLL. Plausible mechanisms behind the effect of height on the risk of hematological malignancies include an increase in cell numbers and cell divisions, as well as elevated levels of growth hormones like IGF-1, which play a significant role in childhood growth and are also associated with cancer development in adulthood [35, 36].

Smoking has been linked to a higher risk of leukemia, with previous research demonstrating that the duration of smoking is correlated with an increased risk [37]. In our study, we found that younger age at smoking initiation might also increase the risk of leukemia, which was independent of the adulthood smoking status, further suggesting the importance of early life exposures on leukemia risk. Chemicals found in tobacco smoke, such as benzene, formaldehyde, and other carcinogens, are associated with leukemogenesis [38]. Affected by cigarette smoke extract, erythrocyte and granulocyte colony-forming units in bone marrow up-regulate toll-like receptor expression and promote the production of inflammatory mediators, which contribute to myelofibrosis and the pathogenesis of leukemia [39]. The potential causal association supported by MR analysis emphasized the importance of cigarette smoking in the development of AML and suggested that tobacco control interventions targeting adolescents might be beneficial in preventing AML later in life.

### Strength and limitations

Our study benefited from a large-scale community-based cohort in the UK, which provided abundant information on hematological malignancy and life-style exposures. We further used meta-analysis and MR analysis to validate the associations.

There are also limitations to our study. First, the collection of early-life exposures heavily relied on questionnaires, which introduces the possibility of recall biases. As this recall bias was not associated with outcomes, this may only lead to misclassification of exposures and attenuate the observed estimates for the associations [40]. Second, healthy volunteer bias among participants in the UK Biobank cannot be avoided. However, the associations found in our study can still be broadly generalized to the population of European ancestry. Third, while restricting our meta-analysis to cohort studies enhances internal validity, it may limit generalizability and reduce statistical power by excluding case–control studies considering that hematological cancers are rare in the population.

## Conclusion

In conclusion, our findings indicate that having a larger body size at age 10, taller height size at age 10 and smoking initiation < 14 years old were associated with an increased risk of hematological malignancies in adulthood, particularly for leukemia and NHL. Additionally, the relationship between birth weight and Hodgkin’s lymphoma remains unclear and requires further investigation.

## Supplementary Information

Supplementary Material 1.

## Data Availability

Data from the UK Biobank (http://www.ukbiobank.ac.uk/) are available to researchers upon application. This research was conducted using the UK Biobank Resource under Application 61083.

## Abbreviations

AML: Acute myeloid leukemia
CI: Confidence interval
CLL: Chronic lymphocytic leukemia
HR: Hazard Ratio
HL: Hodgkin’s lymphoma
IVW: Inverse variance weighting
LD: Linkage disequilibrium
MR: Mendelian Randomization
MM: Multiple myeloma
NHL: Non- Hodgkin’s lymphoma
SNPs: Single nucleotide polymorphisms
UKB: The UK Biobank
