# Supplementary material for: Early-life exposures and risk of hematological malignancies in adulthood: a cohort study, meta-analysis and Mendelian randomization analysis
Source: BMC Cancer. 2025 Oct 8;25:1532. doi: 10.1186/s12885-025-14780-y (PMC12506295; doi:10.1186/s12885-025-14780-y)
Supplement: Supplementary file 1 — Supplementary Material 1. [file 12885_2025_14780_MOESM1_ESM.docx]

**Supplementary table 1**. ICD coding for hematological malignancies

|  | |  |
| --- | --- | --- |
|  | **ICD9** | **ICD10** |
| Non-Hodgkin's lymphoma | 200,202 | C82, C83, C84,C85, C86 |
| Hodgkin lymphoma | 201 | C81 |
| multiple myeloma | 203 | C88,C90 |
| Leukemia | 204,205,206,207,208 | C91,C92,C93,C94,C95 |
| acute myelocytic leukemia (AML) | 2050 | C920 |
| chronic lymphocytic leukemia (CLL) | 2041 | C911 |

**Supplementary table 2**. Search strategy for systematic review^*^

| Step | PubMed | Web of Science | Embase | Scopus |
| --- | --- | --- | --- | --- |
| #1 | Hematologic neoplasms[MeSH] OR leukemia[MeSH] OR lymphoma[MeSH] OR multiple myeloma[MeSH] OR hematologic neoplasms[tiab] OR blood cancer[tiab] OR leukemia[tiab] OR leukaemia[tiab] OR lymphoma[tiab] OR Hodgkin disease[tiab] OR multiple myeloma[tiab] | TS=(hematologic neoplasms OR leukemia OR lymphoma OR multiple myeloma) AND TI=(hematologic neoplasms OR blood cancer OR leukemia OR leukaemia OR lymphoma OR Hodgkin disease OR multiple myeloma) OR AB=(hematologic neoplasms OR blood cancer OR leukemia OR leukaemia OR lymphoma OR Hodgkin disease OR multiple myeloma) | ('hematologic neoplasms'/exp OR 'leukemia'/exp OR 'lymphoma'/exp OR 'multiple myeloma'/exp) AND ('hematologic neoplasms':ab,ti OR 'blood cancer':ab,ti OR 'leukemia':ab,ti OR 'leukaemia':ab,ti OR 'lymphoma':ab,ti OR 'Hodgkin disease':ab,ti OR 'multiple myeloma':ab,ti)) | KEY("hematologic neoplasms" OR "leukemia" OR "lymphoma" OR "multiple myeloma") OR TITLE((hematologic neoplasms) OR (blood cancer) OR leukemia OR leukaemia OR lymphoma OR (Hodgkin disease) OR (multiple myeloma)) OR ABS((hematologic neoplasms) OR (blood cancer) OR leukemia OR leukaemia OR lymphoma OR (Hodgkin disease) OR (multiple myeloma)) |
| #2 | maternal exposure[MeSH] OR multiple birth offspring[MeSH] OR birth weight[MeSH] breast feeding[MeSH] OR milk, human[MeSH] OR bottle feeding[MeSH] OR growth[MeSH] OR smoking[MeSH] OR early life exposure[tiab] OR multiple birth[tiab] OR multiple pregnancy[tiab] OR multiple embryos[tiab] OR polyembryony[tiab] OR single birth[tiab] OR single embryo[tiab] OR breastfeed[tiab] OR breast feed[tiab] OR breastfeeding[tiab] OR breast feeding[tiab] OR breastfed[tiab] OR breast fed[tiab] OR human milk[tiab] OR breast milk[tiab] OR infant diet[tiab] OR infant food[tiab] OR infant feed[tiab] OR infant feeding[tiab] OR infant nutrition[tiab] OR early nutrition[tiab] OR bottlefeed[tiab] OR bottle feed[tiab] OR bottlefeeding[tiab] OR bottle feeding[tiab] OR bottlefed[tiab] OR bottle fed[tiab] OR body size[tiab] OR body weight[tiab] OR weight size[tiab] OR height [tiab] OR body mass index[tiab] OR BMI[tiab] OR obesity[tiab] OR adiposity[tiab] OR thinness[tiab] OR underweight[tiab] OR overweight[tiab] OR smoking[tiab] | TS=(maternal exposure OR multiple birth OR multiple pregnancy OR birth weight OR breast feed OR breastfeed OR milk, human OR bottle feed OR bottlefeed OR growth OR body size OR body weight OR height OR body mass index OR BMI OR smoking) AND  (TI=(early life exposure OR multiple birth OR multiple pregnancy OR multiple embryos OR polyembryony OR single birth OR single embryo OR breast feed OR breastfeed OR human milk OR breast milk OR infant diet OR infant food OR infant feed OR infant feeding OR infant nutrition OR early nutrition OR bottle feed OR bottlefeed OR body size OR body weight OR weight size OR height OR body mass index OR BMI OR obesity OR adiposity OR thinness OR underweight OR overweight OR smoking) OR AB=(early life exposure OR multiple birth OR multiple pregnancy OR multiple embryos OR polyembryony OR single birth OR single embryo OR breast feed OR breastfeed OR human milk OR breast milk OR infant diet OR infant food OR infant feed OR infant feeding OR infant nutrition OR early nutrition OR bottle feed OR bottlefeed OR body size OR body weight OR weight size OR height OR body mass index OR BMI OR obesity OR adiposity OR thinness OR underweight OR overweight OR smoking)) | ('maternal exposure'/exp OR 'multiple birth'/exp OR 'multiple pregnancy'/exp OR 'birth weight'/exp OR 'breast feeding'/exp OR 'breast milk'/exp OR 'bottle feeding'/exp OR 'growth'/exp OR 'body size'/exp OR 'body weight'/exp OR 'height'/exp OR 'body mass index'/exp OR 'BMI'/exp OR smoking/exp) AND ('early life exposure':ab,ti OR 'multiple birth':ab,ti OR 'multiple pregnancy':ab,ti OR 'multiple embryos':ab,ti OR 'polyembryony':ab,ti OR 'single birth':ab,ti OR 'single embryo':ab,ti OR 'breastfeed':ab,ti OR 'breast feed':ab,ti OR 'breastfeeding':ab,ti OR 'breast feeding':ab,ti OR 'breastfed':ab,ti OR 'breast fed':ab,ti OR 'human milk':ab,ti OR 'breast milk':ab,ti OR 'infant diet':ab,ti OR 'infant food':ab,ti OR 'infant feed':ab,ti OR 'infant feeding':ab,ti OR 'infant nutrition':ab,ti OR 'early nutrition':ab,ti OR 'bottlefeed':ab,ti OR 'bottle feed':ab,ti OR 'bottlefeeding':ab,ti OR 'bottle feeding':ab,ti OR 'bottlefed':ab,ti OR 'bottle fed':ab,ti OR 'body size':ab,ti OR 'body weight':ab,ti OR 'weight size':ab,ti OR 'height':ab,ti OR 'body mass index':ab,ti OR 'BMI':ab,ti OR 'obesity':ab,ti OR 'adiposity':ab,ti OR 'thinness':ab,ti OR 'underweight':ab,ti OR 'overweight':ab,ti OR 'smoking':ab,ti | KEY("maternal exposure" OR "multiple birth" OR "multiple pregnancy" OR "birth weight" OR "breast feeding" OR "bottle feeding" OR "milk, human" OR "breast milk" OR "infant feeding" OR "infant nutrition" OR growth OR "body size" OR "body weight" OR height OR "body mass index" OR BMI OR "smoking") OR (TITLE((early life exposure) OR (multiple birth) OR (multiple pregnancy) OR (multiple embryos) OR polyembryony OR (single birth) OR (single embryo) OR breastfeed OR (breast feed) OR breastfeeding OR (breast feeding) OR breastfed OR (breast fed) OR (human milk) OR (breast milk) OR (infant diet) OR (infant food) OR (infant feed) OR (infant feeding) OR (infant nutrition) OR (early nutrition) OR bottlefeed OR (bottle feed) OR bottlefeeding OR (bottle feeding) OR bottlefed OR (bottle fed)OR (body size) OR (body weight) OR (weight size) OR height OR (body mass index) OR BMI OR obesity OR adiposity OR thinness OR underweight OR overweight OR smoking)) OR ABS((early life exposure) OR (multiple birth) OR (multiple pregnancy) OR (multiple embryos) OR polyembryony OR (single birth) OR (single embryo) OR breastfeed OR (breast feed) OR breastfeeding OR (breast feeding) OR breastfed OR (breast fed) OR (human milk) OR (breast milk) OR (infant diet) OR (infant food) OR (infant feed) OR (infant feeding) OR (infant nutrition) OR (early nutrition) OR bottlefeed OR (bottle feed) OR bottlefeeding OR (bottle feeding) OR bottlefed OR (bottle fed) OR (body size) OR (body weight) OR (weight size) OR height OR (body mass index) OR BMI OR obesity OR adiposity OR thinness OR underweight OR overweight OR smoking))) |
| #3 | cohort studies[MeSH] OR cohort[tiab] OR prospective[tiab] OR "follow up"[tiab] OR follow-up[tiab] OR longitudinal[tiab] | TS=(cohort study) OR TI=(cohort OR prospective OR follow up OR longitudinal) OR AB=(cohort OR prospective OR "follow up" OR longitudinal) | 'cohort study'/exp OR 'follow up study':ab,ti OR 'prospective study':ab,ti OR 'longitudinal study':ab,ti OR 'cohort':ab,ti OR 'prospective':ab,ti OR 'follow up':ab,ti OR 'follow-up':ab,ti OR 'longitudinal':ab,ti | KEY("cohort study" OR "follow up study" OR "prospective study" OR "longitudinal study") OR TITLE(cohort OR prospective OR "follow up" OR longitudinal) OR ABS(cohort OR prospective OR "follow up" OR longitudinal) |
| #4 | humans[Mesh] | TS=(rat OR rats OR animal OR animals OR mice OR "in vivo" OR mouse OR rabbit OR rabbits OR murine) OR TI=(rat OR rats OR animal OR animals OR mice OR "in vivo" OR mouse OR rabbit OR rabbits OR murine) OR AB=(rat OR rats OR animal OR animals OR mice OR "in vivo" OR mouse OR rabbit OR rabbits OR murine) | 'human'/exp | INDEXTERMS (humans OR human) |
| #5 | #1 AND #2 AND #3 AND #4 | (#1 AND #2 AND #3) NOT #4 | #1 AND #2 AND #3 AND #4 | #1 AND #2 AND #3 AND #4 |
| Final | 3139 | 3094 | 2720 | 1575 |

^*^ Search date: 14 June 2023.

**Supplementary table 3.** Study characteristics in the meta-analysis for hematological malignancies

| Study | Country | Sample size | Age at baseline | Enrollment period | Years of follow-up | Exposure categories | Outcome | Adjustments |
| --- | --- | --- | --- | --- | --- | --- | --- | --- |
| Nieters et al. 2008 | 10 European countries | 478590 | >18 years | 1992-2000 | Median:  8.5 years | Age of smoking initiation  Never smoked (Ref);  <=16 years;  >16 years | Hodgkin’s lymphoma /non-Hodgkin’s lymphoma | Educational level, stratified by center, sex, and age at recruitment |
| Troy et al. 2010 | the United States | 142982 | 55-74 years | 1993-2001 | Median:  8.8 years | Age of smoking initiation  Never smoked (Ref);  <=16 years;  >16 years | Non-Hodgkin's lymphoma | Age, sex, race/ethnicity, and education |
| Diver et al. 2012 | the United States | 152958 | 55-74 years | 1992 | 15 years | Never smoked (Ref);  <=16 years;  >16 years | Non-Hodgkin's lymphoma | Age, gender, family history of hematopoietic cancer, education, METS, BMI, height, and alcohol use |
| Barker et al. 2013 | Finland | 20431 | At birth | 1924-1944 | up to 2006 | Per 1 kg increase in birth weight | Hodgkin’s lymphoma | Year of birth, sex, and length of gestation |
| Yang et al. 2014 | England and Scotland | 453023  (female) | 50-64 years | 1996-2001 | Average: 9.2 years | Per 1 kg increase in birth weight | Non-Hodgkin's lymphoma | Age, year of birth, region, and adult height and smoking |
| Yang et al. 2016 | England and Scotland | 745273 (female) | 50-64 years | 1996-2001 | Average: 10.9 years | Comparative body size at age 10  Average/thinner (Ref)；  larger | Hodgkin’s lymphoma | Age, year of birth, region, socioeconomic status, height, smoking amount and status, alcohol consumption, exercise, and recent BMI |
| Fu et al. 2021 | the United States | 5845 (male) | 40-75 years | 1986 | 20 years | (1) Birth weight <2.5kg; 2.5-3.9kg (Ref); >4kg (2) Breastfed as a baby No (Ref); yes (3) Maternal smoking No (Ref); yes | Blood cancers | Age, time period, race, family history of cancer, maternal education, and paternal education |
| Guo et al. current | United Kingdom | 501806 | 40-70 | 2006-2010 | Median: 10.8 years | (1) Age of smoking initiation  Never smoked (Ref);<=16 years;  >16 years  (2) Birth weight;<2.5kg;2.5-3.9kg (Ref);>4kg; per 1 kg  (3) Breastfed as a baby ;No (Ref); yes  (4) Maternal smoking;No (Ref); yes | Hodgkin’s lymphoma/  non-Hodgkin’s lymphoma/  blood cancers | Age, sex, ethnicity, and place of residence, aternal smoking, multiple births, birth weight, being breastfed, body size at age 10, height size at age 10, and age at smoking initiation |

**Supplementary Table 4**. Evaluation of risk of bias according to the Newcastle-Ottawa Scale

| Study | Newcastle-Ottawa Scale^*^ | | | | | | | | | | |
| --- | --- | --- | --- | --- | --- | --- | --- | --- | --- | --- | --- |
|  | **Selection** | | | |  | **Comparability** |  | **Outcome** | | | **Total** |
|  | **Representativeness of the exposed cohort** | **Selection of the non-exposed cohort** | **Ascertainment of exposure** | **Demonstration that outcome of interest was not present at start of study** |  | **Comparability of cohorts on the basis of the design or analysis** |  | **Assessment of outcome** | **Was follow-up long enough for outcomes to occur** | **Adequacy of follow up of cohorts** |  |
| Nieters et al. 2008 | ★ | ★ | ★ | ★ |  | ★★ |  | ★ | ★ | / | 8 |
| Troy et al. 2010 | ★ | ★ | / | ★ |  | ★★ |  | ★ | ★ | / | 7 |
| Diver et al. 2012 | ★ | ★ | / | ★ |  | ★★ |  | ★ | ★ | ★ | 8 |
| Barker et al. 2013 | ★ | ★ | ★ | ★ |  | ★★ |  | ★ | ★ | / | 8 |
| Yang et al. 2014 | ★ | ★ | / | ★ |  | ★★ |  | ★ | ★ | / | 7 |
| Yang et al. 2016 | ★ | ★ | / | ★ |  | ★★ |  | ★ | ★ | / | 7 |
| Fu et al. 2021 | / | ★ | ★ | ★ |  | ★★ |  | ★ | ★ | ★ | 8 |
| Guo et al. current | ★ | ★ | / | ★ |  | ★★ |  | ★ | ★ | ★ | 8 |

^*^ Star (★) represents 1 point awarded. According to Newcastle-Ottawa Scale, a study can be awarded a maximum of one star for each numbered item within the Selection and Outcome categories. A maximum of two stars can be given for Comparability.

**Supplementary Table 5.** SNPs used for Mendelian Randomization analysis

| SNP | chr | BP | EA | BETA | SE | *P* | N | EAF | trait | R2 | F | |
| --- | --- | --- | --- | --- | --- | --- | --- | --- | --- | --- | --- | --- |
| rs12401656 | 1 | 43456767 | G | 0.029 | 0.006 | 1.60E-06 | 292712 | 0.865 | birth_weight |  |  | |
| rs80278614 | 1 | 119412317 | A | 0.052 | 0.009 | 4.00E-08 | 292074 | 0.054 | birth_weight |  |  | |
| rs61830764 | 1 | 212289976 | A | 0.018 | 0.004 | 3.00E-05 | 291445 | 0.377 | birth_weight |  |  | |
| rs2551347 | 2 | 23912401 | T | 0.029 | 0.005 | 2.20E-09 | 292714 | 0.749 | birth_weight |  |  | |
| rs754868 | 2 | 43185532 | G | 0.019 | 0.004 | 4.70E-06 | 298139 | 0.419 | birth_weight |  |  | |
| rs4953353 | 2 | 46567276 | G | 0.019 | 0.004 | 6.50E-06 | 292721 | 0.632 | birth_weight |  |  | |
| rs56188432 | 2 | 158406865 | G | 0.25 | 0.048 | 2.64E-07 | 217397 | 0.002 | birth_weight |  |  | |
| rs10181515 | 2 | 227019461 | T | 0.021 | 0.005 | 1.30E-05 | 298138 | 0.225 | birth_weight |  |  | |
| rs10935733 | 3 | 148622968 | T | 0.021 | 0.004 | 5.00E-07 | 292713 | 0.399 | birth_weight |  |  | |
| rs1482852 | 3 | 156798294 | A | 0.054 | 0.004 | 7.60E-39 | 298130 | 0.599 | birth_weight |  |  | |
| rs11711420 | 3 | 183349010 | T | 0.022 | 0.005 | 2.70E-06 | 292710 | 0.747 | birth_weight |  |  | |
| rs4144829 | 4 | 17903654 | C | 0.032 | 0.005 | 1.10E-11 | 292713 | 0.267 | birth_weight |  |  | |
| rs116807401 | 4 | 135121721 | C | 0.088 | 0.016 | 7.00E-08 | 265314 | 0.018 | birth_weight |  |  | |
| rs351930 | 5 | 52003397 | T | 0.02 | 0.005 | 8.10E-05 | 292714 | 0.801 | birth_weight |  |  | |
| rs1547669 | 6 | 33775641 | G | 0.018 | 0.004 | 9.30E-06 | 289000 | 0.497 | birth_weight |  |  | |
| rs6925689 | 6 | 126865884 | T | 0.018 | 0.004 | 2.10E-05 | 292716 | 0.494 | birth_weight |  |  | |
| rs6930558 | 6 | 141878920 | T | 0.022 | 0.005 | 3.30E-06 | 292714 | 0.747 | birth_weight |  |  | |
| rs10872678 | 6 | 152039964 | T | 0.028 | 0.005 | 8.20E-10 | 298136 | 0.724 | birth_weight |  |  | |
| rs7772579 | 6 | 152042502 | A | 0.027 | 0.005 | 5.80E-09 | 292718 | 0.721 | birth_weight |  |  | |
| rs138715366 | 7 | 44246271 | C | 0.235 | 0.022 | 1.40E-25 | 284324 | 0.991 | birth_weight |  |  | |
| rs10265057 | 7 | 47275737 | G | 0.036 | 0.007 | 4.60E-07 | 292446 | 0.092 | birth_weight |  |  | |
| rs112139215 | 7 | 73034559 | A | 0.056 | 0.008 | 1.20E-11 | 295398 | 0.068 | birth_weight |  |  | |
| rs2282978 | 7 | 92264410 | C | 0.021 | 0.004 | 1.60E-06 | 298140 | 0.326 | birth_weight |  |  | |
| rs732563 | 8 | 23345526 | C | 0.019 | 0.004 | 6.10E-06 | 292723 | 0.504 | birth_weight |  |  | |
| rs34036147 | 8 | 38366249 | T | 0.019 | 0.004 | 1.60E-05 | 292711 | 0.688 | birth_weight |  |  | |
| rs13266210 | 8 | 41533514 | A | 0.03 | 0.005 | 3.10E-09 | 292718 | 0.786 | birth_weight |  |  | |
| rs72656010 | 8 | 57122215 | T | 0.026 | 0.006 | 1.60E-05 | 292713 | 0.868 | birth_weight |  |  | |
| rs7819593 | 8 | 106115172 | C | 0.023 | 0.005 | 2.10E-06 | 292718 | 0.243 | birth_weight |  |  | |
| rs9657468 | 8 | 142362391 | G | 0.018 | 0.004 | 3.60E-05 | 286868 | 0.334 | birth_weight |  |  | |
| rs28457693 | 9 | 98217348 | G | 0.04 | 0.007 | 1.70E-09 | 288037 | 0.109 | birth_weight |  |  | |
| rs1323438 | 9 | 119115531 | C | 0.02 | 0.005 | 1.30E-05 | 292712 | 0.718 | birth_weight |  |  | |
| rs3933326 | 9 | 123633948 | G | 0.023 | 0.004 | 2.20E-07 | 292715 | 0.676 | birth_weight |  |  | |
| rs28505901 | 9 | 139241030 | A | 0.024 | 0.005 | 4.20E-07 | 286903 | 0.249 | birth_weight |  |  | |
| rs7076938 | 10 | 115789375 | T | 0.029 | 0.005 | 2.90E-10 | 298136 | 0.735 | birth_weight |  |  | |
| rs11042596 | 11 | 2118860 | T | 0.027 | 0.004 | 1.60E-09 | 292715 | 0.336 | birth_weight |  |  | |
| rs234864 | 11 | 2857297 | A | 0.017 | 0.004 | 4.90E-05 | 296865 | 0.547 | birth_weight |  |  | |
| rs4444073 | 11 | 10331664 | A | 0.023 | 0.004 | 2.20E-08 | 298137 | 0.520 | birth_weight |  |  | |
| rs11055030 | 12 | 12878349 | G | 0.022 | 0.005 | 1.00E-06 | 292715 | 0.718 | birth_weight |  |  | |
| rs8756 | 12 | 66359752 | C | 0.037 | 0.004 | 1.70E-19 | 298139 | 0.487 | birth_weight |  |  | |
| rs7968682 | 12 | 66371880 | G | 0.037 | 0.004 | 4.90E-20 | 298092 | 0.486 | birth_weight |  |  | |
| rs1480470 | 12 | 66412130 | G | 0.028 | 0.004 | 1.10E-10 | 292712 | 0.631 | birth_weight |  |  | |
| rs72681869 | 14 | 50655357 | C | 0.108 | 0.021 | 2.70E-07 | 259445 | 0.011 | birth_weight |  |  | |
| rs6575803 | 14 | 101257755 | C | 0.034 | 0.007 | 9.90E-07 | 284076 | 0.895 | birth_weight |  |  | |
| rs7402983 | 15 | 99193276 | A | 0.027 | 0.004 | 4.60E-10 | 292717 | 0.405 | birth_weight |  |  | |
| rs40434 | 16 | 55699525 | G | 0.017 | 0.004 | 4.80E-05 | 292714 | 0.391 | birth_weight |  |  | |
| rs222857 | 17 | 7164563 | T | 0.026 | 0.004 | 5.80E-10 | 298132 | 0.575 | birth_weight |  |  | |
| rs4511593 | 17 | 7455536 | T | 0.019 | 0.004 | 7.40E-06 | 292717 | 0.650 | birth_weight |  |  | |
| rs9909342 | 17 | 25652275 | A | 0.019 | 0.004 | 6.70E-06 | 292713 | 0.381 | birth_weight |  |  | |
| rs11867479 | 17 | 68090207 | T | 0.018 | 0.004 | 2.20E-05 | 298138 | 0.353 | birth_weight |  |  | |
| rs10221267 | 17 | 68464662 | T | 0.018 | 0.004 | 1.90E-05 | 296641 | 0.512 | birth_weight |  |  | |
| rs73354194 | 17 | 79905947 | C | 0.06 | 0.014 | 1.70E-05 | 268519 | 0.025 | birth_weight |  |  | |
| rs8106042 | 19 | 7161849 | G | 0.023 | 0.005 | 6.60E-07 | 291451 | 0.281 | birth_weight |  |  | |
| rs41355649 | 19 | 33790556 | G | 0.042 | 0.008 | 4.50E-07 | 291155 | 0.934 | birth_weight |  |  | |
| rs1129156 | 19 | 40719076 | T | 0.022 | 0.005 | 1.90E-06 | 292719 | 0.268 | birth_weight |  |  | |
| rs147957154 | 19 | 43431040 | T | 0.026 | 0.006 | 2.40E-05 | 269001 | 0.132 | birth_weight |  |  | |
| rs11698914 | 20 | 31327144 | C | 0.029 | 0.005 | 2.80E-09 | 292713 | 0.233 | birth_weight |  |  | |
| rs1012167 | 20 | 39159119 | C | 0.024 | 0.004 | 1.90E-08 | 292373 | 0.401 | birth_weight |  |  | |
| rs753381 | 20 | 39797465 | T | 0.018 | 0.004 | 9.10E-06 | 297797 | 0.451 | birth_weight |  |  | |
| rs6026449 | 20 | 57272617 | C | 0.018 | 0.004 | 3.20E-05 | 292375 | 0.627 | birth_weight |  |  | |
| rs73143584 | 20 | 62445702 | A | 0.031 | 0.007 | 3.30E-06 | 286584 | 0.110 | birth_weight |  |  | |
| rs134594 | 22 | 29468456 | C | 0.022 | 0.004 | 6.00E-07 | 290627 | 0.351 | birth_weight |  |  | |
| rs41311445 | 22 | 42070374 | A | 0.034 | 0.007 | 1.30E-06 | 289016 | 0.903 | birth_weight |  |  | |
| rs7285579 | 22 | 46441980 | C | 0.018 | 0.005 | 0.00011 | 290177 | 0.698 | birth_weight | 0.017 | 86.88 |  |
| rs2353983 | 1 | 146813137 | T | 0.010213 | 0.001714 | 2.57E-09 | 332021 | 0.626363 | height_10 |  |  | |
| rs12116935 | 1 | 36789546 | G | -0.01036 | 0.0017 | 1.10E-09 | 332021 | 0.387314 | height_10 |  |  | |
| rs9435731 | 1 | 17306029 | A | 0.018552 | 0.001659 | 4.84E-29 | 332021 | 0.520523 | height_10 |  |  | |
| rs6702280 | 1 | 10280774 | A | 0.015945 | 0.002489 | 1.50E-10 | 332021 | 0.127079 | height_10 |  |  | |
| rs7534091 | 1 | 118864616 | G | -0.02702 | 0.001889 | 2.12E-46 | 332021 | 0.260216 | height_10 |  |  | |
| rs2273368 | 1 | 113063771 | T | -0.01326 | 0.002083 | 1.96E-10 | 332021 | 0.197972 | height_10 |  |  | |
| rs7860 | 1 | 171766696 | G | -0.01395 | 0.001872 | 9.40E-14 | 332021 | 0.268279 | height_10 |  |  | |
| rs10911420 | 1 | 183677511 | T | -0.00934 | 0.001683 | 2.89E-08 | 332021 | 0.578989 | height_10 |  |  | |
| rs559008623 | 1 | 218706189 | C | 0.036454 | 0.006681 | 4.86E-08 | 332021 | 0.0159995 | height_10 |  |  | |
| rs61775433 | 1 | 26416449 | G | 0.013995 | 0.002174 | 1.21E-10 | 332021 | 0.176706 | height_10 |  |  | |
| rs4266877 | 1 | 228107031 | A | -0.01147 | 0.002096 | 4.36E-08 | 332021 | 0.194937 | height_10 |  |  | |
| rs271735 | 1 | 234662348 | A | -0.01176 | 0.001731 | 1.10E-11 | 332021 | 0.360578 | height_10 |  |  | |
| rs57668887 | 1 | 172327399 | C | 0.013256 | 0.001762 | 5.45E-14 | 332021 | 0.33176 | height_10 |  |  | |
| rs1044252 | 1 | 176813774 | C | 0.013992 | 0.001675 | 6.56E-17 | 332021 | 0.431744 | height_10 |  |  | |
| rs28459747 | 1 | 218763810 | G | 0.01501 | 0.002588 | 6.64E-09 | 332021 | 0.118862 | height_10 |  |  | |
| rs9442571 | 1 | 9349611 | A | 0.015348 | 0.002483 | 6.36E-10 | 332021 | 0.129357 | height_10 |  |  | |
| rs34517439 | 1 | 78450517 | A | 0.026171 | 0.002542 | 7.40E-25 | 332021 | 0.125765 | height_10 |  |  | |
| rs4338381 | 1 | 103572927 | G | 0.010101 | 0.001721 | 4.40E-09 | 332021 | 0.368143 | height_10 |  |  | |
| rs11581328 | 1 | 8507773 | A | 0.013271 | 0.002074 | 1.56E-10 | 332021 | 0.208664 | height_10 |  |  | |
| rs2843152 | 1 | 2245570 | G | 0.011646 | 0.001891 | 7.29E-10 | 332021 | 0.736062 | height_10 |  |  | |
| rs2782641 | 1 | 44013355 | A | -0.01318 | 0.001709 | 1.26E-14 | 332021 | 0.613307 | height_10 |  |  | |
| rs1514177 | 1 | 74991402 | G | -0.00979 | 0.001676 | 5.29E-09 | 332021 | 0.57506 | height_10 |  |  | |
| rs11466399 | 1 | 218591623 | A | 0.019407 | 0.001838 | 4.60E-26 | 332021 | 0.286375 | height_10 |  |  | |
| rs1057079 | 1 | 11205058 | T | -0.01351 | 0.001896 | 1.03E-12 | 332021 | 0.742793 | height_10 |  |  | |
| rs35915186 | 1 | 224654623 | C | -0.01129 | 0.002037 | 2.94E-08 | 332021 | 0.213463 | height_10 |  |  | |
| rs138309741 | 1 | 150512792 | A | 0.027956 | 0.004275 | 6.22E-11 | 332021 | 0.04023 | height_10 |  |  | |
| rs4927132 | 1 | 54953404 | T | -0.01765 | 0.002778 | 2.09E-10 | 332021 | 0.100255 | height_10 |  |  | |
| rs7530780 | 1 | 93130268 | T | 0.010412 | 0.001718 | 1.36E-09 | 332021 | 0.633366 | height_10 |  |  | |
| rs2378436 | 1 | 221237584 | A | -0.0094 | 0.001673 | 1.92E-08 | 332021 | 0.559865 | height_10 |  |  | |
| rs7540754 | 1 | 227850588 | A | -0.02231 | 0.002203 | 4.17E-24 | 332021 | 0.172742 | height_10 |  |  | |
| rs11205303 | 1 | 149906413 | C | 0.03322 | 0.001687 | 2.55E-86 | 332021 | 0.409126 | height_10 |  |  | |
| rs58670122 | 1 | 22492613 | G | -0.01435 | 0.002407 | 2.50E-09 | 332021 | 0.141692 | height_10 |  |  | |
| rs12561781 | 1 | 9538654 | C | 0.014026 | 0.001774 | 2.64E-15 | 332021 | 0.349753 | height_10 |  |  | |
| rs35184161 | 1 | 26738382 | C | 0.024495 | 0.004115 | 2.65E-09 | 332021 | 0.0444752 | height_10 |  |  | |
| rs12129705 | 1 | 120191044 | T | -0.01577 | 0.002521 | 3.96E-10 | 332021 | 0.127726 | height_10 |  |  | |
| rs2275085 | 1 | 46085852 | T | -0.0103 | 0.001807 | 1.17E-08 | 332021 | 0.697954 | height_10 |  |  | |
| rs212526 | 1 | 21584941 | C | 0.017179 | 0.001692 | 3.20E-24 | 332021 | 0.601789 | height_10 |  |  | |
| rs10914505 | 1 | 32393578 | T | 0.019733 | 0.002446 | 7.14E-16 | 332021 | 0.132601 | height_10 |  |  | |
| rs12141730 | 1 | 40809127 | A | -0.01327 | 0.002244 | 3.37E-09 | 332021 | 0.168194 | height_10 |  |  | |
| rs2885697 | 1 | 41544279 | T | -0.02595 | 0.001755 | 1.96E-49 | 332021 | 0.664376 | height_10 |  |  | |
| rs12031920 | 1 | 51109269 | A | 0.011754 | 0.001683 | 2.85E-12 | 332021 | 0.416881 | height_10 |  |  | |
| rs1360504 | 1 | 56627905 | A | 0.013051 | 0.002105 | 5.66E-10 | 332021 | 0.192263 | height_10 |  |  | |
| rs448008 | 1 | 86810653 | A | 0.015437 | 0.002457 | 3.33E-10 | 332021 | 0.131701 | height_10 |  |  | |
| rs4233334 | 1 | 89147213 | T | 0.011589 | 0.001661 | 2.99E-12 | 332021 | 0.537549 | height_10 |  |  | |
| rs6428799 | 1 | 119680997 | A | -0.00995 | 0.00167 | 2.54E-09 | 332021 | 0.556132 | height_10 |  |  | |
| rs1926872 | 1 | 184018475 | C | 0.025878 | 0.001738 | 4.15E-50 | 332021 | 0.348847 | height_10 |  |  | |
| rs76306191 | 1 | 155006451 | G | 0.013953 | 0.002049 | 9.87E-12 | 332021 | 0.206046 | height_10 |  |  | |
| rs10489290 | 1 | 172223403 | A | 0.025775 | 0.002025 | 4.08E-37 | 332021 | 0.21299 | height_10 |  |  | |
| rs17515718 | 1 | 219041961 | A | -0.01107 | 0.001784 | 5.44E-10 | 332021 | 0.321288 | height_10 |  |  | |
| rs12031691 | 1 | 19750909 | T | -0.01097 | 0.001747 | 3.40E-10 | 332021 | 0.343664 | height_10 |  |  | |
| rs389548 | 1 | 26891697 | A | 0.012377 | 0.001885 | 5.19E-11 | 332021 | 0.738559 | height_10 |  |  | |
| rs1593512 | 1 | 93858374 | A | -0.01045 | 0.001714 | 1.08E-09 | 332021 | 0.631609 | height_10 |  |  | |
| rs545608 | 1 | 177899121 | C | 0.018157 | 0.002042 | 6.05E-19 | 332021 | 0.20814 | height_10 |  |  | |
| rs823130 | 1 | 205714372 | T | -0.01434 | 0.001677 | 1.21E-17 | 332021 | 0.434223 | height_10 |  |  | |
| rs12045638 | 1 | 183295069 | T | -0.01635 | 0.002664 | 8.36E-10 | 332021 | 0.109266 | height_10 |  |  | |
| rs2970599 | 1 | 212183497 | A | 0.01057 | 0.001682 | 3.28E-10 | 332021 | 0.588337 | height_10 |  |  | |
| rs113522281 | 1 | 219769141 | T | -0.01028 | 0.001706 | 1.66E-09 | 332021 | 0.385974 | height_10 |  |  | |
| rs833149 | 2 | 183223013 | C | -0.01054 | 0.001679 | 3.44E-10 | 332021 | 0.576256 | height_10 |  |  | |
| rs13005615 | 2 | 172238433 | G | 0.012454 | 0.001887 | 4.14E-11 | 332021 | 0.265526 | height_10 |  |  | |
| rs981938 | 2 | 218275818 | A | 0.010808 | 0.001787 | 1.46E-09 | 332021 | 0.685456 | height_10 |  |  | |
| rs12993339 | 2 | 219202624 | T | -0.0165 | 0.001898 | 3.45E-18 | 332021 | 0.260247 | height_10 |  |  | |
| rs17511102 | 2 | 37960613 | T | 0.02458 | 0.002893 | 1.95E-17 | 332021 | 0.0906053 | height_10 |  |  | |
| rs59969844 | 2 | 12196833 | G | 0.012644 | 0.00223 | 1.43E-08 | 332021 | 0.168865 | height_10 |  |  | |
| rs1344840 | 2 | 25070645 | A | 0.017544 | 0.001898 | 2.43E-20 | 332021 | 0.262824 | height_10 |  |  | |
| rs6756738 | 2 | 47008964 | T | -0.01386 | 0.001768 | 4.62E-15 | 332021 | 0.337193 | height_10 |  |  | |
| rs7571971 | 2 | 88895351 | C | -0.0132 | 0.00184 | 7.43E-13 | 332021 | 0.718101 | height_10 |  |  | |
| rs283468 | 2 | 233658309 | T | -0.01235 | 0.001743 | 1.39E-12 | 332021 | 0.34688 | height_10 |  |  | |
| rs116376456 | 2 | 227326633 | T | -0.0506 | 0.006454 | 4.55E-15 | 332021 | 0.0172791 | height_10 |  |  | |
| rs79057767 | 2 | 233093733 | G | 0.051436 | 0.004721 | 1.24E-27 | 332021 | 0.0319633 | height_10 |  |  | |
| rs7599010 | 2 | 219661840 | C | -0.01103 | 0.001664 | 3.39E-11 | 332021 | 0.508092 | height_10 |  |  | |
| rs2252488 | 2 | 217857450 | C | 0.010108 | 0.001793 | 1.72E-08 | 332021 | 0.682687 | height_10 |  |  | |
| rs12475061 | 2 | 9679762 | T | 0.009067 | 0.001657 | 4.44E-08 | 332021 | 0.509778 | height_10 |  |  | |
| rs113386058 | 2 | 20143831 | T | -0.04471 | 0.005007 | 4.32E-19 | 332021 | 0.0294529 | height_10 |  |  | |
| rs4665635 | 2 | 23932528 | G | 0.01574 | 0.001657 | 2.14E-21 | 332021 | 0.496422 | height_10 |  |  | |
| rs6739187 | 2 | 25513652 | A | -0.00927 | 0.001688 | 3.99E-08 | 332021 | 0.431524 | height_10 |  |  | |
| rs17400325 | 2 | 178565913 | C | 0.04534 | 0.004174 | 1.75E-27 | 332021 | 0.0410336 | height_10 |  |  | |
| rs2629047 | 2 | 225047774 | A | -0.01313 | 0.001697 | 1.02E-14 | 332021 | 0.395731 | height_10 |  |  | |
| rs13430869 | 2 | 218146818 | T | 0.012182 | 0.001912 | 1.89E-10 | 332021 | 0.747688 | height_10 |  |  | |
| rs10933368 | 2 | 232275273 | T | -0.01506 | 0.002694 | 2.25E-08 | 332021 | 0.109324 | height_10 |  |  | |
| rs72951802 | 2 | 220006130 | A | -0.02067 | 0.00253 | 3.15E-16 | 332021 | 0.122445 | height_10 |  |  | |
| rs11124931 | 2 | 43599252 | A | 0.013983 | 0.001662 | 4.06E-17 | 332021 | 0.526204 | height_10 |  |  | |
| rs59985551 | 2 | 56106928 | T | -0.03872 | 0.001986 | 1.27E-84 | 332021 | 0.224341 | height_10 |  |  | |
| rs12615742 | 2 | 37995727 | T | 0.015299 | 0.001698 | 2.05E-19 | 332021 | 0.505196 | height_10 |  |  | |
| rs12619985 | 2 | 46641627 | C | 0.010724 | 0.001662 | 1.11E-10 | 332021 | 0.522763 | height_10 |  |  | |
| rs41393947 | 2 | 56011517 | A | 0.020309 | 0.002384 | 1.62E-17 | 332021 | 0.142797 | height_10 |  |  | |
| rs5268 | 2 | 232790053 | T | 0.014397 | 0.001734 | 1.00E-16 | 332021 | 0.634661 | height_10 |  |  | |
| rs79264696 | 2 | 241925843 | T | 0.033172 | 0.005669 | 4.88E-09 | 332021 | 0.0237901 | height_10 |  |  | |
| rs707718 | 2 | 72356449 | T | 0.01343 | 0.002187 | 8.25E-10 | 332021 | 0.18245 | height_10 |  |  | |
| rs1545552 | 2 | 33360338 | G | 0.01657 | 0.001831 | 1.45E-19 | 332021 | 0.707682 | height_10 |  |  | |
| rs7559547 | 2 | 615627 | T | 0.026294 | 0.002183 | 2.08E-33 | 332021 | 0.825919 | height_10 |  |  | |
| rs17713568 | 2 | 242132 | A | 0.014956 | 0.001737 | 7.24E-18 | 332021 | 0.350268 | height_10 |  |  | |
| rs62106258 | 2 | 417167 | C | -0.03906 | 0.00386 | 4.62E-24 | 332021 | 0.0484669 | height_10 |  |  | |
| rs3116216 | 2 | 233091531 | C | -0.03151 | 0.002813 | 4.18E-29 | 332021 | 0.0963215 | height_10 |  |  | |
| rs34122762 | 2 | 199744537 | A | 0.01206 | 0.001942 | 5.34E-10 | 332021 | 0.237284 | height_10 |  |  | |
| rs35539500 | 2 | 121613995 | G | -0.01551 | 0.002214 | 2.49E-12 | 332021 | 0.168621 | height_10 |  |  | |
| rs17551974 | 2 | 142293146 | A | -0.01229 | 0.002132 | 8.09E-09 | 332021 | 0.185487 | height_10 |  |  | |
| rs6737373 | 2 | 61498038 | G | 0.010681 | 0.0017 | 3.29E-10 | 332021 | 0.391494 | height_10 |  |  | |
| rs111258456 | 2 | 69724649 | G | 0.016725 | 0.002173 | 1.38E-14 | 332021 | 0.176249 | height_10 |  |  | |
| rs12712912 | 2 | 44396235 | G | -0.02459 | 0.002657 | 2.14E-20 | 332021 | 0.890092 | height_10 |  |  | |
| rs1986260 | 2 | 55937866 | G | 0.031842 | 0.003634 | 1.93E-18 | 332021 | 0.0569056 | height_10 |  |  | |
| rs4852252 | 2 | 71539301 | C | -0.01746 | 0.001672 | 1.59E-25 | 332021 | 0.56425 | height_10 |  |  | |
| rs6717452 | 2 | 10198113 | A | -0.01073 | 0.001677 | 1.56E-10 | 332021 | 0.563679 | height_10 |  |  | |
| rs10204062 | 2 | 172027579 | A | -0.01471 | 0.001721 | 1.26E-17 | 332021 | 0.376536 | height_10 |  |  | |
| rs1561369 | 2 | 183702964 | A | 0.015476 | 0.002559 | 1.46E-09 | 332021 | 0.119831 | height_10 |  |  | |
| rs10172196 | 2 | 36780549 | A | 0.013389 | 0.001807 | 1.27E-13 | 332021 | 0.304839 | height_10 |  |  | |
| rs1446245 | 2 | 114033150 | A | -0.00933 | 0.001673 | 2.44E-08 | 332021 | 0.562011 | height_10 |  |  | |
| rs114707893 | 2 | 97566051 | T | -0.05102 | 0.005345 | 1.36E-21 | 332021 | 0.0247347 | height_10 |  |  | |
| rs11689727 | 2 | 25458100 | A | -0.02072 | 0.001762 | 6.67E-32 | 332021 | 0.33211 | height_10 |  |  | |
| rs7588894 | 2 | 33566412 | A | -0.01128 | 0.001676 | 1.71E-11 | 332021 | 0.466837 | height_10 |  |  | |
| rs72771625 | 2 | 10400557 | T | -0.02115 | 0.002841 | 9.55E-14 | 332021 | 0.0951604 | height_10 |  |  | |
| rs1260326 | 2 | 27730940 | C | 0.014649 | 0.001696 | 5.72E-18 | 332021 | 0.607251 | height_10 |  |  | |
| rs4675801 | 2 | 242493511 | T | -0.01196 | 0.001669 | 7.49E-13 | 332021 | 0.455574 | height_10 |  |  | |
| rs111277591 | 2 | 225413703 | C | 0.039554 | 0.006476 | 1.01E-09 | 332021 | 0.0174645 | height_10 |  |  | |
| rs149870557 | 2 | 233199484 | T | 0.029776 | 0.003055 | 1.91E-22 | 332021 | 0.0856203 | height_10 |  |  | |
| rs886811 | 2 | 242397292 | A | 0.012903 | 0.001682 | 1.72E-14 | 332021 | 0.418744 | height_10 |  |  | |
| rs12466581 | 2 | 233093315 | G | -0.04768 | 0.006113 | 6.23E-15 | 332021 | 0.0187323 | height_10 |  |  | |
| rs76152268 | 2 | 216303813 | C | -0.03896 | 0.006939 | 1.97E-08 | 332021 | 0.0158082 | height_10 |  |  | |
| rs934247 | 2 | 218598202 | A | 0.034093 | 0.00535 | 1.86E-10 | 332021 | 0.97535 | height_10 |  |  | |
| rs1879244 | 3 | 184046355 | T | -0.01311 | 0.001906 | 6.10E-12 | 332021 | 0.744741 | height_10 |  |  | |
| rs62274129 | 3 | 156856967 | T | -0.01226 | 0.001923 | 1.84E-10 | 332021 | 0.252556 | height_10 |  |  | |
| rs3172494 | 3 | 48731487 | T | 0.015612 | 0.002696 | 6.99E-09 | 332021 | 0.106625 | height_10 |  |  | |
| rs6445224 | 3 | 61548285 | T | 0.010375 | 0.001817 | 1.14E-08 | 332021 | 0.706208 | height_10 |  |  | |
| rs4684158 | 3 | 13809901 | T | -0.01091 | 0.001812 | 1.73E-09 | 332021 | 0.685988 | height_10 |  |  | |
| rs9826470 | 3 | 38019428 | T | 0.010957 | 0.001927 | 1.30E-08 | 332021 | 0.249213 | height_10 |  |  | |
| rs9809116 | 3 | 72397279 | G | -0.01944 | 0.0017 | 2.77E-30 | 332021 | 0.40736 | height_10 |  |  | |
| rs2871960 | 3 | 141121814 | C | 0.03665 | 0.001666 | 3.30E-107 | 332021 | 0.447942 | height_10 |  |  | |
| rs75938095 | 3 | 157638596 | A | 0.023949 | 0.00427 | 2.04E-08 | 332021 | 0.0395775 | height_10 |  |  | |
| rs2597513 | 3 | 13555836 | T | -0.02068 | 0.002726 | 3.26E-14 | 332021 | 0.896829 | height_10 |  |  | |
| rs56273245 | 3 | 47314496 | G | -0.01803 | 0.001988 | 1.20E-19 | 332021 | 0.237805 | height_10 |  |  | |
| rs7621604 | 3 | 61557718 | G | -0.01596 | 0.001669 | 1.17E-21 | 332021 | 0.445442 | height_10 |  |  | |
| rs2581828 | 3 | 53133149 | G | -0.01471 | 0.001682 | 2.21E-18 | 332021 | 0.589653 | height_10 |  |  | |
| rs6762578 | 3 | 128992047 | A | 0.019269 | 0.002004 | 6.97E-22 | 332021 | 0.778186 | height_10 |  |  | |
| rs9824067 | 3 | 190849536 | A | -0.01405 | 0.002386 | 3.90E-09 | 332021 | 0.85825 | height_10 |  |  | |
| rs1716838 | 3 | 120106521 | G | -0.01019 | 0.001825 | 2.37E-08 | 332021 | 0.292817 | height_10 |  |  | |
| rs1513304 | 3 | 99269750 | C | 0.014093 | 0.001822 | 1.05E-14 | 332021 | 0.2931 | height_10 |  |  | |
| rs6762851 | 3 | 56686329 | C | -0.00992 | 0.001732 | 1.01E-08 | 332021 | 0.35575 | height_10 |  |  | |
| rs61732778 | 3 | 187443314 | A | 0.023005 | 0.00322 | 9.09E-13 | 332021 | 0.0710233 | height_10 |  |  | |
| rs17365792 | 3 | 136497392 | G | -0.01515 | 0.001994 | 2.99E-14 | 332021 | 0.223172 | height_10 |  |  | |
| rs2268023 | 3 | 52819327 | A | -0.01017 | 0.001701 | 2.24E-09 | 332021 | 0.395523 | height_10 |  |  | |
| rs10937361 | 3 | 188359436 | C | 0.0105 | 0.00167 | 3.22E-10 | 332021 | 0.441036 | height_10 |  |  | |
| rs519384 | 3 | 172168507 | A | 0.027253 | 0.001835 | 6.65E-50 | 332021 | 0.289156 | height_10 |  |  | |
| rs1552485 | 3 | 12580283 | C | -0.01142 | 0.001664 | 6.78E-12 | 332021 | 0.53643 | height_10 |  |  | |
| rs9873478 | 3 | 30078271 | A | 0.010204 | 0.001667 | 9.31E-10 | 332021 | 0.547239 | height_10 |  |  | |
| rs9880232 | 3 | 185360578 | A | 0.01312 | 0.001821 | 5.80E-13 | 332021 | 0.701749 | height_10 |  |  | |
| rs7652177 | 3 | 171969077 | G | 0.01877 | 0.001657 | 9.89E-30 | 332021 | 0.504597 | height_10 |  |  | |
| rs2270894 | 3 | 9975386 | G | -0.02017 | 0.002133 | 3.19E-21 | 332021 | 0.205074 | height_10 |  |  | |
| rs10935113 | 3 | 134173091 | C | 0.01169 | 0.00178 | 5.11E-11 | 332021 | 0.675785 | height_10 |  |  | |
| rs2115959 | 3 | 169117792 | C | 0.009335 | 0.001668 | 2.21E-08 | 332021 | 0.547386 | height_10 |  |  | |
| rs9310019 | 3 | 86752049 | T | 0.012734 | 0.001743 | 2.75E-13 | 332021 | 0.379768 | height_10 |  |  | |
| rs2194411 | 3 | 185548663 | A | 0.03145 | 0.002509 | 5.05E-36 | 332021 | 0.127999 | height_10 |  |  | |
| rs4339203 | 4 | 10462106 | A | -0.01119 | 0.001976 | 1.47E-08 | 332021 | 0.227395 | height_10 |  |  | |
| rs16896245 | 4 | 18005608 | T | -0.03636 | 0.002277 | 2.32E-57 | 332021 | 0.156918 | height_10 |  |  | |
| rs2610986 | 4 | 18037231 | T | -0.01595 | 0.00179 | 5.08E-19 | 332021 | 0.65789 | height_10 |  |  | |
| rs9884546 | 4 | 48566634 | C | 0.011607 | 0.001662 | 2.91E-12 | 332021 | 0.510485 | height_10 |  |  | |
| rs140557441 | 4 | 87659122 | T | -0.04425 | 0.006646 | 2.77E-11 | 332021 | 0.0162397 | height_10 |  |  | |
| rs2647257 | 4 | 106199505 | T | -0.01073 | 0.001701 | 2.84E-10 | 332021 | 0.387943 | height_10 |  |  | |
| rs36227682 | 4 | 146053033 | A | 0.025706 | 0.004669 | 3.68E-08 | 332021 | 0.0356823 | height_10 |  |  | |
| rs114734516 | 4 | 146127689 | C | -0.02582 | 0.003879 | 2.81E-11 | 332021 | 0.0482727 | height_10 |  |  | |
| rs13152701 | 4 | 122751061 | A | -0.0108 | 0.001701 | 2.17E-10 | 332021 | 0.395461 | height_10 |  |  | |
| rs34471118 | 4 | 83273140 | G | -0.01106 | 0.001998 | 3.10E-08 | 332021 | 0.220663 | height_10 |  |  | |
| rs146962627 | 4 | 145875495 | C | -0.04487 | 0.005239 | 1.08E-17 | 332021 | 0.026692 | height_10 |  |  | |
| rs10015974 | 4 | 184196254 | G | 0.013685 | 0.00194 | 1.76E-12 | 332021 | 0.758807 | height_10 |  |  | |
| rs2575572 | 4 | 145552729 | C | -0.01636 | 0.001656 | 5.17E-23 | 332021 | 0.492584 | height_10 |  |  | |
| rs139868653 | 4 | 105931075 | T | -0.03134 | 0.005613 | 2.36E-08 | 332021 | 0.0234298 | height_10 |  |  | |
| rs1838043 | 4 | 152411462 | C | -0.01078 | 0.001682 | 1.49E-10 | 332021 | 0.583983 | height_10 |  |  | |
| rs11731421 | 4 | 1749160 | A | 0.01614 | 0.00176 | 4.82E-20 | 332021 | 0.332928 | height_10 |  |  | |
| rs11722554 | 4 | 5016883 | A | -0.0261 | 0.004384 | 2.62E-09 | 332021 | 0.037125 | height_10 |  |  | |
| rs16888425 | 4 | 13263176 | T | 0.010257 | 0.001766 | 6.29E-09 | 332021 | 0.329406 | height_10 |  |  | |
| rs13106087 | 4 | 145566864 | C | 0.042754 | 0.002207 | 1.45E-83 | 332021 | 0.830084 | height_10 |  |  | |
| rs73793175 | 4 | 1255460 | A | -0.0236 | 0.003639 | 8.78E-11 | 332021 | 0.0548326 | height_10 |  |  | |
| rs6446841 | 4 | 73437856 | G | -0.01751 | 0.001659 | 4.92E-26 | 332021 | 0.524763 | height_10 |  |  | |
| rs73165969 | 4 | 57750725 | A | 0.017681 | 0.002158 | 2.57E-16 | 332021 | 0.182696 | height_10 |  |  | |
| rs11947277 | 4 | 3473751 | T | -0.01109 | 0.0019 | 5.33E-09 | 332021 | 0.257624 | height_10 |  |  | |
| rs1704155 | 4 | 40057909 | C | 0.017251 | 0.002571 | 1.94E-11 | 332021 | 0.882219 | height_10 |  |  | |
| rs1901568 | 4 | 82187030 | C | 0.020236 | 0.001791 | 1.36E-29 | 332021 | 0.313317 | height_10 |  |  | |
| rs28783740 | 4 | 12911495 | T | -0.0128 | 0.001659 | 1.20E-14 | 332021 | 0.506266 | height_10 |  |  | |
| rs3796734 | 4 | 8599467 | C | -0.01105 | 0.001679 | 4.79E-11 | 332021 | 0.435013 | height_10 |  |  | |
| rs35746105 | 5 | 108125740 | G | -0.02799 | 0.003136 | 4.50E-19 | 332021 | 0.0758528 | height_10 |  |  | |
| rs72793280 | 5 | 131562900 | T | 0.018809 | 0.001672 | 2.29E-29 | 332021 | 0.457535 | height_10 |  |  | |
| rs34651 | 5 | 72144005 | T | -0.02052 | 0.003046 | 1.64E-11 | 332021 | 0.918139 | height_10 |  |  | |
| rs1394821 | 5 | 101935100 | A | 0.013122 | 0.001901 | 5.06E-12 | 332021 | 0.255371 | height_10 |  |  | |
| rs6874142 | 5 | 172753555 | G | 0.019823 | 0.00275 | 5.65E-13 | 332021 | 0.111485 | height_10 |  |  | |
| rs244711 | 5 | 176509193 | T | 0.024621 | 0.001915 | 7.96E-38 | 332021 | 0.687357 | height_10 |  |  | |
| rs13183901 | 5 | 172990492 | C | -0.01461 | 0.001743 | 5.39E-17 | 332021 | 0.346387 | height_10 |  |  | |
| rs2059780 | 5 | 134522977 | A | 0.011432 | 0.001669 | 7.40E-12 | 332021 | 0.542844 | height_10 |  |  | |
| rs1533015 | 5 | 32995603 | C | 0.015917 | 0.001675 | 2.05E-21 | 332021 | 0.56856 | height_10 |  |  | |
| rs9716700 | 5 | 32711633 | A | -0.01993 | 0.002217 | 2.55E-19 | 332021 | 0.192017 | height_10 |  |  | |
| rs10079334 | 5 | 33311486 | T | 0.014623 | 0.001907 | 1.76E-14 | 332021 | 0.7465 | height_10 |  |  | |
| rs174047 | 5 | 142623288 | G | 0.009481 | 0.001667 | 1.29E-08 | 332021 | 0.536766 | height_10 |  |  | |
| rs3822593 | 5 | 178551299 | G | -0.01376 | 0.001688 | 3.59E-16 | 332021 | 0.582048 | height_10 |  |  | |
| rs6892884 | 5 | 171015623 | C | 0.012394 | 0.001789 | 4.33E-12 | 332021 | 0.689692 | height_10 |  |  | |
| rs11748619 | 5 | 179727399 | G | 0.014595 | 0.001699 | 8.83E-18 | 332021 | 0.608139 | height_10 |  |  | |
| rs11955153 | 5 | 170864548 | C | -0.0209 | 0.001956 | 1.16E-26 | 332021 | 0.235492 | height_10 |  |  | |
| rs31198 | 5 | 134372685 | C | -0.02038 | 0.001912 | 1.66E-26 | 332021 | 0.250054 | height_10 |  |  | |
| rs425102 | 5 | 77396400 | G | -0.01867 | 0.001933 | 4.68E-22 | 332021 | 0.241488 | height_10 |  |  | |
| rs440292 | 5 | 67609408 | G | 0.014811 | 0.001902 | 6.79E-15 | 332021 | 0.74501 | height_10 |  |  | |
| rs2972210 | 5 | 72658437 | C | -0.0094 | 0.001708 | 3.70E-08 | 332021 | 0.622159 | height_10 |  |  | |
| rs4489092 | 5 | 90420644 | T | -0.01147 | 0.001734 | 3.74E-11 | 332021 | 0.631815 | height_10 |  |  | |
| rs1388122 | 5 | 67100256 | G | 0.017817 | 0.002262 | 3.38E-15 | 332021 | 0.161255 | height_10 |  |  | |
| rs17475053 | 5 | 114995827 | G | 0.012601 | 0.001872 | 1.69E-11 | 332021 | 0.268684 | height_10 |  |  | |
| rs116302901 | 5 | 127802451 | A | 0.042386 | 0.007305 | 6.56E-09 | 332021 | 0.0130294 | height_10 |  |  | |
| rs35668185 | 5 | 168256455 | C | -0.02054 | 0.002045 | 9.45E-24 | 332021 | 0.206755 | height_10 |  |  | |
| rs11743919 | 5 | 156754251 | T | -0.01751 | 0.002023 | 5.01E-18 | 332021 | 0.212346 | height_10 |  |  | |
| rs1818782 | 5 | 39424628 | C | 0.011384 | 0.001756 | 8.96E-11 | 332021 | 0.656992 | height_10 |  |  | |
| rs407746 | 5 | 67323686 | G | 0.018336 | 0.002805 | 6.34E-11 | 332021 | 0.0964875 | height_10 |  |  | |
| rs11738691 | 5 | 54880229 | T | -0.01495 | 0.001756 | 1.70E-17 | 332021 | 0.662511 | height_10 |  |  | |
| rs1366594 | 5 | 88376061 | C | -0.01614 | 0.00166 | 2.44E-22 | 332021 | 0.46933 | height_10 |  |  | |
| rs10063744 | 5 | 95649772 | G | -0.01409 | 0.001843 | 2.09E-14 | 332021 | 0.284987 | height_10 |  |  | |
| rs4868125 | 5 | 171281875 | G | 0.020561 | 0.001708 | 2.21E-33 | 332021 | 0.580137 | height_10 |  |  | |
| rs246435 | 5 | 129045738 | G | -0.02545 | 0.003101 | 2.29E-16 | 332021 | 0.923174 | height_10 |  |  | |
| rs35779874 | 5 | 138869847 | G | -0.01539 | 0.001844 | 6.93E-17 | 332021 | 0.716822 | height_10 |  |  | |
| rs75475720 | 5 | 108200114 | G | 0.023248 | 0.003005 | 1.03E-14 | 332021 | 0.0834433 | height_10 |  |  | |
| rs3811964 | 5 | 32770099 | T | 0.021081 | 0.001892 | 7.89E-29 | 332021 | 0.258643 | height_10 |  |  | |
| rs33852 | 5 | 171189571 | G | 0.015003 | 0.001772 | 2.53E-17 | 332021 | 0.322296 | height_10 |  |  | |
| rs183578486 | 5 | 176532241 | A | -0.0387 | 0.005085 | 2.74E-14 | 332021 | 0.0274215 | height_10 |  |  | |
| rs13156484 | 5 | 122653399 | A | -0.00971 | 0.001676 | 7.08E-09 | 332021 | 0.470873 | height_10 |  |  | |
| rs72780278 | 5 | 126174945 | C | -0.01644 | 0.002971 | 3.13E-08 | 332021 | 0.0856127 | height_10 |  |  | |
| rs112339535 | 5 | 32807390 | G | -0.03409 | 0.005047 | 1.44E-11 | 332021 | 0.0279449 | height_10 |  |  | |
| rs116176947 | 5 | 64693608 | A | -0.02784 | 0.004282 | 7.95E-11 | 332021 | 0.0417839 | height_10 |  |  | |
| rs6899155 | 5 | 36786459 | C | -0.0114 | 0.001678 | 1.11E-11 | 332021 | 0.445196 | height_10 |  |  | |
| rs34742008 | 5 | 42684956 | A | -0.01442 | 0.001668 | 5.42E-18 | 332021 | 0.448891 | height_10 |  |  | |
| rs33321 | 5 | 56206073 | T | 0.010371 | 0.001738 | 2.43E-09 | 332021 | 0.64746 | height_10 |  |  | |
| rs41271299 | 6 | 19839415 | T | 0.057449 | 0.003744 | 3.97E-53 | 332021 | 0.0517069 | height_10 |  |  | |
| rs13194984 | 6 | 26500563 | T | 0.0231 | 0.002356 | 1.08E-22 | 332021 | 0.144103 | height_10 |  |  | |
| rs139901797 | 6 | 158898974 | C | -0.02427 | 0.003533 | 6.48E-12 | 332021 | 0.0587294 | height_10 |  |  | |
| rs1319012 | 6 | 41852616 | A | -0.02748 | 0.003241 | 2.27E-17 | 332021 | 0.925522 | height_10 |  |  | |
| rs3800228 | 6 | 108996748 | G | 0.01443 | 0.001847 | 5.53E-15 | 332021 | 0.716575 | height_10 |  |  | |
| rs2026490 | 6 | 34110880 | A | 0.03353 | 0.006083 | 3.55E-08 | 332021 | 0.0191163 | height_10 |  |  | |
| rs6457814 | 6 | 35317603 | G | -0.05083 | 0.005251 | 3.65E-22 | 332021 | 0.0254879 | height_10 |  |  | |
| rs7740107 | 6 | 130374461 | A | -0.02617 | 0.001877 | 3.34E-44 | 332021 | 0.735506 | height_10 |  |  | |
| rs6453829 | 6 | 76268509 | T | -0.01434 | 0.002405 | 2.50E-09 | 332021 | 0.137257 | height_10 |  |  | |
| rs72848512 | 6 | 36182858 | A | -0.01062 | 0.001822 | 5.54E-09 | 332021 | 0.296105 | height_10 |  |  | |
| rs33966734 | 6 | 41903798 | A | -0.04964 | 0.007339 | 1.36E-11 | 332021 | 0.0132874 | height_10 |  |  | |
| rs7745990 | 6 | 47488387 | A | 0.012898 | 0.001791 | 6.04E-13 | 332021 | 0.688854 | height_10 |  |  | |
| rs524492 | 6 | 44796596 | T | -0.01268 | 0.001869 | 1.16E-11 | 332021 | 0.269602 | height_10 |  |  | |
| rs9386799 | 6 | 109654601 | A | -0.01059 | 0.001676 | 2.62E-10 | 332021 | 0.44759 | height_10 |  |  | |
| rs512840 | 6 | 116712348 | A | 0.010598 | 0.001667 | 2.05E-10 | 332021 | 0.535516 | height_10 |  |  | |
| rs71566925 | 6 | 81473826 | A | 0.021122 | 0.002554 | 1.34E-16 | 332021 | 0.124045 | height_10 |  |  | |
| rs76453248 | 6 | 158918146 | G | 0.014548 | 0.002536 | 9.71E-09 | 332021 | 0.121006 | height_10 |  |  | |
| rs9496369 | 6 | 142724918 | T | -0.02943 | 0.00184 | 1.49E-57 | 332021 | 0.28353 | height_10 |  |  | |
| rs12526645 | 6 | 85455863 | A | -0.01007 | 0.001781 | 1.57E-08 | 332021 | 0.318975 | height_10 |  |  | |
| rs2763263 | 6 | 168814392 | A | -0.01987 | 0.001947 | 1.84E-24 | 332021 | 0.243119 | height_10 |  |  | |
| rs12201553 | 6 | 7719940 | G | 0.023781 | 0.001658 | 1.22E-46 | 332021 | 0.479296 | height_10 |  |  | |
| rs17143141 | 6 | 7801217 | A | 0.016972 | 0.002273 | 8.23E-14 | 332021 | 0.157638 | height_10 |  |  | |
| rs10948374 | 6 | 47647221 | T | 0.011179 | 0.002046 | 4.68E-08 | 332021 | 0.208312 | height_10 |  |  | |
| rs9392366 | 6 | 2101913 | A | -0.0099 | 0.001777 | 2.53E-08 | 332021 | 0.330292 | height_10 |  |  | |
| rs2780226 | 6 | 34199092 | T | -0.04323 | 0.002914 | 9.07E-50 | 332021 | 0.911435 | height_10 |  |  | |
| rs139996541 | 6 | 34224226 | T | 0.032159 | 0.005257 | 9.51E-10 | 332021 | 0.0264073 | height_10 |  |  | |
| rs75307579 | 6 | 34167510 | G | 0.017262 | 0.002805 | 7.57E-10 | 332021 | 0.0964356 | height_10 |  |  | |
| rs9379084 | 6 | 7231843 | A | -0.01639 | 0.002675 | 8.98E-10 | 332021 | 0.117158 | height_10 |  |  | |
| rs34574518 | 6 | 142406234 | G | -0.01305 | 0.002041 | 1.60E-10 | 332021 | 0.209056 | height_10 |  |  | |
| rs267177 | 6 | 7828542 | T | 0.009456 | 0.001709 | 3.18E-08 | 332021 | 0.390634 | height_10 |  |  | |
| rs9274351 | 6 | 32632425 | A | -0.01541 | 0.002257 | 8.82E-12 | 332021 | 0.18632 | height_10 |  |  | |
| rs2206030 | 6 | 35404354 | C | 0.020049 | 0.001932 | 3.14E-25 | 332021 | 0.751948 | height_10 |  |  | |
| rs9341808 | 6 | 80953257 | A | -0.01194 | 0.001664 | 7.43E-13 | 332021 | 0.480173 | height_10 |  |  | |
| rs2008027 | 6 | 126052359 | G | 0.011293 | 0.001665 | 1.20E-11 | 332021 | 0.519214 | height_10 |  |  | |
| rs17789218 | 6 | 100600097 | C | 0.011449 | 0.001929 | 2.95E-09 | 332021 | 0.244053 | height_10 |  |  | |
| rs9378185 | 6 | 28606662 | A | 0.011774 | 0.00166 | 1.34E-12 | 332021 | 0.523488 | height_10 |  |  | |
| rs1233421 | 6 | 29501514 | A | 0.009668 | 0.001656 | 5.25E-09 | 332021 | 0.494454 | height_10 |  |  | |
| rs2814985 | 6 | 34548296 | T | 0.025993 | 0.002436 | 1.41E-26 | 332021 | 0.137148 | height_10 |  |  | |
| rs668871 | 6 | 160769811 | T | 0.012221 | 0.001662 | 1.93E-13 | 332021 | 0.468385 | height_10 |  |  | |
| rs991946 | 6 | 166329862 | T | -0.01073 | 0.001661 | 1.02E-10 | 332021 | 0.478468 | height_10 |  |  | |
| rs9395644 | 6 | 50923637 | G | -0.01836 | 0.002734 | 1.91E-11 | 332021 | 0.102551 | height_10 |  |  | |
| rs9350902 | 6 | 81773990 | C | -0.01376 | 0.001815 | 3.38E-14 | 332021 | 0.316424 | height_10 |  |  | |
| rs114770584 | 6 | 2263605 | T | 0.036563 | 0.006653 | 3.90E-08 | 332021 | 0.0159965 | height_10 |  |  | |
| rs140013811 | 6 | 32089436 | T | -0.03861 | 0.006062 | 1.90E-10 | 332021 | 0.0205131 | height_10 |  |  | |
| rs6902771 | 6 | 152157881 | T | 0.011539 | 0.001659 | 3.50E-12 | 332021 | 0.462804 | height_10 |  |  | |
| rs544148 | 6 | 117466494 | G | 0.009457 | 0.001686 | 2.02E-08 | 332021 | 0.583727 | height_10 |  |  | |
| rs62396185 | 6 | 26180634 | C | -0.02726 | 0.001901 | 1.30E-46 | 332021 | 0.256464 | height_10 |  |  | |
| rs6903066 | 6 | 131332166 | T | 0.016615 | 0.002045 | 4.49E-16 | 332021 | 0.207701 | height_10 |  |  | |
| rs72502585 | 6 | 31381486 | T | -0.01562 | 0.002214 | 1.72E-12 | 332021 | 0.170079 | height_10 |  |  | |
| rs1998264 | 6 | 8589775 | A | 0.010276 | 0.001827 | 1.85E-08 | 332021 | 0.710158 | height_10 |  |  | |
| rs12209223 | 6 | 76164589 | A | 0.032483 | 0.002747 | 2.94E-32 | 332021 | 0.102158 | height_10 |  |  | |
| rs3818695 | 6 | 169363121 | T | -0.01074 | 0.001689 | 2.05E-10 | 332021 | 0.407415 | height_10 |  |  | |
| rs4897180 | 6 | 126727950 | T | 0.033439 | 0.001664 | 9.64E-90 | 332021 | 0.454313 | height_10 |  |  | |
| rs62433460 | 6 | 141663664 | A | 0.010856 | 0.001897 | 1.04E-08 | 332021 | 0.255727 | height_10 |  |  | |
| rs210627 | 6 | 117876956 | A | -0.01182 | 0.001884 | 3.60E-10 | 332021 | 0.736178 | height_10 |  |  | |
| rs7769838 | 6 | 33641956 | C | 0.01041 | 0.001834 | 1.38E-08 | 332021 | 0.711175 | height_10 |  |  | |
| rs4388309 | 6 | 31561639 | A | -0.02098 | 0.002396 | 2.06E-18 | 332021 | 0.138997 | height_10 |  |  | |
| rs622871 | 6 | 31878495 | G | -0.02209 | 0.001788 | 4.39E-35 | 332021 | 0.685639 | height_10 |  |  | |
| rs148190310 | 7 | 148564138 | A | 0.041064 | 0.005176 | 2.13E-15 | 332021 | 0.0267987 | height_10 |  |  | |
| rs73277029 | 7 | 25591710 | T | 0.013957 | 0.002343 | 2.58E-09 | 332021 | 0.151425 | height_10 |  |  | |
| rs849141 | 7 | 28185091 | G | -0.02468 | 0.001824 | 1.09E-41 | 332021 | 0.708641 | height_10 |  |  | |
| rs315854 | 7 | 33916334 | T | -0.00927 | 0.001699 | 4.90E-08 | 332021 | 0.39988 | height_10 |  |  | |
| rs12666660 | 7 | 65525261 | G | 0.00981 | 0.001758 | 2.40E-08 | 332021 | 0.333844 | height_10 |  |  | |
| rs73144827 | 7 | 73319690 | A | -0.01499 | 0.002549 | 4.06E-09 | 332021 | 0.123064 | height_10 |  |  | |
| rs3812277 | 7 | 135067234 | C | 0.012876 | 0.00174 | 1.35E-13 | 332021 | 0.650641 | height_10 |  |  | |
| rs2854746 | 7 | 45960645 | C | -0.01293 | 0.001704 | 3.19E-14 | 332021 | 0.399952 | height_10 |  |  | |
| rs798488 | 7 | 2802522 | C | -0.02464 | 0.00181 | 3.17E-42 | 332021 | 0.300367 | height_10 |  |  | |
| rs11978390 | 7 | 19649160 | G | 0.034187 | 0.005116 | 2.35E-11 | 332021 | 0.0273044 | height_10 |  |  | |
| rs6944563 | 7 | 134389713 | T | 0.010218 | 0.001737 | 4.05E-09 | 332021 | 0.352379 | height_10 |  |  | |
| rs2908297 | 7 | 44193404 | T | -0.01323 | 0.002291 | 7.85E-09 | 332021 | 0.154685 | height_10 |  |  | |
| rs3823974 | 7 | 20442796 | C | -0.0182 | 0.001689 | 4.72E-27 | 332021 | 0.414436 | height_10 |  |  | |
| rs42036 | 7 | 92241451 | G | 0.031334 | 0.001927 | 2.11E-59 | 332021 | 0.246638 | height_10 |  |  | |
| rs78743535 | 7 | 46304421 | T | -0.03132 | 0.005044 | 5.34E-10 | 332021 | 0.0277729 | height_10 |  |  | |
| rs17157112 | 7 | 28779946 | G | -0.00921 | 0.001669 | 3.41E-08 | 332021 | 0.470735 | height_10 |  |  | |
| rs6967633 | 7 | 148393522 | T | -0.01224 | 0.002056 | 2.64E-09 | 332021 | 0.21244 | height_10 |  |  | |
| rs822530 | 7 | 148631555 | T | 0.023692 | 0.002066 | 1.93E-30 | 332021 | 0.794451 | height_10 |  |  | |
| rs11770186 | 7 | 25894232 | A | 0.014679 | 0.002092 | 2.29E-12 | 332021 | 0.195988 | height_10 |  |  | |
| rs7801581 | 7 | 27223771 | T | 0.01107 | 0.001937 | 1.09E-08 | 332021 | 0.252851 | height_10 |  |  | |
| rs10215458 | 7 | 120814211 | T | 0.010261 | 0.00166 | 6.30E-10 | 332021 | 0.508448 | height_10 |  |  | |
| rs2070744 | 7 | 150690079 | T | 0.01398 | 0.001715 | 3.58E-16 | 332021 | 0.612628 | height_10 |  |  | |
| rs6975015 | 7 | 50617215 | A | 0.014261 | 0.002586 | 3.48E-08 | 332021 | 0.117251 | height_10 |  |  | |
| rs723149 | 7 | 46577056 | G | -0.01455 | 0.001671 | 3.16E-18 | 332021 | 0.563721 | height_10 |  |  | |
| rs28872634 | 7 | 49607477 | T | 0.009968 | 0.001696 | 4.17E-09 | 332021 | 0.392682 | height_10 |  |  | |
| rs34743807 | 7 | 92566685 | G | -0.02121 | 0.002587 | 2.38E-16 | 332021 | 0.118525 | height_10 |  |  | |
| rs2715093 | 7 | 50733034 | C | -0.01116 | 0.001666 | 2.07E-11 | 332021 | 0.509205 | height_10 |  |  | |
| rs42528 | 7 | 94049356 | C | -0.01059 | 0.001926 | 3.76E-08 | 332021 | 0.752849 | height_10 |  |  | |
| rs140176556 | 7 | 92672844 | A | 0.031264 | 0.005383 | 6.35E-09 | 332021 | 0.0262723 | height_10 |  |  | |
| rs58063923 | 7 | 1270738 | T | -0.01891 | 0.00303 | 4.41E-10 | 332021 | 0.0815305 | height_10 |  |  | |
| rs2528539 | 7 | 93248951 | A | 0.009409 | 0.001668 | 1.70E-08 | 332021 | 0.440179 | height_10 |  |  | |
| rs7779665 | 7 | 23520963 | T | -0.01941 | 0.001683 | 9.03E-31 | 332021 | 0.418842 | height_10 |  |  | |
| rs17568389 | 7 | 8091876 | A | 0.009683 | 0.001662 | 5.65E-09 | 332021 | 0.512977 | height_10 |  |  | |
| rs12672756 | 7 | 46212931 | T | 0.01868 | 0.002263 | 1.51E-16 | 332021 | 0.166221 | height_10 |  |  | |
| rs2685148 | 7 | 37999558 | T | -0.0288 | 0.004172 | 5.14E-12 | 332021 | 0.0426618 | height_10 |  |  | |
| rs10104443 | 8 | 75976170 | T | -0.01377 | 0.001946 | 1.46E-12 | 332021 | 0.238223 | height_10 |  |  | |
| rs4735766 | 8 | 78099782 | T | 0.03329 | 0.001835 | 1.70E-73 | 332021 | 0.285453 | height_10 |  |  | |
| rs76193355 | 8 | 78917112 | T | 0.02166 | 0.00331 | 6.04E-11 | 332021 | 0.0705281 | height_10 |  |  | |
| rs2248185 | 8 | 25264962 | G | 0.009949 | 0.00173 | 8.89E-09 | 332021 | 0.63565 | height_10 |  |  | |
| rs1960661 | 8 | 131202241 | A | 0.012678 | 0.002083 | 1.15E-09 | 332021 | 0.198205 | height_10 |  |  | |
| rs16939126 | 8 | 76572453 | C | 0.0129 | 0.002268 | 1.28E-08 | 332021 | 0.160253 | height_10 |  |  | |
| rs76364830 | 8 | 13372120 | A | -0.02694 | 0.003455 | 6.43E-15 | 332021 | 0.0627567 | height_10 |  |  | |
| rs7014106 | 8 | 49386030 | T | 0.012353 | 0.001936 | 1.75E-10 | 332021 | 0.241362 | height_10 |  |  | |
| rs73228208 | 8 | 23381769 | A | 0.011076 | 0.001993 | 2.74E-08 | 332021 | 0.226138 | height_10 |  |  | |
| rs77249084 | 8 | 117572538 | G | 0.011346 | 0.001867 | 1.23E-09 | 332021 | 0.273828 | height_10 |  |  | |
| rs62515433 | 8 | 57129291 | T | 0.02462 | 0.002001 | 8.88E-35 | 332021 | 0.22241 | height_10 |  |  | |
| rs6998237 | 8 | 135584019 | T | -0.00982 | 0.001661 | 3.44E-09 | 332021 | 0.520392 | height_10 |  |  | |
| rs4870941 | 8 | 126498828 | C | -0.01813 | 0.001968 | 3.25E-20 | 332021 | 0.238653 | height_10 |  |  | |
| rs61729527 | 8 | 77761919 | T | -0.02158 | 0.003767 | 1.01E-08 | 332021 | 0.0511612 | height_10 |  |  | |
| rs72656010 | 8 | 57122215 | C | -0.03533 | 0.002465 | 1.38E-46 | 332021 | 0.130834 | height_10 |  |  | |
| rs12541381 | 8 | 135649848 | A | -0.01392 | 0.001895 | 2.08E-13 | 332021 | 0.256863 | height_10 |  |  | |
| rs2142331 | 8 | 116636719 | T | -0.00941 | 0.001695 | 2.88E-08 | 332021 | 0.601425 | height_10 |  |  | |
| rs4311660 | 8 | 23166656 | A | -0.01482 | 0.001858 | 1.52E-15 | 332021 | 0.723419 | height_10 |  |  | |
| rs2833 | 8 | 123986649 | G | 0.014281 | 0.001672 | 1.32E-17 | 332021 | 0.442097 | height_10 |  |  | |
| rs72722756 | 8 | 129186110 | C | 0.014478 | 0.002175 | 2.79E-11 | 332021 | 0.177512 | height_10 |  |  | |
| rs10808583 | 8 | 130718020 | A | -0.02866 | 0.002067 | 1.04E-43 | 332021 | 0.202216 | height_10 |  |  | |
| rs62501195 | 8 | 24041988 | C | -0.01933 | 0.002232 | 4.71E-18 | 332021 | 0.171613 | height_10 |  |  | |
| rs11784417 | 8 | 145010752 | T | -0.01598 | 0.001693 | 3.73E-21 | 332021 | 0.397783 | height_10 |  |  | |
| rs4398863 | 8 | 135695110 | C | -0.0107 | 0.00188 | 1.26E-08 | 332021 | 0.73585 | height_10 |  |  | |
| rs11135933 | 8 | 26162364 | G | 0.009333 | 0.001697 | 3.83E-08 | 332021 | 0.601849 | height_10 |  |  | |
| rs1599473 | 8 | 120475358 | T | -0.01478 | 0.001938 | 2.44E-14 | 332021 | 0.243251 | height_10 |  |  | |
| rs9650469 | 8 | 144837330 | C | 0.010207 | 0.001694 | 1.70E-09 | 332021 | 0.595963 | height_10 |  |  | |
| rs10756792 | 9 | 16726119 | T | 0.012201 | 0.001909 | 1.64E-10 | 332021 | 0.744728 | height_10 |  |  | |
| rs66779473 | 9 | 27136175 | A | -0.00962 | 0.001666 | 7.77E-09 | 332021 | 0.450419 | height_10 |  |  | |
| rs954585 | 9 | 109642696 | G | 0.015296 | 0.001737 | 1.29E-18 | 332021 | 0.356505 | height_10 |  |  | |
| rs1147369 | 9 | 132235522 | G | -0.00945 | 0.001658 | 1.21E-08 | 332021 | 0.48372 | height_10 |  |  | |
| rs912260 | 9 | 95493279 | G | -0.01146 | 0.001672 | 7.05E-12 | 332021 | 0.561963 | height_10 |  |  | |
| rs12378054 | 9 | 118411240 | C | -0.01893 | 0.003036 | 4.50E-10 | 332021 | 0.0822363 | height_10 |  |  | |
| rs1984119 | 9 | 98368761 | C | -0.02336 | 0.001917 | 3.78E-34 | 332021 | 0.257355 | height_10 |  |  | |
| rs10746837 | 9 | 90873653 | A | -0.01254 | 0.001689 | 1.17E-13 | 332021 | 0.581348 | height_10 |  |  | |
| rs72743489 | 9 | 96168483 | A | 0.010042 | 0.001694 | 3.05E-09 | 332021 | 0.593208 | height_10 |  |  | |
| rs10993460 | 9 | 97795176 | C | -0.01807 | 0.002914 | 5.63E-10 | 332021 | 0.0886361 | height_10 |  |  | |
| rs2236406 | 9 | 98221861 | C | 0.014688 | 0.001742 | 3.37E-17 | 332021 | 0.349285 | height_10 |  |  | |
| rs12554172 | 9 | 100797597 | A | -0.0128 | 0.002008 | 1.86E-10 | 332021 | 0.220014 | height_10 |  |  | |
| rs3802342 | 9 | 133473303 | G | -0.01554 | 0.001735 | 3.41E-19 | 332021 | 0.353294 | height_10 |  |  | |
| rs2274114 | 9 | 139091460 | A | 0.019957 | 0.002123 | 5.51E-21 | 332021 | 0.18983 | height_10 |  |  | |
| rs112371891 | 9 | 96937049 | G | 0.039393 | 0.004674 | 3.50E-17 | 332021 | 0.033458 | height_10 |  |  | |
| rs10870071 | 9 | 139382184 | C | 0.011504 | 0.001666 | 4.98E-12 | 332021 | 0.446294 | height_10 |  |  | |
| rs4878043 | 9 | 89687854 | T | 0.010284 | 0.001672 | 7.77E-10 | 332021 | 0.543381 | height_10 |  |  | |
| rs6479363 | 9 | 94335200 | C | 0.010985 | 0.001848 | 2.78E-09 | 332021 | 0.284227 | height_10 |  |  | |
| rs10975978 | 9 | 7018817 | G | -0.01676 | 0.002696 | 5.17E-10 | 332021 | 0.106734 | height_10 |  |  | |
| rs4647519 | 9 | 97886456 | A | 0.021193 | 0.003753 | 1.64E-08 | 332021 | 0.054327 | height_10 |  |  | |
| rs10982576 | 9 | 117920248 | C | 0.010788 | 0.001946 | 2.95E-08 | 332021 | 0.240604 | height_10 |  |  | |
| rs902144 | 9 | 109177571 | C | 0.010858 | 0.00166 | 6.12E-11 | 332021 | 0.481044 | height_10 |  |  | |
| rs62581708 | 9 | 123603899 | C | 0.010914 | 0.001924 | 1.42E-08 | 332021 | 0.248469 | height_10 |  |  | |
| rs7047000 | 9 | 98426006 | C | 0.009078 | 0.001663 | 4.84E-08 | 332021 | 0.469571 | height_10 |  |  | |
| rs751543 | 9 | 119122342 | T | 0.016495 | 0.001825 | 1.63E-19 | 332021 | 0.710313 | height_10 |  |  | |
| rs17302434 | 9 | 119155732 | C | 0.017131 | 0.002757 | 5.20E-10 | 332021 | 0.100441 | height_10 |  |  | |
| rs7870253 | 9 | 99231096 | A | 0.016776 | 0.002006 | 6.15E-17 | 332021 | 0.219462 | height_10 |  |  | |
| rs958225 | 9 | 78759705 | A | 0.020985 | 0.003605 | 5.83E-09 | 332021 | 0.0569219 | height_10 |  |  | |
| rs13297831 | 9 | 35911833 | G | -0.01023 | 0.001696 | 1.61E-09 | 332021 | 0.430139 | height_10 |  |  | |
| rs35307904 | 9 | 78511889 | A | -0.02951 | 0.002545 | 4.41E-31 | 332021 | 0.122158 | height_10 |  |  | |
| rs9410916 | 9 | 89089476 | T | 0.011471 | 0.001658 | 4.61E-12 | 332021 | 0.502516 | height_10 |  |  | |
| rs2902406 | 10 | 102660281 | C | 0.018569 | 0.001675 | 1.43E-28 | 332021 | 0.432263 | height_10 |  |  | |
| rs61849823 | 10 | 53290991 | C | 0.018776 | 0.002257 | 8.91E-17 | 332021 | 0.163793 | height_10 |  |  | |
| rs72841270 | 10 | 104642237 | G | 0.018872 | 0.00243 | 8.04E-15 | 332021 | 0.134388 | height_10 |  |  | |
| rs7079567 | 10 | 23742383 | A | 0.011343 | 0.001843 | 7.49E-10 | 332021 | 0.28286 | height_10 |  |  | |
| rs11198898 | 10 | 121118492 | C | -0.01318 | 0.001955 | 1.58E-11 | 332021 | 0.76358 | height_10 |  |  | |
| rs12411442 | 10 | 104349531 | G | -0.01417 | 0.001676 | 2.79E-17 | 332021 | 0.429866 | height_10 |  |  | |
| rs117543413 | 10 | 79543740 | T | -0.03832 | 0.006339 | 1.49E-09 | 332021 | 0.0178841 | height_10 |  |  | |
| rs79043147 | 10 | 124233181 | T | -0.02422 | 0.003205 | 4.14E-14 | 332021 | 0.072911 | height_10 |  |  | |
| rs779933 | 10 | 80918517 | A | -0.01344 | 0.001676 | 1.07E-15 | 332021 | 0.433296 | height_10 |  |  | |
| rs1245564 | 10 | 73836867 | T | -0.01077 | 0.001664 | 9.96E-11 | 332021 | 0.502229 | height_10 |  |  | |
| rs2339686 | 10 | 52766038 | A | -0.01061 | 0.001893 | 2.09E-08 | 332021 | 0.261092 | height_10 |  |  | |
| rs10761494 | 10 | 62160833 | T | 0.010195 | 0.001727 | 3.57E-09 | 332021 | 0.637125 | height_10 |  |  | |
| rs10740055 | 10 | 63718479 | A | -0.0092 | 0.001661 | 3.03E-08 | 332021 | 0.490758 | height_10 |  |  | |
| rs7916821 | 10 | 69933969 | A | 0.012837 | 0.001658 | 9.80E-15 | 332021 | 0.497337 | height_10 |  |  | |
| rs35954730 | 10 | 12943111 | A | -0.01543 | 0.001836 | 4.29E-17 | 332021 | 0.288871 | height_10 |  |  | |
| rs10998648 | 10 | 70977395 | C | 0.010625 | 0.001798 | 3.43E-09 | 332021 | 0.691009 | height_10 |  |  | |
| rs4980067 | 10 | 81136129 | A | -0.01424 | 0.001659 | 9.21E-18 | 332021 | 0.50449 | height_10 |  |  | |
| rs2631676 | 10 | 93037409 | G | 0.012134 | 0.002111 | 9.00E-09 | 332021 | 0.190337 | height_10 |  |  | |
| rs10829605 | 10 | 131344413 | T | 0.012135 | 0.001701 | 9.84E-13 | 332021 | 0.613314 | height_10 |  |  | |
| rs11245343 | 10 | 126415795 | C | 0.011627 | 0.001802 | 1.09E-10 | 332021 | 0.310646 | height_10 |  |  | |
| rs12293395 | 11 | 17047661 | G | 0.012102 | 0.001874 | 1.06E-10 | 332021 | 0.271333 | height_10 |  |  | |
| rs606452 | 11 | 75276178 | C | -0.02248 | 0.002388 | 4.84E-21 | 332021 | 0.860178 | height_10 |  |  | |
| rs117447625 | 11 | 71958986 | T | -0.05418 | 0.009204 | 3.94E-09 | 332021 | 0.00821177 | height_10 |  |  | |
| rs11042355 | 11 | 9465011 | A | 0.011129 | 0.001678 | 3.30E-11 | 332021 | 0.426969 | height_10 |  |  | |
| rs12788184 | 11 | 28389866 | G | -0.01287 | 0.001668 | 1.23E-14 | 332021 | 0.457234 | height_10 |  |  | |
| rs7120438 | 11 | 69902227 | T | 0.010203 | 0.00181 | 1.72E-08 | 332021 | 0.694126 | height_10 |  |  | |
| rs9787897 | 11 | 74659302 | T | 0.010098 | 0.001844 | 4.36E-08 | 332021 | 0.279906 | height_10 |  |  | |
| rs113875379 | 11 | 69279570 | C | 0.025999 | 0.004592 | 1.50E-08 | 332021 | 0.035227 | height_10 |  |  | |
| rs654169 | 11 | 128691920 | A | -0.01219 | 0.001846 | 4.08E-11 | 332021 | 0.292245 | height_10 |  |  | |
| rs7936836 | 11 | 43633645 | A | -0.01165 | 0.001685 | 4.77E-12 | 332021 | 0.419303 | height_10 |  |  | |
| rs10838681 | 11 | 47275064 | A | 0.013244 | 0.001869 | 1.37E-12 | 332021 | 0.268333 | height_10 |  |  | |
| rs77735964 | 11 | 2075720 | G | -0.01327 | 0.002073 | 1.55E-10 | 332021 | 0.201421 | height_10 |  |  | |
| rs10832963 | 11 | 18664241 | G | -0.01056 | 0.001909 | 3.12E-08 | 332021 | 0.745867 | height_10 |  |  | |
| rs2237886 | 11 | 2810731 | T | 0.02376 | 0.002707 | 1.71E-18 | 332021 | 0.10453 | height_10 |  |  | |
| rs35506085 | 11 | 2165576 | A | -0.01585 | 0.002167 | 2.57E-13 | 332021 | 0.181583 | height_10 |  |  | |
| rs511987 | 11 | 125981569 | C | -0.00989 | 0.001745 | 1.45E-08 | 332021 | 0.371392 | height_10 |  |  | |
| rs3851111 | 11 | 126083218 | T | -0.0111 | 0.001794 | 6.14E-10 | 332021 | 0.308247 | height_10 |  |  | |
| rs143840904 | 11 | 2813322 | T | -0.07389 | 0.006225 | 1.73E-32 | 332021 | 0.0192646 | height_10 |  |  | |
| rs10765993 | 11 | 12849185 | C | 0.01277 | 0.001682 | 3.14E-14 | 332021 | 0.423011 | height_10 |  |  | |
| rs1878532 | 11 | 28852980 | A | 0.013594 | 0.002276 | 2.32E-09 | 332021 | 0.842933 | height_10 |  |  | |
| rs648101 | 11 | 64998593 | C | 0.010994 | 0.001824 | 1.67E-09 | 332021 | 0.293657 | height_10 |  |  | |
| rs2513281 | 11 | 68417094 | G | 0.016514 | 0.002305 | 7.81E-13 | 332021 | 0.844393 | height_10 |  |  | |
| rs7115854 | 11 | 30311493 | G | -0.01029 | 0.001862 | 3.30E-08 | 332021 | 0.271531 | height_10 |  |  | |
| rs78108235 | 11 | 89245403 | A | 0.017362 | 0.003048 | 1.22E-08 | 332021 | 0.0831897 | height_10 |  |  | |
| rs56088284 | 11 | 66899619 | G | -0.05258 | 0.00299 | 3.69E-69 | 332021 | 0.083986 | height_10 |  |  | |
| rs77216358 | 11 | 120311157 | G | -0.02954 | 0.003973 | 1.04E-13 | 332021 | 0.0465422 | height_10 |  |  | |
| rs10893812 | 11 | 127932610 | C | -0.01438 | 0.002156 | 2.58E-11 | 332021 | 0.185583 | height_10 |  |  | |
| rs11607789 | 11 | 85526151 | T | -0.01113 | 0.001893 | 4.10E-09 | 332021 | 0.261842 | height_10 |  |  | |
| rs11600815 | 11 | 14797227 | A | 0.028444 | 0.003802 | 7.33E-14 | 332021 | 0.0536908 | height_10 |  |  | |
| rs11049576 | 12 | 28565280 | C | -0.02212 | 0.001817 | 4.10E-34 | 332021 | 0.295587 | height_10 |  |  | |
| rs2900208 | 12 | 11878464 | A | 0.02324 | 0.001735 | 6.37E-41 | 332021 | 0.355387 | height_10 |  |  | |
| rs7311238 | 12 | 54020492 | A | -0.01192 | 0.002154 | 3.10E-08 | 332021 | 0.180732 | height_10 |  |  | |
| rs79345295 | 12 | 102942043 | T | -0.063 | 0.008775 | 7.01E-13 | 332021 | 0.00907031 | height_10 |  |  | |
| rs7977765 | 12 | 107205390 | G | -0.01281 | 0.001944 | 4.39E-11 | 332021 | 0.238184 | height_10 |  |  | |
| rs6581627 | 12 | 65719229 | C | -0.00936 | 0.001661 | 1.78E-08 | 332021 | 0.522088 | height_10 |  |  | |
| rs11613576 | 12 | 104392497 | T | 0.016313 | 0.002919 | 2.30E-08 | 332021 | 0.0887117 | height_10 |  |  | |
| rs11062011 | 12 | 329131 | C | -0.00929 | 0.001695 | 4.20E-08 | 332021 | 0.459572 | height_10 |  |  | |
| rs310792 | 12 | 77450413 | T | 0.014352 | 0.001826 | 3.82E-15 | 332021 | 0.702091 | height_10 |  |  | |
| rs76895963 | 12 | 4384844 | G | 0.083973 | 0.006418 | 4.12E-39 | 332021 | 0.0192794 | height_10 |  |  | |
| rs215226 | 12 | 591300 | G | 0.013859 | 0.001698 | 3.27E-16 | 332021 | 0.402219 | height_10 |  |  | |
| rs6487088 | 12 | 20588382 | T | 0.016596 | 0.002114 | 4.13E-15 | 332021 | 0.800516 | height_10 |  |  | |
| rs76892561 | 12 | 111500681 | G | 0.026742 | 0.003996 | 2.20E-11 | 332021 | 0.0458112 | height_10 |  |  | |
| rs76929617 | 12 | 120867798 | G | -0.03416 | 0.004292 | 1.72E-15 | 332021 | 0.0388598 | height_10 |  |  | |
| rs10748128 | 12 | 69827658 | T | 0.015979 | 0.001744 | 5.19E-20 | 332021 | 0.344848 | height_10 |  |  | |
| rs2364232 | 12 | 93994827 | C | 0.020872 | 0.001898 | 4.00E-28 | 332021 | 0.259359 | height_10 |  |  | |
| rs1351394 | 12 | 66351826 | C | -0.02954 | 0.00166 | 7.86E-71 | 332021 | 0.509586 | height_10 |  |  | |
| rs12319783 | 12 | 102322942 | T | 0.015446 | 0.002809 | 3.81E-08 | 332021 | 0.096919 | height_10 |  |  | |
| rs117081218 | 12 | 102339359 | A | -0.03286 | 0.00456 | 5.74E-13 | 332021 | 0.0366772 | height_10 |  |  | |
| rs855203 | 12 | 102958073 | A | -0.0167 | 0.002779 | 1.85E-09 | 332021 | 0.899948 | height_10 |  |  | |
| rs79747671 | 12 | 103140344 | T | -0.01681 | 0.002665 | 2.80E-10 | 332021 | 0.108547 | height_10 |  |  | |
| rs12321509 | 12 | 24222096 | A | 0.01281 | 0.001792 | 8.91E-13 | 332021 | 0.307191 | height_10 |  |  | |
| rs1035606 | 12 | 29509450 | T | 0.013536 | 0.001843 | 2.06E-13 | 332021 | 0.283662 | height_10 |  |  | |
| rs10777560 | 12 | 94326641 | C | -0.01091 | 0.001686 | 9.68E-11 | 332021 | 0.405544 | height_10 |  |  | |
| rs7132908 | 12 | 50263148 | A | 0.010826 | 0.001706 | 2.21E-10 | 332021 | 0.38392 | height_10 |  |  | |
| rs10770699 | 12 | 20832321 | G | -0.01134 | 0.001684 | 1.65E-11 | 332021 | 0.584118 | height_10 |  |  | |
| rs7310615 | 12 | 111865049 | G | 0.012639 | 0.001671 | 3.86E-14 | 332021 | 0.516763 | height_10 |  |  | |
| rs4567503 | 12 | 122736492 | C | 0.012759 | 0.001753 | 3.39E-13 | 332021 | 0.340333 | height_10 |  |  | |
| rs2710278 | 12 | 12045185 | C | -0.01061 | 0.001811 | 4.62E-09 | 332021 | 0.301372 | height_10 |  |  | |
| rs2058789 | 12 | 14908319 | T | -0.0098 | 0.001718 | 1.16E-08 | 332021 | 0.376491 | height_10 |  |  | |
| rs12824685 | 12 | 123817569 | T | 0.021811 | 0.002072 | 6.48E-26 | 332021 | 0.203341 | height_10 |  |  | |
| rs7976110 | 12 | 124249818 | A | 0.010152 | 0.001815 | 2.21E-08 | 332021 | 0.310515 | height_10 |  |  | |
| rs58730238 | 12 | 66018252 | T | -0.04639 | 0.006928 | 2.15E-11 | 332021 | 0.01492 | height_10 |  |  | |
| rs10778161 | 12 | 102367384 | G | 0.015243 | 0.001843 | 1.36E-16 | 332021 | 0.282963 | height_10 |  |  | |
| rs10879013 | 12 | 70146120 | C | -0.01263 | 0.00223 | 1.47E-08 | 332021 | 0.165706 | height_10 |  |  | |
| rs3858660 | 12 | 97580415 | C | -0.01531 | 0.002637 | 6.43E-09 | 332021 | 0.112404 | height_10 |  |  | |
| rs17426844 | 12 | 116632928 | T | -0.01688 | 0.002366 | 9.73E-13 | 332021 | 0.144415 | height_10 |  |  | |
| rs7970695 | 12 | 121423376 | A | -0.00993 | 0.001713 | 6.65E-09 | 332021 | 0.622865 | height_10 |  |  | |
| rs1263993 | 12 | 124828059 | T | -0.01493 | 0.001704 | 1.98E-18 | 332021 | 0.603749 | height_10 |  |  | |
| rs7953280 | 12 | 94136009 | C | -0.01682 | 0.001666 | 5.85E-24 | 332021 | 0.507457 | height_10 |  |  | |
| rs2277339 | 12 | 57146069 | G | -0.01623 | 0.002727 | 2.65E-09 | 332021 | 0.103178 | height_10 |  |  | |
| rs9523312 | 13 | 92013944 | A | -0.01595 | 0.001695 | 4.99E-21 | 332021 | 0.604197 | height_10 |  |  | |
| rs198607 | 13 | 48979167 | C | 0.010317 | 0.001873 | 3.62E-08 | 332021 | 0.26795 | height_10 |  |  | |
| rs7326823 | 13 | 21543685 | A | -0.01554 | 0.002656 | 4.88E-09 | 332021 | 0.113464 | height_10 |  |  | |
| rs113262473 | 13 | 50420391 | A | -0.01162 | 0.002026 | 9.59E-09 | 332021 | 0.216725 | height_10 |  |  | |
| rs3118906 | 13 | 51106788 | A | -0.02173 | 0.001848 | 6.40E-32 | 332021 | 0.277922 | height_10 |  |  | |
| rs77929895 | 13 | 50072429 | C | -0.01755 | 0.002924 | 1.94E-09 | 332021 | 0.0882446 | height_10 |  |  | |
| rs3993426 | 13 | 115075317 | C | -0.01489 | 0.002057 | 4.63E-13 | 332021 | 0.207715 | height_10 |  |  | |
| rs1822365 | 13 | 21406858 | G | -0.01178 | 0.002141 | 3.73E-08 | 332021 | 0.81647 | height_10 |  |  | |
| rs9595885 | 13 | 33168050 | A | 0.010987 | 0.001721 | 1.73E-10 | 332021 | 0.371269 | height_10 |  |  | |
| rs9315784 | 13 | 41289166 | A | -0.01225 | 0.001831 | 2.26E-11 | 332021 | 0.294526 | height_10 |  |  | |
| rs2812208 | 13 | 50707087 | C | 0.065808 | 0.005729 | 1.57E-30 | 332021 | 0.0213761 | height_10 |  |  | |
| rs9533031 | 13 | 42772717 | T | 0.012005 | 0.001678 | 8.29E-13 | 332021 | 0.578768 | height_10 |  |  | |
| rs17256211 | 14 | 23754580 | G | 0.011353 | 0.001746 | 7.99E-11 | 332021 | 0.650092 | height_10 |  |  | |
| rs7156335 | 14 | 93406232 | C | 0.017146 | 0.002917 | 4.13E-09 | 332021 | 0.0894487 | height_10 |  |  | |
| rs75741145 | 14 | 101202816 | A | -0.01648 | 0.002602 | 2.38E-10 | 332021 | 0.114938 | height_10 |  |  | |
| rs10141617 | 14 | 70416442 | A | 0.011684 | 0.001762 | 3.35E-11 | 332021 | 0.334633 | height_10 |  |  | |
| rs3784004 | 14 | 76081664 | T | -0.01083 | 0.001884 | 8.97E-09 | 332021 | 0.73517 | height_10 |  |  | |
| rs4899012 | 14 | 61003889 | C | -0.01501 | 0.001701 | 1.13E-18 | 332021 | 0.61037 | height_10 |  |  | |
| rs3181251 | 14 | 24805824 | T | 0.017057 | 0.001886 | 1.49E-19 | 332021 | 0.2615 | height_10 |  |  | |
| rs17880989 | 14 | 23313633 | A | 0.031538 | 0.005262 | 2.05E-09 | 332021 | 0.0255191 | height_10 |  |  | |
| rs862041 | 14 | 74986403 | C | 0.017148 | 0.001727 | 3.08E-23 | 332021 | 0.637235 | height_10 |  |  | |
| rs61980882 | 14 | 75021464 | T | -0.01067 | 0.001668 | 1.63E-10 | 332021 | 0.500356 | height_10 |  |  | |
| rs10151561 | 14 | 92430184 | G | -0.0149 | 0.001685 | 9.19E-19 | 332021 | 0.422541 | height_10 |  |  | |
| rs28929474 | 14 | 94844947 | T | 0.059893 | 0.005855 | 1.48E-24 | 332021 | 0.0204301 | height_10 |  |  | |
| rs10131337 | 14 | 37144516 | T | 0.011643 | 0.001936 | 1.81E-09 | 332021 | 0.245173 | height_10 |  |  | |
| rs17197100 | 14 | 21837879 | G | 0.015515 | 0.002495 | 5.00E-10 | 332021 | 0.127632 | height_10 |  |  | |
| rs10459573 | 14 | 103925457 | C | -0.01467 | 0.001715 | 1.24E-17 | 332021 | 0.370578 | height_10 |  |  | |
| rs8019890 | 14 | 21538067 | A | 0.012835 | 0.001685 | 2.58E-14 | 332021 | 0.53047 | height_10 |  |  | |
| rs2498851 | 14 | 92332548 | A | 0.015869 | 0.002212 | 7.30E-13 | 332021 | 0.169553 | height_10 |  |  | |
| rs7146939 | 14 | 65583581 | C | 0.014518 | 0.001683 | 6.26E-18 | 332021 | 0.580458 | height_10 |  |  | |
| rs2332326 | 14 | 24835859 | C | -0.01681 | 0.002665 | 2.82E-10 | 332021 | 0.891101 | height_10 |  |  | |
| rs74630865 | 15 | 84259565 | G | -0.01178 | 0.001909 | 6.78E-10 | 332021 | 0.262316 | height_10 |  |  | |
| rs12907384 | 15 | 86276000 | C | -0.01115 | 0.001663 | 2.03E-11 | 332021 | 0.535823 | height_10 |  |  | |
| rs17205463 | 15 | 62381413 | T | -0.01351 | 0.00167 | 6.02E-16 | 332021 | 0.449459 | height_10 |  |  | |
| rs2663126 | 15 | 99563857 | A | -0.01168 | 0.001792 | 7.15E-11 | 332021 | 0.69014 | height_10 |  |  | |
| rs2165241 | 15 | 74222202 | C | 0.020238 | 0.00166 | 3.35E-34 | 332021 | 0.507746 | height_10 |  |  | |
| rs12906942 | 15 | 72794405 | C | 0.040883 | 0.006103 | 2.10E-11 | 332021 | 0.0187931 | height_10 |  |  | |
| rs10906982 | 15 | 84568158 | A | 0.027569 | 0.001659 | 5.13E-62 | 332021 | 0.521223 | height_10 |  |  | |
| rs4932200 | 15 | 89349708 | G | 0.014421 | 0.001663 | 4.37E-18 | 332021 | 0.48352 | height_10 |  |  | |
| rs12908947 | 15 | 89385632 | G | -0.01918 | 0.00186 | 6.11E-25 | 332021 | 0.72576 | height_10 |  |  | |
| rs28583508 | 15 | 100800049 | T | -0.01967 | 0.001762 | 6.02E-29 | 332021 | 0.660847 | height_10 |  |  | |
| rs2871865 | 15 | 99194896 | G | -0.02688 | 0.002594 | 3.59E-25 | 332021 | 0.115645 | height_10 |  |  | |
| rs920141 | 15 | 40234813 | C | -0.01106 | 0.001951 | 1.45E-08 | 332021 | 0.237587 | height_10 |  |  | |
| rs685091 | 15 | 48848130 | C | -0.0252 | 0.002816 | 3.62E-19 | 332021 | 0.096188 | height_10 |  |  | |
| rs35874463 | 15 | 67457698 | G | 0.050039 | 0.003547 | 3.50E-45 | 332021 | 0.057733 | height_10 |  |  | |
| rs2415130 | 15 | 72242554 | A | 0.011269 | 0.001955 | 8.24E-09 | 332021 | 0.235804 | height_10 |  |  | |
| rs56173559 | 15 | 67008443 | G | 0.010978 | 0.00188 | 5.25E-09 | 332021 | 0.271528 | height_10 |  |  | |
| rs11632935 | 15 | 89439215 | T | -0.01314 | 0.001678 | 4.86E-15 | 332021 | 0.433738 | height_10 |  |  | |
| rs8039718 | 15 | 70356150 | T | 0.016431 | 0.002167 | 3.36E-14 | 332021 | 0.179093 | height_10 |  |  | |
| rs10152898 | 15 | 74255121 | T | -0.01893 | 0.00217 | 2.71E-18 | 332021 | 0.178294 | height_10 |  |  | |
| rs2074585 | 15 | 91009484 | A | 0.011541 | 0.001657 | 3.26E-12 | 332021 | 0.512556 | height_10 |  |  | |
| rs2289790 | 15 | 67476970 | C | -0.01132 | 0.00195 | 6.46E-09 | 332021 | 0.24079 | height_10 |  |  | |
| rs1529889 | 15 | 100539258 | A | 0.011345 | 0.001691 | 1.95E-11 | 332021 | 0.457604 | height_10 |  |  | |
| rs62621400 | 15 | 101718239 | G | -0.02134 | 0.00359 | 2.78E-09 | 332021 | 0.0567143 | height_10 |  |  | |
| rs2466949 | 15 | 66086188 | G | 0.01366 | 0.002163 | 2.69E-10 | 332021 | 0.179452 | height_10 |  |  | |
| rs72765638 | 15 | 89257734 | C | -0.01583 | 0.002539 | 4.52E-10 | 332021 | 0.121305 | height_10 |  |  | |
| rs190543502 | 15 | 43757184 | C | -0.0331 | 0.005519 | 2.00E-09 | 332021 | 0.023753 | height_10 |  |  | |
| rs1351438 | 15 | 100601469 | C | -0.01152 | 0.002091 | 3.60E-08 | 332021 | 0.800514 | height_10 |  |  | |
| rs72755233 | 15 | 100692953 | A | -0.04654 | 0.002616 | 8.66E-71 | 332021 | 0.112887 | height_10 |  |  | |
| rs28584580 | 15 | 89397827 | G | -0.08015 | 0.004897 | 3.52E-60 | 332021 | 0.0294692 | height_10 |  |  | |
| rs8031196 | 15 | 80994931 | T | 0.009981 | 0.001668 | 2.20E-09 | 332021 | 0.448959 | height_10 |  |  | |
| rs782932 | 15 | 61409980 | A | -0.01359 | 0.001754 | 9.08E-15 | 332021 | 0.340682 | height_10 |  |  | |
| rs17200030 | 15 | 89379348 | T | -0.03625 | 0.004874 | 1.03E-13 | 332021 | 0.0298444 | height_10 |  |  | |
| rs34092297 | 15 | 70010472 | G | -0.02234 | 0.002743 | 3.80E-16 | 332021 | 0.101944 | height_10 |  |  | |
| rs36008565 | 16 | 84751046 | G | 0.01291 | 0.001936 | 2.62E-11 | 332021 | 0.248941 | height_10 |  |  | |
| rs2907272 | 16 | 4019350 | T | 0.015018 | 0.002169 | 4.43E-12 | 332021 | 0.82145 | height_10 |  |  | |
| rs12925127 | 16 | 24728185 | A | 0.009619 | 0.001756 | 4.31E-08 | 332021 | 0.336725 | height_10 |  |  | |
| rs112758960 | 16 | 67308279 | A | -0.02872 | 0.004102 | 2.53E-12 | 332021 | 0.0426632 | height_10 |  |  | |
| rs73532431 | 16 | 20939509 | A | -0.01289 | 0.002222 | 6.66E-09 | 332021 | 0.16706 | height_10 |  |  | |
| rs9940278 | 16 | 53800200 | T | 0.009465 | 0.001679 | 1.73E-08 | 332021 | 0.421924 | height_10 |  |  | |
| rs112549897 | 16 | 29832950 | G | -0.02157 | 0.003784 | 1.19E-08 | 332021 | 0.0540988 | height_10 |  |  | |
| rs35467921 | 16 | 30048553 | T | 0.013987 | 0.001691 | 1.31E-16 | 332021 | 0.401665 | height_10 |  |  | |
| rs7190096 | 16 | 9066756 | G | 0.01142 | 0.00193 | 3.26E-09 | 332021 | 0.246301 | height_10 |  |  | |
| rs76513770 | 16 | 72505534 | C | -0.02037 | 0.002474 | 1.85E-16 | 332021 | 0.12908 | height_10 |  |  | |
| rs2967363 | 16 | 82204132 | G | -0.01303 | 0.001974 | 4.15E-11 | 332021 | 0.770916 | height_10 |  |  | |
| rs753602 | 16 | 88807224 | C | 0.012335 | 0.001814 | 1.06E-11 | 332021 | 0.70197 | height_10 |  |  | |
| rs11648221 | 16 | 75182089 | C | 0.010527 | 0.001709 | 7.34E-10 | 332021 | 0.620362 | height_10 |  |  | |
| rs6600203 | 16 | 2445480 | G | 0.010264 | 0.001767 | 6.31E-09 | 332021 | 0.670675 | height_10 |  |  | |
| rs4074872 | 16 | 15117299 | A | -0.01219 | 0.002107 | 7.36E-09 | 332021 | 0.250317 | height_10 |  |  | |
| rs10514517 | 16 | 81579729 | T | 0.015318 | 0.002137 | 7.62E-13 | 332021 | 0.184877 | height_10 |  |  | |
| rs4303473 | 16 | 84901475 | C | 0.01163 | 0.001716 | 1.21E-11 | 332021 | 0.377483 | height_10 |  |  | |
| rs56186137 | 16 | 28825953 | G | 0.012096 | 0.001688 | 7.68E-13 | 332021 | 0.404359 | height_10 |  |  | |
| rs933561 | 16 | 49874676 | G | -0.01006 | 0.001742 | 7.63E-09 | 332021 | 0.347575 | height_10 |  |  | |
| rs13338700 | 16 | 781292 | T | 0.021781 | 0.002068 | 6.13E-26 | 332021 | 0.20197 | height_10 |  |  | |
| rs11639510 | 16 | 3485490 | C | 0.009957 | 0.001685 | 3.42E-09 | 332021 | 0.576019 | height_10 |  |  | |
| rs138937927 | 16 | 51095561 | T | -0.03541 | 0.005359 | 3.92E-11 | 332021 | 0.0263835 | height_10 |  |  | |
| rs12445470 | 16 | 997496 | A | 0.014587 | 0.001728 | 3.11E-17 | 332021 | 0.369595 | height_10 |  |  | |
| rs34978797 | 16 | 2144400 | G | -0.01961 | 0.003079 | 1.87E-10 | 332021 | 0.0790735 | height_10 |  |  | |
| rs27384 | 16 | 2248888 | C | 0.016699 | 0.001667 | 1.30E-23 | 332021 | 0.444898 | height_10 |  |  | |
| rs11540358 | 16 | 53503943 | G | 0.014961 | 0.001796 | 8.00E-17 | 332021 | 0.306307 | height_10 |  |  | |
| rs9935080 | 16 | 4902207 | G | 0.010904 | 0.001664 | 5.68E-11 | 332021 | 0.474422 | height_10 |  |  | |
| rs1126464 | 16 | 89704365 | C | 0.014273 | 0.001934 | 1.58E-13 | 332021 | 0.243401 | height_10 |  |  | |
| rs11861996 | 16 | 90105281 | G | -0.01427 | 0.002358 | 1.43E-09 | 332021 | 0.852912 | height_10 |  |  | |
| rs3020619 | 17 | 61993137 | G | 0.026143 | 0.001876 | 4.08E-44 | 332021 | 0.271528 | height_10 |  |  | |
| rs8072010 | 17 | 69930198 | C | 0.013133 | 0.001901 | 4.88E-12 | 332021 | 0.259428 | height_10 |  |  | |
| rs1114297 | 17 | 59599207 | C | -0.00972 | 0.001783 | 4.99E-08 | 332021 | 0.677189 | height_10 |  |  | |
| rs9890133 | 17 | 68169005 | G | -0.01576 | 0.002589 | 1.13E-09 | 332021 | 0.117269 | height_10 |  |  | |
| rs8074344 | 17 | 62020866 | G | 0.012631 | 0.001688 | 7.20E-14 | 332021 | 0.566938 | height_10 |  |  | |
| rs55749333 | 17 | 7371932 | T | -0.01022 | 0.001727 | 3.25E-09 | 332021 | 0.638267 | height_10 |  |  | |
| rs9747062 | 17 | 8023057 | C | 0.012647 | 0.0017 | 1.00E-13 | 332021 | 0.604584 | height_10 |  |  | |
| rs1619588 | 17 | 42063363 | A | -0.00975 | 0.001716 | 1.32E-08 | 332021 | 0.628794 | height_10 |  |  | |
| rs112058117 | 17 | 44041562 | T | -0.01196 | 0.002007 | 2.57E-09 | 332021 | 0.224357 | height_10 |  |  | |
| rs8073455 | 17 | 54849224 | T | -0.017 | 0.001659 | 1.30E-24 | 332021 | 0.493069 | height_10 |  |  | |
| rs6503750 | 17 | 54712139 | A | 0.024647 | 0.003807 | 9.58E-11 | 332021 | 0.94946 | height_10 |  |  | |
| rs11870991 | 17 | 42836124 | A | 0.014676 | 0.002389 | 8.16E-10 | 332021 | 0.140734 | height_10 |  |  | |
| rs9892365 | 17 | 59491384 | G | -0.01861 | 0.001763 | 4.67E-26 | 332021 | 0.669676 | height_10 |  |  | |
| rs9912553 | 17 | 79959703 | G | 0.013545 | 0.001854 | 2.75E-13 | 332021 | 0.723644 | height_10 |  |  | |
| rs9902386 | 17 | 21280185 | G | 0.011333 | 0.00179 | 2.45E-10 | 332021 | 0.677151 | height_10 |  |  | |
| rs8075255 | 17 | 46958166 | C | 0.020068 | 0.002185 | 4.20E-20 | 332021 | 0.175263 | height_10 |  |  | |
| rs4789193 | 17 | 73404796 | T | 0.0152 | 0.002248 | 1.36E-11 | 332021 | 0.837583 | height_10 |  |  | |
| rs12452590 | 17 | 60720058 | G | 0.01054 | 0.001743 | 1.49E-09 | 332021 | 0.363041 | height_10 |  |  | |
| rs7223643 | 17 | 65830282 | G | 0.011627 | 0.002119 | 4.08E-08 | 332021 | 0.189686 | height_10 |  |  | |
| rs12452505 | 17 | 63556402 | G | -0.01913 | 0.002394 | 1.33E-15 | 332021 | 0.140525 | height_10 |  |  | |
| rs11867479 | 17 | 68090207 | T | 0.010472 | 0.001729 | 1.39E-09 | 332021 | 0.3586 | height_10 |  |  | |
| rs2530802 | 17 | 54763443 | G | 0.018484 | 0.001859 | 2.73E-23 | 332021 | 0.273146 | height_10 |  |  | |
| rs584828 | 17 | 38599230 | T | -0.014 | 0.001694 | 1.41E-16 | 332021 | 0.400372 | height_10 |  |  | |
| rs652093 | 17 | 18269099 | G | -0.01364 | 0.001721 | 2.31E-15 | 332021 | 0.633068 | height_10 |  |  | |
| rs12450371 | 17 | 1667674 | T | 0.012574 | 0.001748 | 6.36E-13 | 332021 | 0.348305 | height_10 |  |  | |
| rs72823964 | 17 | 28120887 | T | 0.018967 | 0.002438 | 7.24E-15 | 332021 | 0.133002 | height_10 |  |  | |
| rs7223535 | 17 | 29211667 | A | -0.0281 | 0.001866 | 3.10E-51 | 332021 | 0.270191 | height_10 |  |  | |
| rs117223734 | 17 | 54694724 | G | 0.043691 | 0.00665 | 5.05E-11 | 332021 | 0.0157444 | height_10 |  |  | |
| rs1043515 | 17 | 36922196 | G | 0.01179 | 0.001671 | 1.71E-12 | 332021 | 0.56452 | height_10 |  |  | |
| rs1052169 | 17 | 43189049 | C | 0.014535 | 0.002151 | 1.42E-11 | 332021 | 0.183782 | height_10 |  |  | |
| rs55938136 | 17 | 43798360 | G | -0.01123 | 0.00198 | 1.40E-08 | 332021 | 0.226206 | height_10 |  |  | |
| rs10520770 | 18 | 46602964 | C | 0.011029 | 0.001668 | 3.80E-11 | 332021 | 0.448615 | height_10 |  |  | |
| rs2543005 | 18 | 43049872 | C | 0.010292 | 0.001857 | 2.98E-08 | 332021 | 0.274925 | height_10 |  |  | |
| rs8091465 | 18 | 22811960 | T | -0.00992 | 0.00173 | 9.86E-09 | 332021 | 0.356987 | height_10 |  |  | |
| rs9964411 | 18 | 29737002 | G | 0.012898 | 0.002278 | 1.49E-08 | 332021 | 0.16039 | height_10 |  |  | |
| rs4369779 | 18 | 20735408 | C | 0.040848 | 0.002022 | 1.12E-90 | 332021 | 0.787313 | height_10 |  |  | |
| rs17522826 | 18 | 53070914 | A | 0.015512 | 0.002141 | 4.28E-13 | 332021 | 0.188236 | height_10 |  |  | |
| rs571312 | 18 | 57839769 | A | 0.012986 | 0.001954 | 3.05E-11 | 332021 | 0.234442 | height_10 |  |  | |
| rs688559 | 19 | 39471120 | G | 0.009276 | 0.001698 | 4.65E-08 | 332021 | 0.549992 | height_10 |  |  | |
| rs10948 | 19 | 10754905 | T | -0.01231 | 0.001757 | 2.44E-12 | 332021 | 0.664051 | height_10 |  |  | |
| rs12462204 | 19 | 4772763 | C | -0.01383 | 0.002462 | 1.92E-08 | 332021 | 0.131142 | height_10 |  |  | |
| rs34840745 | 19 | 7183797 | T | 0.01779 | 0.001888 | 4.52E-21 | 332021 | 0.261832 | height_10 |  |  | |
| rs1043413 | 19 | 41939297 | G | 0.014834 | 0.001698 | 2.43E-18 | 332021 | 0.388111 | height_10 |  |  | |
| rs147110934 | 19 | 55993436 | T | -0.04588 | 0.0054 | 1.96E-17 | 332021 | 0.024103 | height_10 |  |  | |
| rs67820526 | 19 | 4083916 | A | 0.012778 | 0.002184 | 4.92E-09 | 332021 | 0.179797 | height_10 |  |  | |
| rs7251031 | 19 | 11266693 | G | -0.01347 | 0.001805 | 8.57E-14 | 332021 | 0.306544 | height_10 |  |  | |
| rs2854502 | 19 | 44053542 | A | 0.01194 | 0.002117 | 1.70E-08 | 332021 | 0.189115 | height_10 |  |  | |
| rs4802273 | 19 | 46244060 | G | 0.009907 | 0.001812 | 4.57E-08 | 332021 | 0.298442 | height_10 |  |  | |
| rs751858 | 19 | 19602821 | C | -0.01536 | 0.002151 | 9.24E-13 | 332021 | 0.181857 | height_10 |  |  | |
| rs2228612 | 19 | 10273372 | C | 0.021526 | 0.003386 | 2.06E-10 | 332021 | 0.0636924 | height_10 |  |  | |
| rs149202222 | 19 | 11153574 | T | -0.0204 | 0.003373 | 1.48E-09 | 332021 | 0.0661496 | height_10 |  |  | |
| rs11667331 | 19 | 31050571 | G | 0.026554 | 0.002231 | 1.19E-32 | 332021 | 0.165932 | height_10 |  |  | |
| rs7254564 | 19 | 12169180 | C | 0.019034 | 0.003127 | 1.15E-09 | 332021 | 0.0762295 | height_10 |  |  | |
| rs2271881 | 19 | 18305824 | T | 0.009727 | 0.001729 | 1.85E-08 | 332021 | 0.35872 | height_10 |  |  | |
| rs12980849 | 19 | 37651714 | T | -0.01266 | 0.001661 | 2.52E-14 | 332021 | 0.524235 | height_10 |  |  | |
| rs516316 | 19 | 49206145 | C | -0.01055 | 0.001663 | 2.29E-10 | 332021 | 0.508363 | height_10 |  |  | |
| rs12973528 | 19 | 2155497 | T | 0.019601 | 0.001703 | 1.18E-30 | 332021 | 0.396861 | height_10 |  |  | |
| rs12980469 | 19 | 3457058 | A | 0.013879 | 0.001973 | 2.00E-12 | 332021 | 0.229593 | height_10 |  |  | |
| rs62621197 | 19 | 8670147 | T | -0.08399 | 0.004591 | 9.73E-75 | 332021 | 0.035921 | height_10 |  |  | |
| rs2279008 | 19 | 17283303 | C | -0.0131 | 0.001909 | 6.91E-12 | 332021 | 0.251719 | height_10 |  |  | |
| rs12975297 | 19 | 56025654 | T | 0.011568 | 0.002042 | 1.47E-08 | 332021 | 0.210308 | height_10 |  |  | |
| rs2602713 | 19 | 4910021 | C | 0.012868 | 0.001686 | 2.28E-14 | 332021 | 0.436771 | height_10 |  |  | |
| rs10412446 | 19 | 50067508 | G | 0.012113 | 0.001985 | 1.05E-09 | 332021 | 0.227203 | height_10 |  |  | |
| rs2083919 | 20 | 5109402 | C | -0.01637 | 0.002649 | 6.35E-10 | 332021 | 0.110239 | height_10 |  |  | |
| rs3213150 | 20 | 32272201 | A | -0.01834 | 0.00183 | 1.23E-23 | 332021 | 0.294092 | height_10 |  |  | |
| rs45465702 | 20 | 33320571 | C | 0.023888 | 0.002139 | 5.81E-29 | 332021 | 0.184066 | height_10 |  |  | |
| rs2050091 | 20 | 35838591 | C | 0.042431 | 0.004345 | 1.59E-22 | 332021 | 0.958877 | height_10 |  |  | |
| rs6127699 | 20 | 54825592 | C | -0.03999 | 0.005535 | 5.02E-13 | 332021 | 0.0229212 | height_10 |  |  | |
| rs13038881 | 20 | 31468113 | C | 0.009812 | 0.001662 | 3.52E-09 | 332021 | 0.531954 | height_10 |  |  | |
| rs143384 | 20 | 34025756 | G | 0.044806 | 0.001687 | 2.77E-155 | 332021 | 0.402796 | height_10 |  |  | |
| rs151197637 | 20 | 34218087 | A | 0.041849 | 0.007144 | 4.69E-09 | 332021 | 0.0145063 | height_10 |  |  | |
| rs78319794 | 20 | 33906639 | C | -0.03131 | 0.005558 | 1.76E-08 | 332021 | 0.0226246 | height_10 |  |  | |
| rs34580798 | 20 | 47899957 | C | 0.023037 | 0.001995 | 7.76E-31 | 332021 | 0.221108 | height_10 |  |  | |
| rs76940308 | 20 | 54808024 | T | 0.016528 | 0.002744 | 1.70E-09 | 332021 | 0.102012 | height_10 |  |  | |
| rs2182356 | 20 | 54859034 | T | -0.01882 | 0.001872 | 8.96E-24 | 332021 | 0.733092 | height_10 |  |  | |
| rs6054392 | 20 | 6594443 | C | -0.02412 | 0.001716 | 7.49E-45 | 332021 | 0.629802 | height_10 |  |  | |
| rs6026579 | 20 | 57463993 | T | -0.01021 | 0.001758 | 6.39E-09 | 332021 | 0.659726 | height_10 |  |  | |
| rs1291132 | 20 | 35512452 | A | 0.010663 | 0.001685 | 2.50E-10 | 332021 | 0.41126 | height_10 |  |  | |
| rs61734651 | 20 | 61451332 | T | 0.0239 | 0.003348 | 9.39E-13 | 332021 | 0.0706128 | height_10 |  |  | |
| rs4810897 | 20 | 47535404 | C | -0.01436 | 0.002455 | 4.91E-09 | 332021 | 0.131682 | height_10 |  |  | |
| rs235763 | 20 | 6705246 | C | -0.01192 | 0.001698 | 2.21E-12 | 332021 | 0.401097 | height_10 |  |  | |
| rs1741344 | 20 | 4101800 | T | -0.0163 | 0.001719 | 2.49E-21 | 332021 | 0.63264 | height_10 |  |  | |
| rs1467847 | 21 | 35714544 | C | 0.011171 | 0.001676 | 2.65E-11 | 332021 | 0.567837 | height_10 |  |  | |
| rs1892684 | 21 | 39650903 | G | -0.01422 | 0.00167 | 1.68E-17 | 332021 | 0.558802 | height_10 |  |  | |
| rs713875 | 22 | 30592487 | G | -0.01104 | 0.001667 | 3.57E-11 | 332021 | 0.557539 | height_10 |  |  | |
| rs11913403 | 22 | 33106737 | G | -0.01539 | 0.002344 | 5.07E-11 | 332021 | 0.146937 | height_10 |  |  | |
| rs13058335 | 22 | 18910479 | T | 0.017898 | 0.003151 | 1.35E-08 | 332021 | 0.0765646 | height_10 |  |  | |
| rs4820324 | 22 | 38599857 | C | -0.01187 | 0.001682 | 1.72E-12 | 332021 | 0.581261 | height_10 |  |  | |
| rs2157314 | 22 | 46246890 | G | 0.010792 | 0.001688 | 1.62E-10 | 332021 | 0.457424 | height_10 |  |  | |
| rs5749937 | 22 | 22140136 | T | 0.009266 | 0.00166 | 2.37E-08 | 332021 | 0.50907 | height_10 |  |  | |
| rs9330813 | 22 | 46364161 | A | -0.01087 | 0.001783 | 1.08E-09 | 332021 | 0.316128 | height_10 |  |  | |
| rs5763244 | 22 | 20789190 | C | 0.010169 | 0.001672 | 1.19E-09 | 332021 | 0.428931 | height_10 | 0.061 | 33.14 | |
| rs12042908 | 1 | 74997762 | G | -0.0276 | 0.001677 | 7.83E-61 | 331693 | 0.562344 | body_size_10 |  |  | |
| rs11209943 | 1 | 72750500 | G | 0.018923 | 0.001694 | 5.85E-29 | 331693 | 0.599711 | body_size_10 |  |  | |
| rs2767486 | 1 | 65991203 | G | 0.015111 | 0.002079 | 3.66E-13 | 331693 | 0.20053 | body_size_10 |  |  | |
| rs9438393 | 1 | 205782718 | G | -0.01017 | 0.001689 | 1.71E-09 | 331693 | 0.415765 | body_size_10 |  |  | |
| rs543874 | 1 | 177889480 | G | 0.046892 | 0.002046 | 3.68E-116 | 331693 | 0.207808 | body_size_10 |  |  | |
| rs12140153 | 1 | 62579891 | T | -0.02232 | 0.002885 | 1.03E-14 | 331693 | 0.0967607 | body_size_10 |  |  | |
| rs72683129 | 1 | 66069781 | G | -0.01359 | 0.00236 | 8.60E-09 | 331693 | 0.149593 | body_size_10 |  |  | |
| rs7534091 | 1 | 118864616 | G | 0.012094 | 0.001895 | 1.73E-10 | 331693 | 0.260216 | body_size_10 |  |  | |
| rs2229330 | 1 | 6649228 | G | 0.021752 | 0.003214 | 1.31E-11 | 331693 | 0.0718419 | body_size_10 |  |  | |
| rs10399787 | 1 | 7032629 | A | -0.01027 | 0.00169 | 1.24E-09 | 331693 | 0.418842 | body_size_10 |  |  | |
| rs7550711 | 1 | 110082886 | T | 0.051713 | 0.005247 | 6.52E-23 | 331693 | 0.0259328 | body_size_10 |  |  | |
| rs587271 | 1 | 54743111 | T | 0.011241 | 0.001871 | 1.86E-09 | 331693 | 0.687423 | body_size_10 |  |  | |
| rs72678994 | 1 | 72350609 | C | 0.015076 | 0.002749 | 4.15E-08 | 331693 | 0.113566 | body_size_10 |  |  | |
| rs1384660 | 2 | 142299735 | A | -0.01716 | 0.002143 | 1.17E-15 | 331693 | 0.184775 | body_size_10 |  |  | |
| rs12713889 | 2 | 77225361 | C | -0.01312 | 0.001769 | 1.19E-13 | 331693 | 0.329676 | body_size_10 |  |  | |
| rs1706252 | 2 | 188156578 | C | 0.009827 | 0.00178 | 3.38E-08 | 331693 | 0.678101 | body_size_10 |  |  | |
| rs13401686 | 2 | 650519 | G | -0.04253 | 0.002199 | 2.73E-83 | 331693 | 0.171947 | body_size_10 |  |  | |
| rs2384054 | 2 | 25156773 | C | 0.035375 | 0.00166 | 1.14E-100 | 331693 | 0.489674 | body_size_10 |  |  | |
| rs115319174 | 2 | 207066474 | C | 0.043785 | 0.00361 | 7.50E-34 | 331693 | 0.0577374 | body_size_10 |  |  | |
| rs62104184 | 2 | 542879 | A | 0.02332 | 0.002982 | 5.26E-15 | 331693 | 0.0909077 | body_size_10 |  |  | |
| rs657667 | 2 | 67693591 | A | -0.01005 | 0.001701 | 3.56E-09 | 331693 | 0.581252 | body_size_10 |  |  | |
| rs62106258 | 2 | 417167 | C | -0.07916 | 0.003869 | 5.44E-93 | 331693 | 0.0484669 | body_size_10 |  |  | |
| rs72760962 | 2 | 710284 | C | 0.025503 | 0.004379 | 5.75E-09 | 331693 | 0.0372703 | body_size_10 |  |  | |
| rs2693887 | 2 | 6211461 | T | -0.01741 | 0.003052 | 1.18E-08 | 331693 | 0.0820895 | body_size_10 |  |  | |
| rs13025101 | 2 | 45130956 | A | 0.012157 | 0.002062 | 3.74E-09 | 331693 | 0.205367 | body_size_10 |  |  | |
| rs149838931 | 2 | 24694250 | A | 0.043939 | 0.006178 | 1.15E-12 | 331693 | 0.0200816 | body_size_10 |  |  | |
| rs13428812 | 2 | 25492467 | G | 0.015096 | 0.001797 | 4.51E-17 | 331693 | 0.309375 | body_size_10 |  |  | |
| rs58741371 | 2 | 119633581 | C | -0.01286 | 0.002114 | 1.19E-09 | 331693 | 0.198314 | body_size_10 |  |  | |
| rs10207816 | 2 | 207119641 | G | -0.01137 | 0.001923 | 3.36E-09 | 331693 | 0.249863 | body_size_10 |  |  | |
| rs1040319 | 3 | 88098062 | G | 0.01321 | 0.002237 | 3.50E-09 | 331693 | 0.834752 | body_size_10 |  |  | |
| rs60654199 | 3 | 141267295 | A | 0.022066 | 0.003328 | 3.36E-11 | 331693 | 0.0666476 | body_size_10 |  |  | |
| rs7355953 | 3 | 85792137 | C | 0.017565 | 0.002031 | 5.35E-18 | 331693 | 0.21342 | body_size_10 |  |  | |
| rs7625768 | 3 | 131774642 | A | 0.01091 | 0.00179 | 1.10E-09 | 331693 | 0.317224 | body_size_10 |  |  | |
| rs2034963 | 3 | 48170802 | C | -0.01088 | 0.001755 | 5.69E-10 | 331693 | 0.648697 | body_size_10 |  |  | |
| rs7432587 | 3 | 86199354 | G | -0.00939 | 0.001683 | 2.42E-08 | 331693 | 0.565108 | body_size_10 |  |  | |
| rs1603977 | 3 | 25108179 | A | 0.010484 | 0.001819 | 8.30E-09 | 331693 | 0.700745 | body_size_10 |  |  | |
| rs1308362 | 3 | 138103927 | T | -0.01318 | 0.002117 | 4.87E-10 | 331693 | 0.810225 | body_size_10 |  |  | |
| rs355748 | 3 | 153964496 | T | 0.00939 | 0.00172 | 4.82E-08 | 331693 | 0.376764 | body_size_10 |  |  | |
| rs347617 | 3 | 11278211 | T | -0.01368 | 0.001773 | 1.22E-14 | 331693 | 0.670829 | body_size_10 |  |  | |
| rs776106 | 3 | 77742551 | A | -0.0108 | 0.001899 | 1.31E-08 | 331693 | 0.742295 | body_size_10 |  |  | |
| rs11920002 | 3 | 131616345 | T | -0.02202 | 0.003266 | 1.57E-11 | 331693 | 0.0701915 | body_size_10 |  |  | |
| rs77771121 | 3 | 61221102 | T | -0.01826 | 0.002304 | 2.31E-15 | 331693 | 0.154137 | body_size_10 |  |  | |
| rs78332833 | 4 | 41770126 | G | -0.01852 | 0.003309 | 2.19E-08 | 331693 | 0.0716906 | body_size_10 |  |  | |
| rs35443290 | 4 | 44503166 | G | -0.01165 | 0.002052 | 1.37E-08 | 331693 | 0.217714 | body_size_10 |  |  | |
| rs7377083 | 4 | 102708997 | A | 0.014327 | 0.00169 | 2.28E-17 | 331693 | 0.431316 | body_size_10 |  |  | |
| rs13113232 | 4 | 137151705 | C | -0.00958 | 0.001722 | 2.64E-08 | 331693 | 0.370839 | body_size_10 |  |  | |
| rs34811474 | 4 | 25408838 | A | -0.01348 | 0.001967 | 7.19E-12 | 331693 | 0.231875 | body_size_10 |  |  | |
| rs788860 | 4 | 82137639 | A | -0.01255 | 0.00183 | 7.10E-12 | 331693 | 0.294396 | body_size_10 |  |  | |
| rs2955476 | 4 | 130741313 | T | -0.00987 | 0.001696 | 5.96E-09 | 331693 | 0.594714 | body_size_10 |  |  | |
| rs13141110 | 4 | 30837846 | A | 0.012475 | 0.001692 | 1.68E-13 | 331693 | 0.407747 | body_size_10 |  |  | |
| rs4449446 | 4 | 42155174 | A | 0.011288 | 0.001958 | 8.17E-09 | 331693 | 0.237685 | body_size_10 |  |  | |
| rs10938397 | 4 | 45182527 | G | 0.021962 | 0.001681 | 5.25E-39 | 331693 | 0.434075 | body_size_10 |  |  | |
| rs918472 | 5 | 170738836 | A | 0.011035 | 0.001855 | 2.69E-09 | 331693 | 0.717778 | body_size_10 |  |  | |
| rs1346482 | 5 | 153535857 | A | -0.01276 | 0.001663 | 1.67E-14 | 331693 | 0.53319 | body_size_10 |  |  | |
| rs154619 | 5 | 66200447 | C | 0.011169 | 0.001859 | 1.89E-09 | 331693 | 0.723189 | body_size_10 |  |  | |
| rs9291816 | 5 | 63932508 | T | -0.01339 | 0.001775 | 4.72E-14 | 331693 | 0.324522 | body_size_10 |  |  | |
| rs7727824 | 5 | 60706426 | C | -0.01041 | 0.001731 | 1.83E-09 | 331693 | 0.359952 | body_size_10 |  |  | |
| rs2115885 | 5 | 87598818 | A | -0.01415 | 0.002075 | 9.33E-12 | 331693 | 0.204422 | body_size_10 |  |  | |
| rs13175179 | 5 | 156781955 | A | 0.012149 | 0.002044 | 2.78E-09 | 331693 | 0.210278 | body_size_10 |  |  | |
| rs1422067 | 5 | 77424836 | T | -0.01164 | 0.001949 | 2.33E-09 | 331693 | 0.238272 | body_size_10 |  |  | |
| rs254672 | 5 | 157246215 | G | -0.00985 | 0.001773 | 2.73E-08 | 331693 | 0.328302 | body_size_10 |  |  | |
| rs2307111 | 5 | 75003678 | C | -0.00976 | 0.001704 | 1.00E-08 | 331693 | 0.393183 | body_size_10 |  |  | |
| rs6875288 | 5 | 144506078 | G | 0.009134 | 0.001664 | 4.07E-08 | 331693 | 0.512844 | body_size_10 |  |  | |
| rs77960 | 5 | 103964585 | A | -0.01008 | 0.001771 | 1.28E-08 | 331693 | 0.32864 | body_size_10 |  |  | |
| rs6457808 | 6 | 35143465 | T | 0.034253 | 0.006224 | 3.73E-08 | 331693 | 0.0181139 | body_size_10 |  |  | |
| rs3131934 | 6 | 30931844 | C | 0.019686 | 0.002205 | 4.37E-19 | 331693 | 0.172372 | body_size_10 |  |  | |
| rs987237 | 6 | 50803050 | G | 0.026392 | 0.002173 | 6.35E-34 | 331693 | 0.178318 | body_size_10 |  |  | |
| rs4487589 | 6 | 100178143 | T | 0.01171 | 0.002081 | 1.83E-08 | 331693 | 0.19847 | body_size_10 |  |  | |
| rs201237 | 6 | 10020243 | G | -0.01238 | 0.001747 | 1.38E-12 | 331693 | 0.345609 | body_size_10 |  |  | |
| rs3884025 | 6 | 25930088 | A | 0.017816 | 0.002774 | 1.33E-10 | 331693 | 0.100073 | body_size_10 |  |  | |
| rs9272550 | 6 | 32606970 | A | 0.013127 | 0.002263 | 6.57E-09 | 331693 | 0.212522 | body_size_10 |  |  | |
| rs9391254 | 6 | 105377347 | T | -0.0102 | 0.001778 | 9.62E-09 | 331693 | 0.321611 | body_size_10 |  |  | |
| rs1452991 | 6 | 141473363 | A | 0.010922 | 0.001731 | 2.82E-10 | 331693 | 0.371715 | body_size_10 |  |  | |
| rs6931604 | 6 | 98578215 | T | 0.010235 | 0.001703 | 1.85E-09 | 331693 | 0.602614 | body_size_10 |  |  | |
| rs34260097 | 6 | 100727703 | G | 0.016879 | 0.001988 | 2.04E-17 | 331693 | 0.225481 | body_size_10 |  |  | |
| rs6921067 | 6 | 50352150 | A | 0.010577 | 0.001796 | 3.90E-09 | 331693 | 0.688318 | body_size_10 |  |  | |
| rs62425398 | 6 | 166416028 | A | 0.01612 | 0.002699 | 2.35E-09 | 331693 | 0.107214 | body_size_10 |  |  | |
| rs13211684 | 6 | 51175486 | C | 0.014173 | 0.001718 | 1.56E-16 | 331693 | 0.380507 | body_size_10 |  |  | |
| rs1294093 | 6 | 154332947 | G | -0.01365 | 0.001813 | 5.23E-14 | 331693 | 0.302956 | body_size_10 |  |  | |
| rs12110721 | 6 | 55190480 | A | 0.018014 | 0.002236 | 7.76E-16 | 331693 | 0.1761 | body_size_10 |  |  | |
| rs9382623 | 6 | 56218157 | A | 0.010614 | 0.001926 | 3.57E-08 | 331693 | 0.248936 | body_size_10 |  |  | |
| rs507809 | 6 | 54293766 | T | -0.01968 | 0.003326 | 3.29E-09 | 331693 | 0.0692544 | body_size_10 |  |  | |
| rs73422097 | 6 | 41727740 | G | 0.012336 | 0.001813 | 1.03E-11 | 331693 | 0.299415 | body_size_10 |  |  | |
| rs9474773 | 6 | 54114363 | T | 0.025234 | 0.003699 | 8.99E-12 | 331693 | 0.0534996 | body_size_10 |  |  | |
| rs4727295 | 7 | 76636756 | A | -0.01262 | 0.002214 | 1.19E-08 | 331693 | 0.828097 | body_size_10 |  |  | |
| rs1852006 | 7 | 77829768 | A | -0.01054 | 0.001735 | 1.24E-09 | 331693 | 0.356006 | body_size_10 |  |  | |
| rs4727289 | 7 | 93026328 | T | -0.0112 | 0.001958 | 1.06E-08 | 331693 | 0.23954 | body_size_10 |  |  | |
| rs6944123 | 7 | 103128824 | T | 0.0106 | 0.001789 | 3.16E-09 | 331693 | 0.314451 | body_size_10 |  |  | |
| rs6972277 | 7 | 24306230 | A | -0.01194 | 0.00173 | 5.09E-12 | 331693 | 0.635807 | body_size_10 |  |  | |
| rs11525873 | 7 | 138817193 | C | -0.01608 | 0.002792 | 8.40E-09 | 331693 | 0.0983855 | body_size_10 |  |  | |
| rs982692 | 7 | 93206613 | C | 0.014347 | 0.001729 | 1.08E-16 | 331693 | 0.361064 | body_size_10 |  |  | |
| rs4731419 | 7 | 127850795 | T | 0.011405 | 0.001707 | 2.38E-11 | 331693 | 0.581465 | body_size_10 |  |  | |
| rs13256357 | 8 | 9979535 | T | 0.015386 | 0.002073 | 1.15E-13 | 331693 | 0.202489 | body_size_10 |  |  | |
| rs2656288 | 8 | 4138427 | A | -0.01171 | 0.001838 | 1.85E-10 | 331693 | 0.708856 | body_size_10 |  |  | |
| rs13254935 | 8 | 41201081 | C | -0.01431 | 0.002577 | 2.80E-08 | 331693 | 0.117683 | body_size_10 |  |  | |
| rs2126474 | 8 | 76878957 | T | -0.01743 | 0.001689 | 5.78E-25 | 331693 | 0.413168 | body_size_10 |  |  | |
| rs13254613 | 8 | 64804804 | C | 0.013209 | 0.001751 | 4.59E-14 | 331693 | 0.344832 | body_size_10 |  |  | |
| rs881299 | 8 | 38332249 | C | 0.010032 | 0.001685 | 2.63E-09 | 331693 | 0.412418 | body_size_10 |  |  | |
| rs12001083 | 9 | 120404878 | T | -0.01148 | 0.001771 | 9.08E-11 | 331693 | 0.329948 | body_size_10 |  |  | |
| rs57263785 | 9 | 2197987 | G | -0.01172 | 0.001931 | 1.29E-09 | 331693 | 0.246003 | body_size_10 |  |  | |
| rs1935354 | 9 | 27822531 | C | 0.011238 | 0.001667 | 1.58E-11 | 331693 | 0.484337 | body_size_10 |  |  | |
| rs112216627 | 9 | 96181202 | A | -0.01954 | 0.003409 | 1.00E-08 | 331693 | 0.0636909 | body_size_10 |  |  | |
| rs818902 | 9 | 6968603 | A | -0.01101 | 0.001794 | 8.46E-10 | 331693 | 0.683414 | body_size_10 |  |  | |
| rs3118249 | 9 | 25103035 | T | 0.009349 | 0.001684 | 2.82E-08 | 331693 | 0.570884 | body_size_10 |  |  | |
| rs7856471 | 9 | 32328019 | C | 0.01109 | 0.001894 | 4.75E-09 | 331693 | 0.264331 | body_size_10 |  |  | |
| rs2149564 | 9 | 98607989 | T | -0.0115 | 0.001665 | 5.03E-12 | 331693 | 0.481695 | body_size_10 |  |  | |
| rs2275241 | 9 | 129370576 | A | 0.010371 | 0.00172 | 1.64E-09 | 331693 | 0.371494 | body_size_10 |  |  | |
| rs10821163 | 9 | 96343060 | C | 0.015166 | 0.001762 | 7.51E-18 | 331693 | 0.343053 | body_size_10 |  |  | |
| rs12343333 | 9 | 109620811 | C | -0.01003 | 0.001815 | 3.23E-08 | 331693 | 0.300323 | body_size_10 |  |  | |
| rs3814119 | 9 | 129462901 | C | 0.010749 | 0.001682 | 1.68E-10 | 331693 | 0.441942 | body_size_10 |  |  | |
| rs117911387 | 9 | 130446836 | A | 0.026129 | 0.00394 | 3.32E-11 | 331693 | 0.0466698 | body_size_10 |  |  | |
| rs7084503 | 10 | 2666859 | C | -0.01194 | 0.001671 | 8.92E-13 | 331693 | 0.506738 | body_size_10 |  |  | |
| rs4572029 | 10 | 70889053 | G | -0.01166 | 0.002086 | 2.25E-08 | 331693 | 0.203687 | body_size_10 |  |  | |
| rs11256627 | 10 | 10535954 | A | 0.010784 | 0.001834 | 4.13E-09 | 331693 | 0.705761 | body_size_10 |  |  | |
| rs41310284 | 10 | 102447647 | A | -0.01929 | 0.002768 | 3.21E-12 | 331693 | 0.101545 | body_size_10 |  |  | |
| rs4075752 | 10 | 2085106 | C | -0.0104 | 0.001666 | 4.34E-10 | 331693 | 0.498353 | body_size_10 |  |  | |
| rs17399739 | 10 | 87490850 | G | 0.019211 | 0.003268 | 4.12E-09 | 331693 | 0.0695257 | body_size_10 |  |  | |
| rs6484440 | 11 | 29310910 | T | 0.010508 | 0.001667 | 2.94E-10 | 331693 | 0.486257 | body_size_10 |  |  | |
| rs10796828 | 11 | 69490346 | G | 0.011259 | 0.00173 | 7.56E-11 | 331693 | 0.635426 | body_size_10 |  |  | |
| rs10790809 | 11 | 126372550 | G | 0.009424 | 0.001673 | 1.79E-08 | 331693 | 0.559426 | body_size_10 |  |  | |
| rs11030119 | 11 | 27728102 | A | 0.015226 | 0.0018 | 2.74E-17 | 331693 | 0.308813 | body_size_10 |  |  | |
| rs2958542 | 11 | 62181882 | T | -0.01045 | 0.001737 | 1.81E-09 | 331693 | 0.366328 | body_size_10 |  |  | |
| rs10835356 | 11 | 28565801 | A | 0.012083 | 0.001667 | 4.21E-13 | 331693 | 0.502384 | body_size_10 |  |  | |
| rs11215403 | 11 | 115058585 | A | -0.011 | 0.001935 | 1.33E-08 | 331693 | 0.244354 | body_size_10 |  |  | |
| rs2303385 | 11 | 65640562 | A | -0.01133 | 0.001727 | 5.37E-11 | 331693 | 0.639677 | body_size_10 |  |  | |
| rs10896348 | 11 | 68357368 | C | -0.01341 | 0.001852 | 4.47E-13 | 331693 | 0.277766 | body_size_10 |  |  | |
| rs11039307 | 11 | 47611152 | T | 0.014792 | 0.001689 | 1.96E-18 | 331693 | 0.408314 | body_size_10 |  |  | |
| rs1402954 | 11 | 33777334 | T | 0.016046 | 0.002901 | 3.20E-08 | 331693 | 0.090328 | body_size_10 |  |  | |
| rs11170468 | 12 | 39430048 | C | -0.01107 | 0.001963 | 1.68E-08 | 331693 | 0.234547 | body_size_10 |  |  | |
| rs9669278 | 12 | 66374587 | C | -0.0097 | 0.001673 | 6.84E-09 | 331693 | 0.516489 | body_size_10 |  |  | |
| rs7132908 | 12 | 50263148 | A | 0.032826 | 0.00171 | 4.09E-82 | 331693 | 0.38392 | body_size_10 |  |  | |
| rs10842356 | 12 | 24621348 | T | -0.00945 | 0.001661 | 1.26E-08 | 331693 | 0.513956 | body_size_10 |  |  | |
| rs7299842 | 12 | 122492313 | A | 0.009242 | 0.001687 | 4.31E-08 | 331693 | 0.438843 | body_size_10 |  |  | |
| rs12817542 | 12 | 103736499 | T | 0.021236 | 0.003476 | 1.00E-09 | 331693 | 0.0607875 | body_size_10 |  |  | |
| rs7978659 | 12 | 49507127 | T | 0.014416 | 0.001753 | 1.99E-16 | 331693 | 0.342761 | body_size_10 |  |  | |
| rs860698 | 12 | 50486322 | C | -0.01003 | 0.001688 | 2.87E-09 | 331693 | 0.4124 | body_size_10 |  |  | |
| rs2187642 | 12 | 11855624 | C | 0.010531 | 0.001715 | 8.23E-10 | 331693 | 0.623274 | body_size_10 |  |  | |
| rs78607331 | 12 | 57648644 | T | 0.026535 | 0.003987 | 2.84E-11 | 331693 | 0.0454183 | body_size_10 |  |  | |
| rs55726687 | 12 | 991306 | A | 0.015972 | 0.002036 | 4.39E-15 | 331693 | 0.211138 | body_size_10 |  |  | |
| rs11612454 | 12 | 99658692 | T | 0.010735 | 0.001911 | 1.93E-08 | 331693 | 0.254249 | body_size_10 |  |  | |
| rs7305424 | 12 | 118399491 | T | 0.010731 | 0.00176 | 1.08E-09 | 331693 | 0.340501 | body_size_10 |  |  | |
| rs12230843 | 12 | 20105947 | G | 0.009549 | 0.001675 | 1.18E-08 | 331693 | 0.529484 | body_size_10 |  |  | |
| rs9538141 | 13 | 59178258 | A | 0.011955 | 0.001674 | 9.12E-13 | 331693 | 0.512986 | body_size_10 |  |  | |
| rs1441264 | 13 | 79580919 | A | 0.012722 | 0.001727 | 1.78E-13 | 331693 | 0.591866 | body_size_10 |  |  | |
| rs9568868 | 13 | 54107583 | T | 0.023041 | 0.002497 | 2.78E-20 | 331693 | 0.129002 | body_size_10 |  |  | |
| rs7997379 | 13 | 27936117 | G | -0.00926 | 0.001678 | 3.40E-08 | 331693 | 0.428963 | body_size_10 |  |  | |
| rs2504235 | 13 | 28612886 | G | -0.01466 | 0.00173 | 2.41E-17 | 331693 | 0.638613 | body_size_10 |  |  | |
| rs1336486 | 13 | 40784814 | G | 0.013122 | 0.001769 | 1.21E-13 | 331693 | 0.3288 | body_size_10 |  |  | |
| rs9507895 | 13 | 28018237 | C | -0.01244 | 0.00187 | 2.84E-11 | 331693 | 0.27626 | body_size_10 |  |  | |
| rs1333010 | 13 | 66205228 | A | -0.0123 | 0.001704 | 5.26E-13 | 331693 | 0.608556 | body_size_10 |  |  | |
| rs12883788 | 14 | 33303540 | T | 0.010348 | 0.001674 | 6.31E-10 | 331693 | 0.459307 | body_size_10 |  |  | |
| rs61980365 | 14 | 30500472 | A | 0.030692 | 0.004333 | 1.41E-12 | 331693 | 0.0385336 | body_size_10 |  |  | |
| rs76565625 | 14 | 80318018 | T | 0.022448 | 0.004035 | 2.65E-08 | 331693 | 0.0450817 | body_size_10 |  |  | |
| rs4899746 | 14 | 79943927 | G | 0.011743 | 0.001727 | 1.04E-11 | 331693 | 0.630298 | body_size_10 |  |  | |
| rs11073864 | 15 | 89989514 | T | 0.011858 | 0.002075 | 1.10E-08 | 331693 | 0.201906 | body_size_10 |  |  | |
| rs824207 | 15 | 24007729 | G | 0.00964 | 0.001665 | 7.08E-09 | 331693 | 0.535225 | body_size_10 |  |  | |
| rs28626095 | 15 | 68066729 | C | -0.02233 | 0.001995 | 4.57E-29 | 331693 | 0.222587 | body_size_10 |  |  | |
| rs3817428 | 15 | 89415247 | G | 0.01103 | 0.001877 | 4.18E-09 | 331693 | 0.267231 | body_size_10 |  |  | |
| rs72755233 | 15 | 100692953 | A | 0.017825 | 0.002624 | 1.10E-11 | 331693 | 0.112887 | body_size_10 |  |  | |
| rs16954988 | 16 | 55435388 | A | -0.01151 | 0.00206 | 2.29E-08 | 331693 | 0.205698 | body_size_10 |  |  | |
| rs55880046 | 16 | 19941557 | G | -0.0305 | 0.002381 | 1.47E-37 | 331693 | 0.14233 | body_size_10 |  |  | |
| rs34033929 | 16 | 4024128 | T | 0.018088 | 0.001718 | 6.41E-26 | 331693 | 0.596687 | body_size_10 |  |  | |
| rs4889631 | 16 | 30877636 | A | -0.01436 | 0.002076 | 4.55E-12 | 331693 | 0.798214 | body_size_10 |  |  | |
| rs62033415 | 16 | 53837369 | T | -0.02028 | 0.003136 | 9.99E-11 | 331693 | 0.077432 | body_size_10 |  |  | |
| rs9922288 | 16 | 24550930 | G | -0.01159 | 0.001979 | 4.69E-09 | 331693 | 0.762214 | body_size_10 |  |  | |
| rs78138943 | 16 | 67448533 | G | 0.033615 | 0.004663 | 5.65E-13 | 331693 | 0.0328471 | body_size_10 |  |  | |
| rs4238585 | 16 | 20255097 | T | 0.017077 | 0.002518 | 1.18E-11 | 331693 | 0.870582 | body_size_10 |  |  | |
| rs8047251 | 16 | 3708567 | A | 0.010173 | 0.001795 | 1.46E-08 | 331693 | 0.313189 | body_size_10 |  |  | |
| rs11641362 | 16 | 85335951 | T | -0.01128 | 0.001777 | 2.20E-10 | 331693 | 0.326903 | body_size_10 |  |  | |
| rs9929715 | 16 | 4000765 | C | -0.01098 | 0.001753 | 3.73E-10 | 331693 | 0.397891 | body_size_10 |  |  | |
| rs11642090 | 16 | 81730582 | C | 0.012433 | 0.00173 | 6.58E-13 | 331693 | 0.374986 | body_size_10 |  |  | |
| rs1421085 | 16 | 53800954 | C | 0.04885 | 0.001693 | 8.51E-183 | 331693 | 0.40212 | body_size_10 |  |  | |
| rs62037364 | 16 | 28868695 | A | 0.014249 | 0.001696 | 4.40E-17 | 331693 | 0.406868 | body_size_10 |  |  | |
| rs73614566 | 16 | 53563327 | C | 0.01415 | 0.002438 | 6.50E-09 | 331693 | 0.135079 | body_size_10 |  |  | |
| rs12952581 | 17 | 47448346 | A | 0.011908 | 0.001732 | 6.20E-12 | 331693 | 0.363275 | body_size_10 |  |  | |
| rs2246621 | 17 | 74084441 | T | -0.01045 | 0.001672 | 4.16E-10 | 331693 | 0.446105 | body_size_10 |  |  | |
| rs12601380 | 17 | 34904985 | C | -0.01071 | 0.001683 | 1.93E-10 | 331693 | 0.412618 | body_size_10 |  |  | |
| rs1555569 | 17 | 2149425 | C | -0.01291 | 0.001747 | 1.49E-13 | 331693 | 0.344841 | body_size_10 |  |  | |
| rs11150745 | 17 | 78757626 | G | -0.01251 | 0.001785 | 2.38E-12 | 331693 | 0.319489 | body_size_10 |  |  | |
| rs3874943 | 17 | 44572068 | G | -0.01089 | 0.001722 | 2.59E-10 | 331693 | 0.512441 | body_size_10 |  |  | |
| rs67603370 | 17 | 7524504 | A | 0.017759 | 0.003178 | 2.30E-08 | 331693 | 0.0768997 | body_size_10 |  |  | |
| rs2953016 | 17 | 29494693 | G | -0.01227 | 0.00204 | 1.81E-09 | 331693 | 0.78784 | body_size_10 |  |  | |
| rs657693 | 18 | 77162462 | G | 0.00955 | 0.001728 | 3.29E-08 | 331693 | 0.362511 | body_size_10 |  |  | |
| rs11662628 | 18 | 946727 | C | 0.010333 | 0.001691 | 9.93E-10 | 331693 | 0.584119 | body_size_10 |  |  | |
| rs3764516 | 18 | 52494374 | C | -0.01278 | 0.001963 | 7.59E-11 | 331693 | 0.764769 | body_size_10 |  |  | |
| rs663129 | 18 | 57838401 | A | 0.034566 | 0.001959 | 1.25E-69 | 331693 | 0.234384 | body_size_10 |  |  | |
| rs7239114 | 18 | 45921214 | A | 0.013308 | 0.00168 | 2.37E-15 | 331693 | 0.542958 | body_size_10 |  |  | |
| rs11873305 | 18 | 58049192 | C | -0.04355 | 0.004299 | 4.12E-24 | 331693 | 0.0391431 | body_size_10 |  |  | |
| rs8088123 | 18 | 58100400 | A | -0.01886 | 0.003233 | 5.44E-09 | 331693 | 0.0719812 | body_size_10 |  |  | |
| rs62621197 | 19 | 8670147 | T | 0.026698 | 0.004605 | 6.76E-09 | 331693 | 0.035921 | body_size_10 |  |  | |
| rs3810291 | 19 | 47569003 | A | 0.014906 | 0.001777 | 4.99E-17 | 331693 | 0.677478 | body_size_10 |  |  | |
| rs2315025 | 19 | 19426609 | T | -0.0101 | 0.001761 | 9.79E-09 | 331693 | 0.336361 | body_size_10 |  |  | |
| rs16982345 | 19 | 18500722 | A | 0.011806 | 0.001919 | 7.67E-10 | 331693 | 0.250741 | body_size_10 |  |  | |
| rs140036621 | 19 | 47709169 | T | 0.040619 | 0.006384 | 1.99E-10 | 331693 | 0.0185751 | body_size_10 |  |  | |
| rs10423928 | 19 | 46182304 | A | -0.0122 | 0.002099 | 6.19E-09 | 331693 | 0.19349 | body_size_10 |  |  | |
| rs6014536 | 20 | 54420996 | T | -0.01327 | 0.001929 | 6.01E-12 | 331693 | 0.247281 | body_size_10 |  |  | |
| rs73085586 | 20 | 22430241 | A | 0.012307 | 0.002078 | 3.15E-09 | 331693 | 0.201135 | body_size_10 |  |  | |
| rs16996644 | 20 | 15813475 | G | 0.018837 | 0.002496 | 4.51E-14 | 331693 | 0.127612 | body_size_10 |  |  | |
| rs947088 | 20 | 17171373 | T | 0.010826 | 0.001853 | 5.19E-09 | 331693 | 0.718359 | body_size_10 |  |  | |
| rs2024962 | 20 | 6613051 | A | 0.011183 | 0.001683 | 3.03E-11 | 331693 | 0.438894 | body_size_10 |  |  | |
| rs2281148 | 20 | 36433288 | C | 0.010778 | 0.001921 | 2.03E-08 | 331693 | 0.250994 | body_size_10 |  |  | |
| rs13047416 | 21 | 40309436 | G | -0.01126 | 0.001722 | 6.20E-11 | 331693 | 0.375427 | body_size_10 |  |  | |
| rs2876980 | 22 | 22106535 | C | 0.013998 | 0.002354 | 2.74E-09 | 331693 | 0.146715 | body_size_10 |  |  | |
| rs2072858 | 22 | 40708679 | C | -0.01263 | 0.001743 | 4.35E-13 | 331693 | 0.351908 | body_size_10 | 0.018 | 29.53 | |
| rs72853300 | 2 | 145638766 | T | 0.01896 | 0.00336 | 1.75E-08 | 341427 | 0.153 | smoking_age |  |  | |
| rs12611472 | 2 | 225353649 | C | 0.01842 | 0.00265 | 3.48E-12 | 341427 | 0.297 | smoking_age |  |  | |
| rs7559982 | 2 | 63622309 | A | -0.0172 | 0.00244 | 1.67E-12 | 341427 | 0.564 | smoking_age |  |  | |
| rs11915747 | 3 | 85699040 | G | 0.02022 | 0.00254 | 1.57E-15 | 339643 | 0.354 | smoking_age |  |  | |
| rs13136239 | 4 | 140908755 | A | 0.01481 | 0.00255 | 6.29E-09 | 341427 | 0.342 | smoking_age |  |  | |
| rs2471711 | 4 | 28589079 | T | -0.0192 | 0.00337 | 1.19E-08 | 341427 | 0.152 | smoking_age |  |  | |
| rs624833 | 4 | 2881256 | G | 0.01575 | 0.00264 | 2.36E-09 | 341427 | 0.302 | smoking_age |  |  | |
| rs7682598 | 4 | 68000888 | G | 0.01726 | 0.00288 | 2.09E-09 | 341427 | 0.771 | smoking_age |  |  | |
| rs1403174 | 7 | 2032865 | T | 0.0155 | 0.00245 | 2.5E-10 | 341427 | 0.579 | smoking_age |  |  | |
| rs11780471 | 8 | 27344719 | A | 0.03296 | 0.00509 | 9.44E-11 | 341427 | 0.0601 | smoking_age | 0.001 | 41.40 | |

Notes: Birth weight was measured in kg and age at smoking initiation was measured in years. Comparative body and height size at age 10 were collected through the question “When you were 10 years old, compared to average, would you describe yourself as…”. Participants’ body size was categorized as thinner, larger, or about average, while their height size was categorized as shorter, taller, or about average.

**Supplementary Table 6.** The mediation analysis of the adulthood factors in relation between early life exposures and hematological malignancy

|  | Outcomes | | | | | |  |
| --- | --- | --- | --- | --- | --- | --- | --- |
|  | Hematological Malignancy | *P* value | Leukemia | *P* value | Non-Hodgkin's lymphoma | *P* value |  |
| Cox regression model (HR) |  |  |  |  |  |  |  |
| Height at 10 | 0.908 (0.427, 1.932) | 0.802 | 0.270 (0.066, 1.100) | 0.068 | 1.728 (0.575, 5.190) | 0.330 |  |
| Mediator: Adulthood height | **1.015 (1.003, 1.026)** | **0.010** | 0.997 (0.976, 1.018) | 0.776 | **1.025 (1.008, 1.041)** | **0.003** |  |
| Interaction term | 1.000 (0.996, 1.005) | 0.766 | 1.008 (1.000, 1.017) | 0.053 | 0.997 (0.990, 1.003) | 0.348 |  |
| Linear/ model for the mediators | **5.518 (5.496, 5.540)** | **<0.001** | **5.518 (5.496, 5.540)** | **<0.001** | **5.518 (5.496, 5.540)** | **<0.001** |  |
| Overall proportion of mediation (%) | 88.08% | **<0.001** | 87.44% | **0.013** | 69.67% | **0.012** |  |
| Cox regression model (HR) |  |  |  |  |  |  |  |
| Body size at 10 | 0.871 (0.682, 1.112) | 0.268 | 0.716 (0.453, 1.132) | 0.153 | 0.881 (0.618, 1.256) | 0.484 |  |
| Mediator: BMI | 0.996 (0.978, 1.014) | 0.664 | 0.987 (0.955, 1.021) | 0.462 | 0.990 (0.965, 1.017) | 0.466 |  |
| Interaction term | 1.001 (0.999, 1.015) | 0.106 | 1.014 (0.998, 1.030) | 0.086 | 1.007 (0.995, 1.019) | 0.276 |  |
| Linear/ model for the mediators | **1.438 (1.418, 1.457)** | **<0.001** | **1.438 (1.418, 1.457)** | **<0.001** | **1.438 (1.418, 1.457)** | **<0.001** |  |
| Overall proportion of mediation (%) | 35.90% | 0.023 | 56.98% | 0.115 | 23.55% | 0.242 |  |
| Cox regression model for outcome(HR) |  |  |  |  |  |  |  |
| Young age at smoking initiation | 1.017 (0.982, 1.052) | 0.345 | **1.102 (1.035, 1.173)** | **0.002** | 0.993 (0.945, 1.044) | 0.777 |  |
| Mediator: current smoking | 1.011 (0.752, 1.360) | 0.942 | 1.232 (0.713, 2.129) | 0.454 | 0.805 (0.524, 1.236) | 0.321 |  |
| Interaction term | 1.055 (0.910, 1.224) | 0.475 | 0.927 (0.704, 1.220) | 0.588 | 1.222 (0.991, 1.506) | 0.061 |  |
| Linear/ model for the mediators | **1.248 (1.237, 1.259)** | **<0.001** | **1.248 (1.237, 1.259)** | **<0.001** | **1.248 (1.237, 1.259)** | **<0.001** |  |
| Overall proportion of mediation (%) | 63.38% | **0.033** | -0.04% | 0.923 | 103% | **0.003** |  |

**Supplementary Table 7.** Hazard ratio of leukemia subtypes (chronic lymphocytic leukemia and acute myelocytic leukemia) in the UK biobank cohort.

| **Cause of mortality** | **Leukemia** | | | | | | | |
| --- | --- | --- | --- | --- | --- | --- | --- | --- |
|  | **chronic lymphocytic leukemia** | | | | **acute myelocytic leukemia** | | | |
|  | **Total number** | **NO of events** | **Hazard ratio (95%CI)** | | **Total number** | **NO of events** | **Hazard ratio (95%CI)** | |
|  |  |  | **Model 1** | **Model 2** |  |  | **Model 1** | **Model 2** |
| **Maternal smoking around birth** | | |  |  |  |  |  |  |
| No | 305421 | 403 | 1.00 (REF) | 1.00 (REF) | 305616 | 208 | 1.00 (REF) | 1.00 (REF) |
| Yes | 126287 | 176 | 1.07 (0.90;1.28) | 1.09 (0.91;1.30) | 126380 | 83 | 1.00 (0.77;1.29) | 0.98 (0.75;1.26) |
| **Part of a multiple birth** | |  |  |  |  |  |  |  |
| No | 480793 | 659 | 1.00 (REF) | 1.00 (REF) | 481119 | 333 | 1.00 (REF) | 1.00 (REF) |
| Yes | 11205 | 12 | 0.80 (0.45;1.41) | 0.87 (0.49;1.55) | 11210 | 7 | 0.92 (0.43;1.94) | 0.88 (0.41;1.90) |
| **Birth weight** |  |  |  |  |  |  |  |  |
| <2.5kg | 28175 | 25 | 1.00 (REF) | 1.00 (REF) | 28180 | 20 | 1.00 (REF) | 1.00 (REF) |
| 2.5-4kg | 209990 | 244 | 1.35 (0.89;2.03) | 1.26 (0.83;1.92) | 210105 | 129 | 0.91 (0.57;1.46) | 0.86 (0.53;1.39) |
| >4kg | 38151 | 47 | 1.20 (0.74;1.95) | 1.07 (0.65;1.76) | 38168 | 30 | 1.02 (0.58;1.80) | 0.92 (0.51;1.64) |
| **Breastfed as a baby** | |  |  |  |  |  |  |  |
| No | 105875 | 99 | 1.00 (REF) | 1.00 (REF) | 105904 | 70 | 1.00 (REF) | 1.00 (REF) |
| Yes | 276833 | 391 | 1.14 (0.91;1.42) | 1.12 (0.89;1.40) | 277029 | 195 | 0.84 (0.64;1.11) | 0.85 (0.64;1.12) |
| **Comparative body size at age 10** | | |  |  |  |  |  |  |
| Thinner | 163227 | 213 | 1.00 (REF) | 1.00 (REF) | 163338 | 102 | 1.00 (REF) | 1.00 (REF) |
| About average | 248932 | 333 | 1.02 (0.86;1.21) | 1.02 (0.85;1.21) | 249089 | 176 | 1.12 (0.88;1.43) | 1.12 (0.87;1.43) |
| Larger | 77954 | 114 | 1.26 (1.00;1.58) | 1.24 (0.99;1.56) | 78007 | 61 | 1.37 (1.00;1.89) | 1.32 (0.96;1.82) |
| **Comparative height size at age 10** | | |  |  |  |  |  |  |
| Shorter | 100293 | 115 | 1.00 (REF) | 1.00 (REF) | 100350 | 58 | 1.00 (REF) | 1.00 (REF) |
| About average | 266483 | 358 | 1.13 (0.92;1.39) | 1.12 (0.91;1.39) | 266653 | 188 | 1.19 (0.89;1.60) | 1.18 (0.88;1.59) |
| Taller | 123975 | 190 | **1.33 (1.05;1.67)** | **1.31 (1.04;1.65)** | 124068 | 97 | 1.35 (0.98;1.87) | 1.32 (0.95;1.84) |
| **Smoking age** |  |  |  |  |  |  |  |  |
| No smoking | 272848 | 318 | 1.00 (REF) | 1.00 (REF) | 273019 | 147 | 1.00 (REF) | 1.00 (REF) |
| 5-14 | 25505 | 43 | 1.23 (0.89;1.70) | 1.22 (0.88;1.68) | 25524 | 24 | **1.66 (1.07;2.56)** | **1.65 (1.07;2.56)** |
| 15-18 | 93133 | 138 | 1.00 (0.81;1.22) | 0.99 (0.81;1.21) | 93178 | 93 | **1.56 (1.20;2.03)** | **1.55 (1.19;2.02)** |
| >18 | 39972 | 61 | 1.10 (0.84;1.45) | 1.09 (0.83;1.43) | 40006 | 27 | 1.08 (0.71;1.63) | 1.06 (0.70;1.61) |

^*^ Model 1 adjusted for age, sex, region of residence and ethnicity. Model 2 further adjusted for maternal smoking, multiple births, birth weight, being breastfed, body size at age 10, height size at age 10, and age at smoking initiation simultaneously. Statistically significant results are bolded. CI confidence interval, HR hazard ratio.

**Supplementary Table 8.** The associations between early-life exposures and risk of leukemia in the UK biobank cohort stratified by sex and age

|  | **Male** | | |  | **Female** | | |  | **age<60** | | |  | **age>60** | | |  |
| --- | --- | --- | --- | --- | --- | --- | --- | --- | --- | --- | --- | --- | --- | --- | --- | --- |
|  | **Total No.** | **No. of events** | **Hazard ratio (95%CI)** |  | **Total No.** | **No. of events** | **Hazard ratio (95%CI)** |  | **Total No.** | **No. of events** | **Hazard ratio (95%CI)** |  | **Total No.** | **No. of events** | **Hazard ratio (95%CI)** | |
| **Maternal smoking around birth** | | |  |  |  |  |  |  |  |  |  |  |  |  |  | |
| No | 135 953 | 494 | 1.00 (REF) |  | 169 058 | 319 | 1.00 (REF) |  | 172 374 | 260 | 1.00 (REF) |  | 132 637 | 553 | 1.00 (REF) | |
| Yes | 58 812 | 194 | 0.92 (0.78;1.09) |  | 67 302 | 155 | 1.30 (1.07;1.57) |  | 77 264 | 138 | 1.07 (0.86;1.32) |  | 48 850 | 211 | 1.05 (0.90;1.23) | |
| **Part of a multiple birth** | |  |  |  |  |  |  |  |  |  |  |  |  |  |  | |
| No | 218 518 | 810 | 1.00 (REF) |  | 261 601 | 523 | 1.00 (REF) |  | 273 422 | 427 | 1.00 (REF) |  | 206 697 | 906 | 1.00 (REF) | |
| Yes | 5 005 | 11 | 0.62 (0.34;1.13) |  | 6 187 | 14 | 1.22 (0.71;2.11) |  | 6 544 | 14 | 1.31 (0.76;2.27) |  | 4 648 | 11 | 0.59 (0.32;1.08) | |
| **Birth weight** | |  |  |  |  |  |  |  |  |  |  |  |  |  |  | |
| <2.5kg | 8 065 | 24 | 1.00 (REF) |  | 20 073 | 38 | 1.00 (REF) |  | 16 517 | 29 | 1.00 (REF) |  | 11 621 | 33 | 1.00 (REF) | |
| 2.5-4kg | 79 292 | 269 | 1.10 (0.72;1.67) |  | 130 446 | 227 | 1.03 (0.72;1.46) |  | 137 395 | 187 | 0.77 (0.51;1.15) |  | 72 343 | 309 | 1.29 (0.90;1.86) | |
| >4kg | 19 872 | 75 | 1.08 (0.68;1.73) |  | 18 217 | 34 | 0.97 (0.60;1.56) |  | 22 591 | 49 | 1.02 (0.63;1.65) |  | 15 498 | 60 | 1.03 (0.67;1.59) | |
| **Breastfed as a baby** | |  |  |  |  |  |  |  |  |  |  |  |  |  |  | |
| No | 39 799 | 124 | 1.00 (REF) |  | 65 945 | 106 | 1.00 (REF) |  | 75 388 | 113 | 1.00 (REF) |  | 30 356 | 117 | 1.00 (REF) | |
| Yes | 122 348 | 454 | 0.93 (0.76;1.14) |  | 154 106 | 316 | 1.07 (0.85;1.34) |  | 157 240 | 247 | 0.90 (0.71;1.13) |  | 119 214 | 523 | 1.06 (0.87;1.30) | |
| **Comparative body size at age 10** | | |  |  |  |  |  |  |  |  |  |  |  |  |  | |
| Thinner | 77 929 | 281 | 1.00 (REF) |  | 85 083 | 147 | 1.00 (REF) |  | 92 825 | 143 | 1.00 (REF) |  | 70 187 | 285 | 1.00 (REF) | |
| About average | 113 615 | 406 | 0.96 (0.82;1.12) |  | 134 971 | 273 | 1.17 (0.96;1.44) |  | 138 472 | 206 | 0.99 (0.79;1.22) |  | 110 114 | 473 | 1.05 (0.91;1.22) | |
| Larger | 30 417 | 123 | 1.13 (0.91;1.40) |  | 47 418 | 110 | 1.42 (1.11;1.83) |  | 47 910 | 86 | 1.18 (0.90;1.54) |  | 29 925 | 147 | 1.27 (1.04;1.55) | |
| **Comparative height size at age 10** | | |  |  |  |  |  |  |  |  |  |  |  |  |  | |
| Shorter | 43 596 | 124 | 1.00 (REF) |  | 56 582 | 106 | 1.00 (REF) |  | 57 839 | 74 | 1.00 (REF) |  | 42 339 | 156 | 1.00 (REF) | |
| About average | 123 705 | 451 | 1.28 (1.05;1.56) |  | 142 407 | 278 | 1.01 (0.80;1.27) |  | 149 754 | 219 | 1.13 (0.87;1.48) |  | 116 358 | 510 | 1.16 (0.97;1.39) | |
| Taller | 56 144 | 242 | 1.50 (1.21;1.87) |  | 67 632 | 147 | 1.17 (0.91;1.50) |  | 71 899 | 145 | 1.55 (1.16;2.05) |  | 51 877 | 244 | 1.25 (1.02;1.53) | |
| **Smoking age** | |  |  |  |  |  |  |  |  |  |  |  |  |  |  | |
| No smoking | 110 959 | 335 | 1.00 (REF) |  | 161 570 | 302 | 1.00 (REF) |  | 165 657 | 228 | 1.00 (REF) |  | 106 872 | 409 | 1.00 (REF) | |
| 5-14 | 16 590 | 85 | 1.62 (1.27;2.05) |  | 8 859 | 14 | 1.01 (0.59;1.73) |  | 15 614 | 36 | 1.46 (1.02;2.08) |  | 9 835 | 63 | 1.47 (1.12;1.92) | |
| 15-18 | 50 277 | 219 | 1.21 (1.02;1.43) |  | 42 684 | 91 | 1.06 (0.84;1.34) |  | 46 416 | 85 | 1.15 (0.90;1.48) |  | 46 545 | 225 | 1.15 (0.97;1.35) | |
| >18 | 18 449 | 62 | 0.96 (0.73;1.26) |  | 21 470 | 52 | 1.13 (0.84;1.52) |  | 19 382 | 34 | 1.19 (0.83;1.71) |  | 20 537 | 80 | 0.96 (0.76;1.23) | |

^*^ Adjusted for age, sex, region of residence, ethnicity, maternal smoking, multiple births, birth weight, being breastfed, body size at age 10, height size at age 10, and age at smoking initiation simultaneously.

**Supplementary Table 9.** The associations between early-life exposures and risk of Hodgkin's lymphoma in the UK biobank cohort stratified by sex and age

|  | **Male** | | |  | **Female** | | |  | **age<60** | | |  | **age>60** | | |  |
| --- | --- | --- | --- | --- | --- | --- | --- | --- | --- | --- | --- | --- | --- | --- | --- | --- |
|  | **Total No.** | **No. of events** | **Hazard ratio (95%CI)** |  | **Total No.** | **No. of events** | **Hazard ratio (95%CI)** |  | **Total No.** | **No. of events** | **Hazard ratio (95%CI)** |  | **Total No.** | **No. of events** | **Hazard ratio (95%CI)** | |
| **Maternal smoking around birth** | | |  |  |  |  |  |  |  |  |  |  |  |  |  | |
| No | 136 240 | 46 | 1.00 (REF) |  | 169 180 | 45 | 1.00 (REF) |  | 172 385 | 46 | 1.00 (REF) |  | 133 035 | 45 | 1.00 (REF) | |
| Yes | 58 900 | 28 | 1.44 (0.89;2.33) |  | 67 377 | 17 | 0.92 (0.52;1.63) |  | 77 282 | 23 | 1.12 (0.67;1.88) |  | 48 995 | 22 | 1.27 (0.76;2.13) | |
| **Part of a multiple birth** | |  |  |  |  |  |  |  |  |  |  |  |  |  |  | |
| No | 218 976 | 83 | 1.00 (REF) |  | 261 817 | 71 | 1.00 (REF) |  | 273 446 | 75 | 1.00 (REF) |  | 207 347 | 79 | 1.00 (REF) | |
| Yes | 5 012 |  | 0.00 (0.00;0.00) |  | 6 198 | 3 | 2.15 (0.64;7.18) |  | 6 552 | 2 | 1.43 (0.34;5.97) |  | 4 658 | 1 | 0.63 (0.09;4.64) | |
| **Birth weight** | |  |  |  |  |  |  |  |  |  |  |  |  |  |  | |
| <2.5kg | 8 079 | 1 | 1.00 (REF) |  | 20 094 | 4 | 1.00 (REF) |  | 16 526 | 2 | 1.00 (REF) |  | 11 647 | 3 | 1.00 (REF) | |
| 2.5-4kg | 79 435 | 29 | 2.88 (0.39;21.25) |  | 130 522 | 36 | 1.56 (0.53;4.60) |  | 137 398 | 35 | 2.33 (0.55;9.97) |  | 72 559 | 30 | 1.50 (0.45;5.02) | |
| >4kg | 19 900 | 11 | 4.16 (0.53;32.44) |  | 18 221 | 11 | 3.19 (0.96;10.60) |  | 22 590 | 9 | 3.62 (0.75;17.40) |  | 15 531 | 13 | 2.64 (0.73;9.56) | |
| **Breastfed as a baby** | |  |  |  |  |  |  |  |  |  |  |  |  |  |  | |
| No | 39 855 | 13 | 1.00 (REF) |  | 65 972 | 18 | 1.00 (REF) |  | 75 390 | 19 | 1.00 (REF) |  | 30 437 | 12 | 1.00 (REF) | |
| Yes | 122 607 | 44 | 0.92 (0.49;1.74) |  | 154 247 | 45 | 0.98 (0.56;1.71) |  | 157 250 | 49 | 1.07 (0.62;1.84) |  | 119 604 | 40 | 0.82 (0.43;1.57) | |
| **Comparative body size at age 10** | | |  |  |  |  |  |  |  |  |  |  |  |  |  | |
| Thinner | 78 096 | 26 | 1.00 (REF) |  | 85 125 | 22 | 1.00 (REF) |  | 92 830 | 30 | 1.00 (REF) |  | 70 391 | 18 | 1.00 (REF) | |
| About average | 113 844 | 42 | 1.23 (0.75;2.03) |  | 135 092 | 37 | 1.05 (0.61;1.79) |  | 138 482 | 32 | 0.74 (0.45;1.24) |  | 110 454 | 47 | 1.79 (1.03;3.11) | |
| Larger | 30 476 | 13 | 1.36 (0.69;2.65) |  | 47 474 | 15 | 1.14 (0.59;2.21) |  | 47 920 | 13 | 0.82 (0.42;1.58) |  | 30 030 | 15 | 2.01 (1.01;4.02) | |
| **Comparative height size at age 10** | | |  |  |  |  |  |  |  |  |  |  |  |  |  | |
| Shorter | 43 657 | 24 | 1.00 (REF) |  | 56 628 | 14 | 1.00 (REF) |  | 57 843 | 18 | 1.00 (REF) |  | 42 442 | 20 | 1.00 (REF) | |
| About average | 123 956 | 35 | 0.48 (0.28;0.82) |  | 142 545 | 36 | 0.98 (0.53;1.84) |  | 149 766 | 33 | 0.73 (0.40;1.30) |  | 116 735 | 38 | 0.61 (0.35;1.06) | |
| Taller | 56 295 | 22 | 0.67 (0.37;1.20) |  | 67 674 | 24 | 1.30 (0.67;2.52) |  | 71 914 | 24 | 1.04 (0.56;1.93) |  | 52 055 | 22 | 0.78 (0.42;1.44) | |
| **Smoking age** | |  |  |  |  |  |  |  |  |  |  |  |  |  |  | |
| No smoking | 111 133 | 35 | 1.00 (REF) |  | 161 672 | 42 | 1.00 (REF) |  | 165 646 | 41 | 1.00 (REF) |  | 107 159 | 36 | 1.00 (REF) | |
| 5-14 | 16 648 | 8 | 1.47 (0.68;3.17) |  | 8 859 | 5 | 2.22 (0.87;5.66) |  | 15 626 | 7 | 1.84 (0.82;4.16) |  | 9 881 | 6 | 1.51 (0.63;3.64) | |
| 15-18 | 50 404 | 22 | 1.31 (0.76;2.25) |  | 42 724 | 13 | 1.10 (0.59;2.05) |  | 46 431 | 14 | 1.18 (0.64;2.19) |  | 46 697 | 21 | 1.20 (0.70;2.08) | |
| >18 | 18 480 | 5 | 0.82 (0.32;2.11) |  | 21 497 | 8 | 1.29 (0.60;2.76) |  | 19 380 | 7 | 1.43 (0.64;3.20) |  | 20 597 | 6 | 0.81 (0.34;1.93) | |

^*^ Adjusted for age, sex, region of residence, ethnicity, maternal smoking, multiple births, birth weight, being breastfed, body size at age 10, height size at age 10, and age at smoking initiation simultaneously.

**Supplementary Table 10.** The associations between early-life exposures and risk of Non-Hodgkin's lymphoma in the UK biobank cohort stratified by sex and age

|  | **Male** | | |  | **Female** | | |  | **age<60** | | |  | **age>60** | | |  |
| --- | --- | --- | --- | --- | --- | --- | --- | --- | --- | --- | --- | --- | --- | --- | --- | --- |
|  | **Total No.** | **No. of events** | **Hazard ratio (95%CI)** |  | **Total No.** | **No. of events** | **Hazard ratio (95%CI)** |  | **Total No.** | **No. of events** | **Hazard ratio (95%CI)** |  | **Total No.** | **No. of events** | **Hazard ratio (95%CI)** | |
| **Maternal smoking around birth** | | |  |  |  |  |  |  |  |  |  |  |  |  |  | |
| No | 135 337 | 711 | 1.00 (REF) |  | 168 396 | 634 | 1.00 (REF) |  | 171 868 | 444 | 1.00 (REF) |  | 131 865 | 901 | 1.00 (REF) | |
| Yes | 58 495 | 329 | 1.11 (0.97;1.27) |  | 67 076 | 257 | 1.07 (0.92;1.24) |  | 77 027 | 236 | 1.12 (0.96;1.32) |  | 48 544 | 350 | 1.08 (0.95;1.22) | |
| **Part of a multiple birth** | |  |  |  |  |  |  |  |  |  |  |  |  |  |  | |
| No | 217 510 | 1 164 | 1.00 (REF) |  | 260 606 | 1 005 | 1.00 (REF) |  | 272 591 | 752 | 1.00 (REF) |  | 205 525 | 1 417 | 1.00 (REF) | |
| Yes | 4 970 | 31 | 1.20 (0.83;1.73) |  | 6 158 | 28 | 1.22 (0.83;1.79) |  | 6 530 | 19 | 1.04 (0.65;1.66) |  | 4 598 | 40 | 1.31 (0.95;1.82) | |
| **Birth weight** | |  |  |  |  |  |  |  |  |  |  |  |  |  |  | |
| <2.5kg | 8 023 | 43 | 1.00 (REF) |  | 20 004 | 78 | 1.00 (REF) |  | 16 474 | 49 | 1.00 (REF) |  | 11 553 | 72 | 1.00 (REF) | |
| 2.5-4kg | 78 989 | 352 | 0.90 (0.65;1.25) |  | 129 961 | 455 | 0.96 (0.75;1.24) |  | 136 986 | 340 | 0.84 (0.61;1.14) |  | 71 964 | 467 | 1.03 (0.79;1.32) | |
| >4kg | 19 763 | 110 | 1.02 (0.71;1.47) |  | 18 155 | 67 | 0.92 (0.66;1.29) |  | 22 524 | 70 | 0.97 (0.66;1.41) |  | 15 394 | 107 | 1.01 (0.74;1.38) | |
| **Breastfed as a baby** | |  |  |  |  |  |  |  |  |  |  |  |  |  |  | |
| No | 39 647 | 171 | 1.00 (REF) |  | 65 741 | 194 | 1.00 (REF) |  | 75 201 | 170 | 1.00 (REF) |  | 30 187 | 195 | 1.00 (REF) | |
| Yes | 121 785 | 643 | 0.98 (0.82;1.16) |  | 153 502 | 608 | 1.15 (0.97;1.35) |  | 156 734 | 450 | 1.15 (0.96;1.38) |  | 118 553 | 801 | 1.01 (0.86;1.18) | |
| **Comparative body size at age 10** | | |  |  |  |  |  |  |  |  |  |  |  |  |  | |
| Thinner | 77 604 | 382 | 1.00 (REF) |  | 84 757 | 304 | 1.00 (REF) |  | 92 569 | 236 | 1.00 (REF) |  | 69 792 | 450 | 1.00 (REF) | |
| About average | 113 050 | 628 | 1.11 (0.97;1.26) |  | 134 424 | 555 | 1.11 (0.96;1.28) |  | 138 059 | 373 | 1.02 (0.86;1.20) |  | 109 415 | 810 | 1.16 (1.03;1.30) | |
| Larger | 30 253 | 184 | 1.29 (1.08;1.53) |  | 47 262 | 168 | 1.02 (0.84;1.23) |  | 47 729 | 156 | 1.26 (1.03;1.55) |  | 29 786 | 196 | 1.06 (0.90;1.26) | |
| **Comparative height size at age 10** | | |  |  |  |  |  |  |  |  |  |  |  |  |  | |
| Shorter | 43 385 | 205 | 1.00 (REF) |  | 56 405 | 174 | 1.00 (REF) |  | 57 686 | 128 | 1.00 (REF) |  | 42 104 | 251 | 1.00 (REF) | |
| About average | 123 135 | 663 | 1.10 (0.94;1.29) |  | 141 831 | 574 | 1.28 (1.08;1.52) |  | 149 254 | 440 | 1.31 (1.07;1.60) |  | 115 712 | 797 | 1.11 (0.97;1.29) | |
| Taller | 55 873 | 331 | 1.24 (1.04;1.48) |  | 67 363 | 276 | 1.35 (1.12;1.64) |  | 71 702 | 201 | 1.25 (1.00;1.57) |  | 51 534 | 406 | 1.31 (1.12;1.54) | |
| **Smoking age** | |  |  |  |  |  |  |  |  |  |  |  |  |  |  | |
| No smoking | 110 407 | 550 | 1.00 (REF) |  | 160 961 | 619 | 1.00 (REF) |  | 165 150 | 444 | 1.00 (REF) |  | 106 218 | 725 | 1.00 (REF) | |
| 5-14 | 16 523 | 111 | 1.26 (1.03;1.55) |  | 8 830 | 26 | 0.92 (0.62;1.37) |  | 15 573 | 53 | 1.20 (0.90;1.60) |  | 9 780 | 84 | 1.16 (0.92;1.46) | |
| 15-18 | 50 034 | 292 | 0.98 (0.85;1.13) |  | 42 485 | 183 | 1.07 (0.90;1.26) |  | 46 266 | 135 | 0.99 (0.81;1.20) |  | 46 253 | 340 | 1.01 (0.89;1.16) | |
| >18 | 18 349 | 108 | 1.02 (0.83;1.26) |  | 21 396 | 78 | 0.85 (0.67;1.07) |  | 19 320 | 60 | 1.11 (0.85;1.45) |  | 20 425 | 126 | 0.87 (0.72;1.06) | |

^*^ Adjusted for age, sex, region of residence, ethnicity, maternal smoking, multiple births, birth weight, being breastfed, body size at age 10, height size at age 10, and age at smoking initiation simultaneously.

**Supplementary Table 11.** The associations between early-life exposures and risk of multiple myeloma in the UK biobank cohort stratified by sex and age

|  | **Male** | | |  | **Female** | | |  | **age<60** | | |  | **age>60** | | |  |
| --- | --- | --- | --- | --- | --- | --- | --- | --- | --- | --- | --- | --- | --- | --- | --- | --- |
|  | **Total No.** | **No. of events** | **Hazard ratio (95%CI)** |  | **Total No.** | **No. of events** | **Hazard ratio (95%CI)** |  | **Total No.** | **No. of events** | **Hazard ratio (95%CI)** |  | **Total No.** | **No. of events** | **Hazard ratio (95%CI)** | |
| **Maternal smoking around birth** | | |  |  |  |  |  |  |  |  |  |  |  |  |  | |
| No | 136000 | 357 | 1.00 (REF) |  | 169025 | 292 | 1.00 (REF) |  | 172384 | 201 | 1.00 (REF) |  | 132641 | 448 | 1.00 (REF) | |
| Yes | 58845 | 132 | 0.92 (0.75;1.13) |  | 67353 | 79 | 0.78 (0.61;1.01) |  | 77312 | 71 | 0.78 (0.59;1.03) |  | 48886 | 140 | 0.91 (0.75;1.11) | |
| **Part of a multiple birth** | |  |  |  |  |  |  |  |  |  |  |  |  |  |  | |
| No | 218635 | 560 | 1.00 (REF) |  | 261623 | 406 | 1.00 (REF) |  | 273474 | 299 | 1.00 (REF) |  | 206784 | 667 | 1.00 (REF) | |
| Yes | 5006 | 7 | 0.59 (0.28;1.26) |  | 6183 | 16 | 1.80 (1.07;3.03) |  | 6550 | 6 | 0.89 (0.39;2.03) |  | 4639 | 17 | 1.20 (0.73;1.97) | |
| **Birth weight** | |  |  |  |  |  |  |  |  |  |  |  |  |  |  | |
| <2.5kg | 8071 | 14 | 1.00 (REF) |  | 20075 | 30 | 1.00 (REF) |  | 16530 | 14 | 1.00 (REF) |  | 11616 | 30 | 1.00 (REF) | |
| 2.5-4kg | 79353 | 160 | 1.19 (0.68;2.07) |  | 130443 | 188 | 1.14 (0.76;1.70) |  | 137418 | 125 | 1.05 (0.60;1.86) |  | 72378 | 223 | 1.17 (0.79;1.74) | |
| >4kg | 19887 | 49 | 1.33 (0.73;2.44) |  | 18217 | 29 | 1.12 (0.66;1.90) |  | 22602 | 36 | 1.64 (0.87;3.09) |  | 15502 | 42 | 0.96 (0.59;1.56) | |
| **Breastfed as a baby** | |  |  |  |  |  |  |  |  |  |  |  |  |  |  | |
| No | 39829 | 77 | 1.00 (REF) |  | 65955 | 82 | 1.00 (REF) |  | 75408 | 76 | 1.00 (REF) |  | 30376 | 83 | 1.00 (REF) | |
| Yes | 122422 | 304 | 0.93 (0.72;1.20) |  | 154109 | 256 | 1.00 (0.77;1.29) |  | 157270 | 177 | 0.85 (0.64;1.12) |  | 119261 | 383 | 1.06 (0.83;1.35) | |
| **Comparative body size at age 10** | | |  |  |  |  |  |  |  |  |  |  |  |  |  | |
| Thinner | 77969 | 197 | 1.00 (REF) |  | 85074 | 126 | 1.00 (REF) |  | 92848 | 99 | 1.00 (REF) |  | 70195 | 224 | 1.00 (REF) | |
| About average | 113661 | 289 | 0.99 (0.82;1.19) |  | 134984 | 215 | 1.10 (0.88;1.38) |  | 138491 | 147 | 1.00 (0.77;1.29) |  | 110154 | 357 | 1.04 (0.87;1.23) | |
| Larger | 30441 | 80 | 1.12 (0.86;1.45) |  | 47429 | 79 | 1.22 (0.92;1.62) |  | 47916 | 62 | 1.22 (0.89;1.68) |  | 29954 | 97 | 1.11 (0.87;1.41) | |
| **Comparative height size at age 10** | | |  |  |  |  |  |  |  |  |  |  |  |  |  | |
| Shorter | 43589 | 113 | 1.00 (REF) |  | 56587 | 84 | 1.00 (REF) |  | 57845 | 54 | 1.00 (REF) |  | 42331 | 143 | 1.00 (REF) | |
| About average | 123775 | 304 | 0.91 (0.73;1.14) |  | 142413 | 221 | 1.01 (0.79;1.31) |  | 149767 | 159 | 1.09 (0.79;1.49) |  | 116421 | 366 | 0.91 (0.74;1.10) | |
| Taller | 56200 | 145 | 0.98 (0.77;1.26) |  | 67638 | 112 | 1.11 (0.83;1.48) |  | 71938 | 87 | 1.23 (0.87;1.73) |  | 51900 | 170 | 0.96 (0.77;1.21) | |
| **Smoking age** | |  |  |  |  |  |  |  |  |  |  |  |  |  |  | |
| No smoking | 110964 | 271 | 1.00 (REF) |  | 161556 | 251 | 1.00 (REF) |  | 165668 | 170 | 1.00 (REF) |  | 106852 | 352 | 1.00 (REF) | |
| 5-14 | 16631 | 34 | 0.79 (0.56;1.14) |  | 8863 | 6 | 0.61 (0.27;1.38) |  | 15628 | 17 | 1.04 (0.63;1.71) |  | 9866 | 23 | 0.66 (0.43;1.01) | |
| 15-18 | 50327 | 129 | 0.86 (0.69;1.06) |  | 42695 | 68 | 1.08 (0.82;1.41) |  | 46433 | 54 | 1.04 (0.76;1.41) |  | 46589 | 143 | 0.88 (0.72;1.08) | |
| >18 | 18445 | 52 | 0.97 (0.72;1.31) |  | 21481 | 38 | 1.05 (0.74;1.48) |  | 19388 | 23 | 1.08 (0.70;1.67) |  | 20538 | 67 | 0.95 (0.73;1.24) | |

^*^ Adjusted for age, sex, region of residence, ethnicity, maternal smoking, multiple births, birth weight, being breastfed, body size at age 10, height size at age 10, and age at smoking initiation simultaneously.

**Supplementary Table 12.** Risk of hematological malignancies in the White population of UK biobank cohort by early-life exposures

|  | **Hematological Malignancies** | | |
| --- | --- | --- | --- |
|  | **Total number** | **NO of events** | **Hazard ratio (95%CI)** |
| **Maternal smoking around birth** |  |  |  |
| No | 277671 | 2661 | 1.00 (REF) |
| Yes | 122620 | 1127 | 1.02 (0.95;1.10) |
| **Part of a multiple birth** |  |  |  |
| No | 447822 | 4298 | 1.00 (REF) |
| Yes | 10535 | 100 | 1.04 (0.85;1.27) |
| **Birth weight** |  |  |  |
| <2.5kg | 26693 | 221 | 1.00 (REF) |
| 2.5-4kg | 200968 | 1615 | 1.00 (0.87;1.16) |
| >4kg | 36571 | 374 | 1.08 (0.91;1.28) |
| **Breastfed as a baby** |  |  |  |
| No | 102183 | 755 | 1.00 (REF) |
| Yes | 252198 | 2449 | 1.01 (0.93;1.10) |
| **Comparative body size at age 10** |  |  |  |
| Thinner | 150668 | 1374 | 1.00 (REF) |
| About average | 73167 | 716 | **1.16 (1.06;1.28)** |
| Plumper | 233782 | 2287 | 1.06 (0.99;1.14) |
| **Comparative height size at age 10** |  |  |  |
| Shorter | 93665 | 776 | 1.00 (REF) |
| About average | 116630 | 1207 | **1.23 (1.13;1.35)** |
| Taller | 247700 | 2399 | **1.12 (1.03;1.22)** |
| **Smoking age** |  |  |  |
| No smoking | 250443 | 2212 | 1.00 (REF) |
| 5-14 | 24373 | 271 | **1.18 (1.04;1.34)** |
| 15-18 | 89292 | 960 | 1.03 (0.95;1.11) |
| >18 | 37031 | 376 | 0.99 (0.89;1.10) |

^*^ The Model adjusted for maternal smoking, multiple births, birth weight, being breastfed, body size at age 10, height size at age 10, and age at smoking initiation simultaneously. Statistically significant results are bolded. CI confidence interval, HR hazard ratio.

**Supplementary Table 13.** Risk of hematological malignancies in the White population of UK biobank cohort by early-life exposures

|  | **Total number** | **Leukemia** | | **Hodgkin's lymphoma** | | **Non-Hodgkin's lymphoma** | | **Multiple myeloma** | |
| --- | --- | --- | --- | --- | --- | --- | --- | --- | --- |
|  |  | **NO of events** | **Hazard ratio**  **(95%CI)** | **NO of events** | **Hazard ratio**  **(95%CI)** | **NO of events** | **Hazard ratio**  **(95%CI)** | **NO of events** | **Hazard ratio**  **(95%CI)** |
| **Maternal smoking around birth** | | |  |  |  |  |  |  |  |
| No | 277671 | 781 | 1.00 (REF) | 82 | 1.00 (REF) | 1274 | 1.00 (REF) | 590 | 1.00 (REF) |
| Yes | 122620 | 348 | 1.07 (0.94;1.21) | 45 | 1.22 (0.84;1.77) | 575 | 1.08 (0.98;1.20) | 204 | **0.86 (0.73;1.00)** |
| **Part of a multiple birth** |  |  |  |  |  |  |  |  |  |
| No | 447822 | 1296 | 1.00 (REF) | 145 | 1.00 (REF) | 2084 | 1.00 (REF) | 903 | 1.00 (REF) |
| Yes | 10535 | 23 | 0.80 (0.52;1.21) | 3 | 1.06 (0.33;3.41) | 56 | 1.19 (0.90;1.56) | 20 | 1.00 (0.64;1.58) |
| **Birth weight** |  |  |  |  |  |  |  |  |  |
| <2.5kg | 26693 | 61 | 1.00 (REF) | 5 | 1.00 (REF) | 117 | 1.00 (REF) | 43 | 1.00 (REF) |
| 2.5-4kg | 200968 | 486 | 1.03 (0.78;1.35) | 62 | 1.78 (0.70;4.52) | 781 | 0.94 (0.77;1.15) | 335 | 1.08 (0.78;1.50) |
| >4kg | 36571 | 108 | 1.02 (0.74;1.41) | 21 | **3.02 (1.11;8.24)** | 175 | 1.00 (0.79;1.27) | 78 | 1.17 (0.80;1.72) |
| **Breastfed as a baby** |  |  |  |  |  |  |  |  |  |
| No | 102183 | 226 | 1.00 (REF) | 31 | 1.00 (REF) | 360 | 1.00 (REF) | 152 | 1.00 (REF) |
| Yes | 252198 | 738 | 0.99 (0.85;1.15) | 80 | 0.90 (0.59;1.37) | 1190 | 1.06 (0.94;1.19) | 513 | 0.99 (0.82;1.19) |
| **Comparative body size at age 10** | |  |  |  |  |  |  |  |  |
| Thinner | 150668 | 412 | 1.00 (REF) | 45 | 1.00 (REF) | 655 | 1.00 (REF) | 298 | 1.00 (REF) |
| About average | 73167 | 228 | **1.25 (1.06;1.47)** | 26 | 1.22 (0.75;1.99) | 339 | **1.14 (1.00;1.30)** | 148 | 1.15 (0.95;1.41) |
| Plumper | 233782 | 661 | 1.03 (0.91;1.17) | 75 | 1.15 (0.79;1.68) | 1146 | **1.11 (1.01;1.23)** | 475 | 1.02 (0.88;1.18) |
| **Comparative height size at age 10** | |  |  |  |  |  |  |  |  |
| Shorter | 93665 | 222 | 1.00 (REF) | 38 | 1.00 (REF) | 366 | 1.00 (REF) | 183 | 1.00 (REF) |
| About average | 116630 | 376 | **1.34 (1.13;1.58)** | 44 | 0.86 (0.56;1.34) | 587 | **1.29 (1.13;1.47)** | 236 | 1.01 (0.83;1.23) |
| Taller | 247700 | 713 | **1.17 (1.00;1.36)** | 64 | **0.60 (0.40;0.90)** | 1188 | **1.18 (1.04;1.32)** | 495 | 0.97 (0.82;1.15) |
| **Smoking age** |  |  |  |  |  |  |  |  |  |
| No smoking | 250443 | 611 | 1.00 (REF) | 71 | 1.00 (REF) | 1113 | 1.00 (REF) | 474 | 1.00 (REF) |
| 5-14 | 24373 | 98 | **1.48 (1.19;1.83)** | 13 | 1.78 (0.97;3.24) | 135 | **1.20 (1.00;1.44)** | 39 | 0.78 (0.56;1.09) |
| 15-18 | 89292 | 304 | **1.15 (1.00;1.32)** | 35 | 1.28 (0.85;1.92) | 466 | 1.01 (0.91;1.13) | 189 | 0.92 (0.77;1.09) |
| >18 | 37031 | 111 | 1.05 (0.85;1.28) | 13 | 1.17 (0.64;2.11) | 179 | 0.95 (0.81;1.11) | 86 | 1.02 (0.81;1.29) |

^*^ The Model adjusted for maternal smoking, multiple births, birth weight, being breastfed, body size at age 10, height size at age 10, and age at smoking initiation simultaneously. Statistically significant results are bolded. CI confidence interval, HR hazard ratio.

**Supplementary Table 14.** Mendelian randomization analysis for the associations between early-life exposures and risk of hematological malignancies

| Exposure | Outcome | Method | No. SNP | beta | se | p-value |
| --- | --- | --- | --- | --- | --- | --- |
| Birth weight | AML | Inverse variance weighted | 61 | 0.348096 | 0.534326 | 0.514744 |
| Birth weight | AML | MR Egger | 61 | -0.65463 | 1.540855 | 0.672494 |
| Birth weight | AML | Weighted median | 61 | 0.455867 | 0.778947 | 0.55839 |
| Height at age 10 | AML | Inverse variance weighted | 634 | 0.321418 | 0.28158 | 0.253669 |
| Height at age 10 | AML | MR Egger | 634 | 0.50894 | 0.705882 | 0.471177 |
| Height at age 10 | AML | Weighted median | 634 | 0.467221 | 0.439872 | 0.288157 |
| Age at smoking initiation | AML | Inverse variance weighted | 10 | -2.7712 | 2.025122 | 0.171184 |
| Age at smoking initiation | AML | MR Egger | 10 | -24.5709 | 10.41484 | 0.046014 |
| Age at smoking initiation | AML | Weighted median | 10 | -3.20744 | 2.75539 | 0.244399 |
| Body size at age 10 | AML | Inverse variance weighted | 194 | -0.40286 | 0.515004 | 0.434075 |
| Body size at age 10 | AML | MR Egger | 194 | 0.635627 | 1.184647 | 0.592197 |
| Body size at age 10 | AML | Weighted median | 194 | -0.06629 | 0.848465 | 0.937726 |
| Birth weight | CLL | Inverse variance weighted | 61 | 0.413618 | 0.326531 | 0.205262 |
| Birth weight | CLL | MR Egger | 61 | 0.027549 | 0.944985 | 0.976841 |
| Birth weight | CLL | Weighted median | 61 | 0.294709 | 0.483848 | 0.542461 |
| Height at age 10 | CLL | Inverse variance weighted | 634 | 0.358754 | 0.178171 | 0.044058 |
| Height at age 10 | CLL | MR Egger | 634 | 0.991816 | 0.445211 | 0.026249 |
| Height at age 10 | CLL | Weighted median | 634 | 0.372653 | 0.269062 | 0.16605 |
| Age at smoking initiation | CLL | Inverse variance weighted | 10 | 1.817782 | 1.416256 | 0.199313 |
| Age at smoking initiation | CLL | MR Egger | 10 | -11.7636 | 6.500954 | 0.107971 |
| Age at smoking initiation | CLL | Weighted median | 10 | 1.516198 | 1.695276 | 0.371126 |
| Body size at age 10 | CLL | Inverse variance weighted | 194 | 0.154743 | 0.314142 | 0.622302 |
| Body size at age 10 | CLL | MR Egger | 194 | -0.04402 | 0.722068 | 0.951447 |
| Body size at age 10 | CLL | Weighted median | 194 | -0.02544 | 0.531673 | 0.961835 |
| Birth weight | Hodgkin's lymphoma | Inverse variance weighted | 61 | -0.22457 | 0.290241 | 0.439088 |
| Birth weight | Hodgkin's lymphoma | MR Egger | 61 | -0.49469 | 0.831505 | 0.554166 |
| Birth weight | Hodgkin's lymphoma | Weighted median | 61 | -0.49611 | 0.432348 | 0.251185 |
| Height at age 10 | Hodgkin's lymphoma | Inverse variance weighted | 634 | 0.122677 | 0.152662 | 0.421637 |
| Height at age 10 | Hodgkin's lymphoma | MR Egger | 634 | -0.13213 | 0.380505 | 0.728525 |
| Height at age 10 | Hodgkin's lymphoma | Weighted median | 634 | 0.057121 | 0.235242 | 0.808147 |
| Age at smoking initiation | Hodgkin's lymphoma | Inverse variance weighted | 10 | -1.60056 | 1.495208 | 0.284412 |
| Age at smoking initiation | Hodgkin's lymphoma | MR Egger | 10 | -5.44191 | 8.472171 | 0.538628 |
| Age at smoking initiation | Hodgkin's lymphoma | Weighted median | 10 | -1.27609 | 1.520428 | 0.401301 |
| Body size at age 10 | Hodgkin's lymphoma | Inverse variance weighted | 194 | 0.221027 | 0.291361 | 0.448091 |
| Body size at age 10 | Hodgkin's lymphoma | MR Egger | 194 | 0.298998 | 0.671779 | 0.65676 |
| Body size at age 10 | Hodgkin's lymphoma | Weighted median | 194 | 0.3753 | 0.473576 | 0.42808 |
| Birth weight | Multiple myeloma | Inverse variance weighted | 61 | 0.264834 | 0.336897 | 0.431811 |
| Birth weight | Multiple myeloma | MR Egger | 61 | 1.219128 | 0.974692 | 0.215951 |
| Birth weight | Multiple myeloma | Weighted median | 61 | 0.258783 | 0.50619 | 0.609185 |
| Height at age 10 | Multiple myeloma | Inverse variance weighted | 634 | 0.083746 | 0.173951 | 0.630206 |
| Height at age 10 | Multiple myeloma | MR Egger | 634 | 0.378104 | 0.435893 | 0.38604 |
| Height at age 10 | Multiple myeloma | Weighted median | 634 | 0.183861 | 0.281387 | 0.513493 |
| Age at smoking initiation | Multiple myeloma | Inverse variance weighted | 10 | 0.762881 | 1.341957 | 0.569706 |
| Age at smoking initiation | Multiple myeloma | MR Egger | 10 | 1.877722 | 7.668639 | 0.812731 |
| Age at smoking initiation | Multiple myeloma | Weighted median | 10 | 0.693293 | 1.602041 | 0.665192 |
| Body size at age 10 | Multiple myeloma | Inverse variance weighted | 194 | 0.067208 | 0.324353 | 0.835849 |
| Body size at age 10 | Multiple myeloma | MR Egger | 194 | -0.01796 | 0.746797 | 0.980837 |
| Body size at age 10 | Multiple myeloma | Weighted median | 194 | -0.28099 | 0.570269 | 0.622207 |
| Birth weight | Non-Hodgkin's lymphoma | Inverse variance weighted | 61 | 0.053083 | 0.29553 | 0.857451 |
| Birth weight | Non-Hodgkin's lymphoma | MR Egger | 61 | 0.560627 | 0.858962 | 0.516498 |
| Birth weight | Non-Hodgkin's lymphoma | Weighted median | 61 | 0.157033 | 0.439658 | 0.720963 |
| Height at age 10 | Non-Hodgkin's lymphoma | Inverse variance weighted | 634 | 0.199007 | 0.141453 | 0.159464 |
| Height at age 10 | Non-Hodgkin's lymphoma | MR Egger | 634 | 0.451165 | 0.354381 | 0.203447 |
| Height at age 10 | Non-Hodgkin's lymphoma | Weighted median | 634 | 0.157758 | 0.225014 | 0.483238 |
| Age at smoking initiation | Non-Hodgkin's lymphoma | Inverse variance weighted | 10 | -1.70287 | 0.966855 | 0.078196 |
| Age at smoking initiation | Non-Hodgkin's lymphoma | MR Egger | 10 | -4.58343 | 5.216227 | 0.405189 |
| Age at smoking initiation | Non-Hodgkin's lymphoma | Weighted median | 10 | -2.28758 | 1.25344 | 0.067995 |
| Body size at age 10 | Non-Hodgkin's lymphoma | Inverse variance weighted | 194 | 0.086603 | 0.259782 | 0.738858 |
| Body size at age 10 | Non-Hodgkin's lymphoma | MR Egger | 194 | 0.302413 | 0.598959 | 0.614209 |
| Body size at age 10 | Non-Hodgkin's lymphoma | Weighted median | 194 | -0.16346 | 0.433137 | 0.705891 |

*In the analyses, 61 out of 63, 634 out of 648, 194 out of 204 SNPs for birth weight, height at 10 and body size at 10, respectively, were found in the summary statistics of hematological cancer.


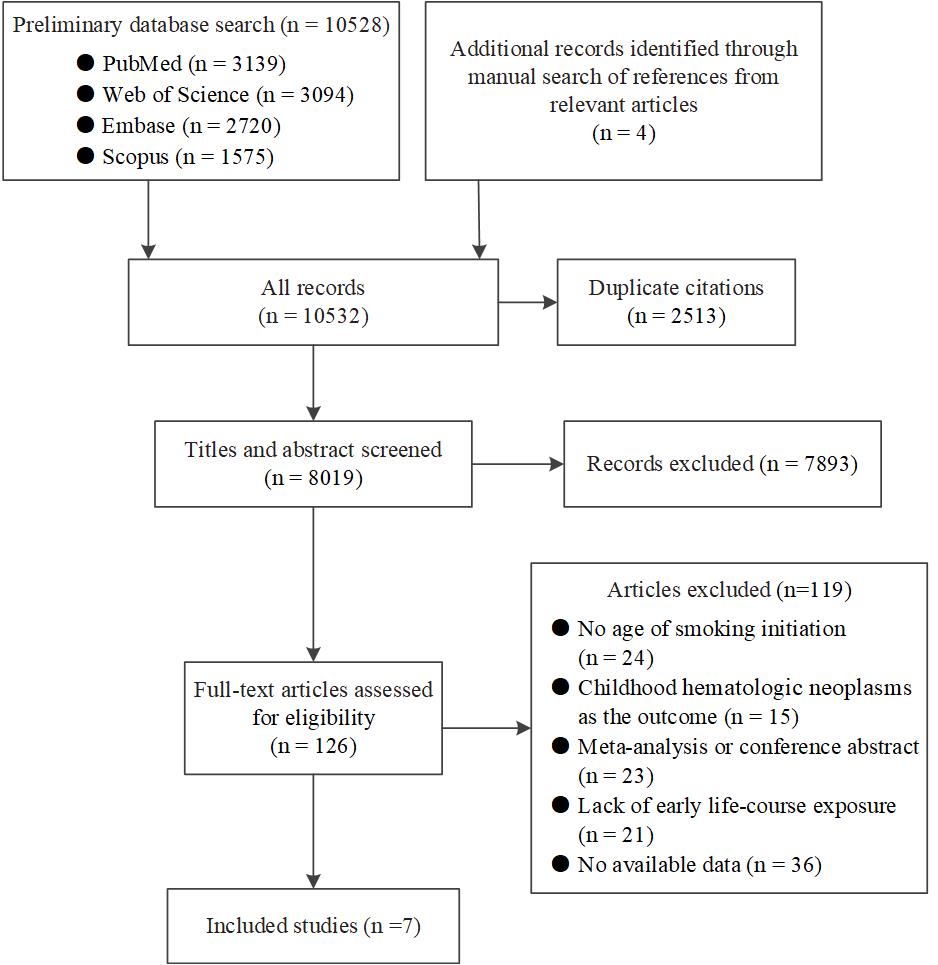


**Supplementary Figure 1** Flow chart for the selection of studies in the meta-analysis
